# Aging induces aberrant state transition kinetics in murine muscle stem cells

**DOI:** 10.1242/dev.183855

**Published:** 2020-05-05

**Authors:** Jacob C. Kimmel, Ara B. Hwang, Annarita Scaramozza, Wallace F. Marshall, Andrew S. Brack

**Affiliations:** 1 Eli and Edythe Broad Center for Regenerative Medicine, University of California, San Francisco, 35 Medical Center Way, San Francisco, CA 94143, USA; 2 Center for Cellular Construction, University of California, San Francisco, San Francisco, CA 94143, USA; 3 Biochemistry & Biophysics, University of California, San Francisco, San Francisco, CA 94143, USA

**Keywords:** Aging, Muscle stem cell, Single cell RNA-seq, Time-lapse imaging, State transition, Stem cell activation

## Abstract

Murine muscle stem cells (MuSCs) experience a transition from quiescence to activation that is required for regeneration, but it remains unknown if the trajectory and dynamics of activation change with age. Here, we use time-lapse imaging and single cell RNA-seq to measure activation trajectories and rates in young and aged MuSCs. We find that the activation trajectory is conserved in aged cells, and we develop effective machine-learning classifiers for cell age. Using cell-behavior analysis and RNA velocity, we find that activation kinetics are delayed in aged MuSCs, suggesting that changes in stem cell dynamics may contribute to impaired stem cell function with age. Intriguingly, we also find that stem cell activation appears to be a random walk-like process, with frequent reversals, rather than a continuous linear progression. These results support a view of the aged stem cell phenotype as a combination of differences in the location of stable cell states and differences in transition rates between them.

## Abstract (precis)

**Highlighted Article:** Aged muscle stem cells display delayed activation dynamics, but retain a youthful activation trajectory, suggesting that changes to cell state dynamics might contribute to aging pathology.

## INTRODUCTION

Stem cells play a keystone role in tissue homeostasis and regeneration in mammalian tissues. During homeostasis, stem cells in multiple systems maintain a noncycling quiescent state (Fuchs, 2009). In the event of injury, quiescent stem cells undergo a dynamic process of activation, generating biomass, restructuring cellular geometry, altering metabolism (Ryall et al., 2015), and entering the cell cycle to produce progenitor daughters (Brack and Rando, 2012). Impaired stem cell activation has been shown to impair regenerative potential in multiple tissues (Megeney et al., 1996; Zou et al., 2018). Likewise, ‘priming’ of activation by systemic signaling factors has been reported to improve regeneration (Rodgers et al., 2014, 2017).

As murine-muscle stem cells (MuSCs) age, the proportion of cells in regenerative states declines, and the overall regenerative capacity of the stem cell pool is greatly diminished (Blau et al., 2015; Brack and Muñoz-Cánoves, 2016). Age-related decline in regenerative potential has been attributed to differences in cell signaling (Bernet et al., 2014; Brack et al., 2007; Conboy et al., 2003; Cosgrove et al., 2014) and proliferative history (Chakkalakal et al., 2012). These differences in regenerative potential between stem cells are traditionally viewed as the result of differences in the characteristics of stable cell phenotypes (Altschuler and Wu, 2010; Blau et al., 2015; Chakkalakal et al., 2012).

However, aged MuSCs have been reported to show impaired activation in multiple studies, suggesting that a defect in activation dynamics may also contribute to impaired regeneration (Brack et al., 2007; Cosgrove et al., 2014; Egerman et al., 2015; Gilbert et al., 2010). Differences in activation with age could be the result of cells taking different paths through state space, or the result of cells moving along the same path at different rates. Impaired activation with age may therefore be explained by one of two models, or a combination of the two.

**Fig. 1.**
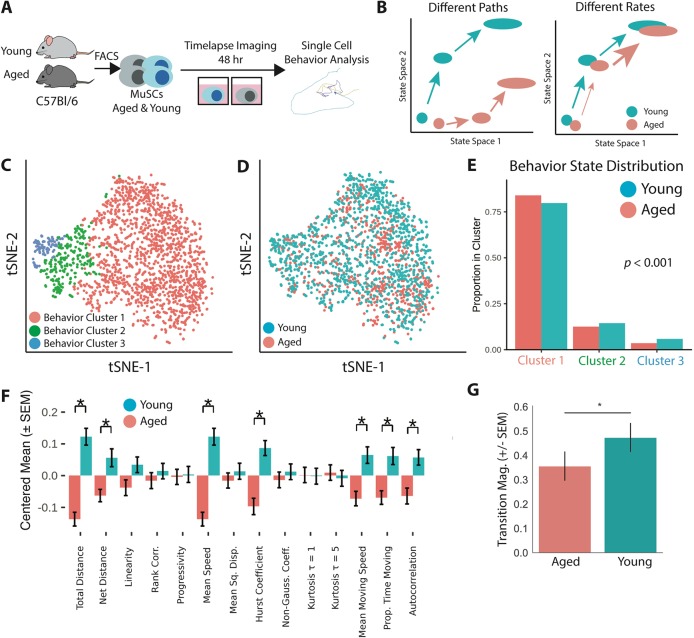
**Aged MuSCs display lower cell motility and delayed activation by single cell behavior analysis.** (A) Experimental schematic. MuSCs were isolated and imaged on a time-lapse microscope for 48 h. Tracking was performed from 10 h to 35 h. (B) Diagram of the different paths and different rates models for age-related decline in muscle stem cell regenerative capacity. (C) t-SNE visualization of cell behavior state space with color overlay of hierarchical clustering identities (aged animals: *n*=742 cells; young animals: *n*=1201 cells). (D) t-SNE visualization of cell ages in cell behavior space. (E) Aged cells display a significant preference for less-motile behavior clusters (χ2 test, age × behavior cluster contingency table, *P*<0.001). (F) Young cells are significantly more motile than aged cells, suggesting that aged cells are delayed in activation. Mean feature values are presented for each age after centering the population mean to μ=0 and scaling the variance to σ^2^=1 (*P<0.05, two-tailed unpaired Student's *t*-test, Holm–Bonferroni corrected). (G) Aged cells have significantly decreased behavior state transition magnitude (two-tailed unpaired Student's *t*-test, *P<0.05), suggesting delayed activation. State transition magnitude in behavior space is measured as the magnitude of the mean transition vector.

In the first model (different paths), the location of cell states is shifted by age, such that aged cells at a particular point in the activation process exhibit different phenotypes than those of young cells at the same point in the process. In the second model (different rates), aged and young cells traverse a similar phenotypic path during activation, but take different amounts of time to reach a given point in the process. In this model, differences in young and aged phenotypes are primarily the result of changes in activation dynamics (Fig. 1B).

In MuSCs, the activation process is canonically characterized by expression of *Myod1* (Grounds et al., 1992; Yablonka-Reuveni and Rivera, 1994), loss of *Spry1* and *Pax7*, and entry into the cell cycle (Shea et al., 2010). Multiple groups have characterized the dynamics of activation at the population level using ensemble assays to measure these molecular markers (Cornelison and Wold, 1997; Fu et al., 2015; Jones et al., 2005; Yablonka-Reuveni and Rivera, 1994; Zhang et al., 2010). Likewise, it has been reported that aged MuSCs show a delayed time to first division relative to young cells, with fewer aged cells forming colonies *in vitro* (Cosgrove et al., 2014; Gilbert et al., 2010). These studies have elucidated many of the molecular players and sequences in MuSC activation and shown that aged cells exhibit a delay in at least one activation hallmark (first division time).

Genomics studies have revealed that MuSC activation is a complex process, affecting many aspects of transcription and cell behavior (Liu et al., 2013). However, it remains unknown how aging affects the progress of activation in MuSCs outside of a small set of molecular markers and binary behavior features (i.e. cell cycle events). Although it is known that aged MuSCs display a delayed cell-cycle entry, for instance, it is unknown if this one feature of cell behavior reflects a broader delay in the activation process across the many transcriptional and cell behavior features involved. Traditional molecular biology tools have also limited investigation to terminal assays, such that activation dynamics in single cells have not been directly observed. In order to disambiguate between the different paths and different rates models of MuSC aging, we require single cell measurements of activation dynamics that capture a broad set of transcriptional and behavioral features.

Single cell analyses in the hematopoietic system identified distinct aged and young transcriptional phenotypes (as in the different paths model), and altered cell cycle kinetics (as in the different rates model) (Kowalczyk et al., 2015), suggesting that both models are plausible in the context of myogenic activation. To investigate each of these possibilities, we use our recently developed cell-behavior analysis platform ‘Heteromotility’ (Kimmel et al., 2018) to quantify phenotypic-state dynamics during MuSC activation in aged and young MuSCs. Multiple groups have demonstrated the value of single cell RNA sequencing (scRNA-seq) to elucidate differences between skeletal muscle cell types and dynamic regulation of myogenic programs following injury (Dell'Orso et al., 2019; Giordani et al., 2019; The Tabula Muris Consortium et al., 2018). We likewise complement our behavioral assay approach with scRNA-seq to map the transcriptional state space of MuSC activation. Leveraging RNA velocity analysis (La Manno et al., 2018), we infer transcriptional-state transition dynamics to pair with state transition dynamics inferred from cell behavior.

In these transcriptional assays, we further investigate differences across age and activation state within the subsets of highly regenerative label-retaining cells (LRCs) and less regenerative non-label retaining cells (nonLRCs). We previously described LRCs and nonLRCs as discrete populations of MuSCs with different proliferative histories and different regenerative potentials (Chakkalakal et al., 2012, 2014). The relative proportions of these populations changes with age, suggesting that age-related changes specific to the LRC or nonLRC compartment may shed light on MuSC aging.

We find that both behavioral and transcriptional-state spaces are continuous across MuSC activation and that measurements of cell heterogeneity are comparable between assays. In aged MuSCs, we find aberrant transition dynamics that lead to significantly delayed activation by both methods. These findings are reflected in a comparison of LRCs to less regenerative nonLRCs, suggesting aberrant transition dynamics might be related more generally to impaired regenerative potential. Identifying genetic pathways that are altered in both aged MuSCs and nonLRCs, we find that biosynthetic processes activate more slowly in both populations. To determine if less regenerative MuSCs occupy different steady states, we trained machine-learning classifiers to discriminate MuSC age and LRC status. Classifiers readily discriminate between MuSC ages and proliferative histories. Our results suggest that aged stem cells display delayed activation kinetics, in addition to subtle differences in the position of activation states.

## RESULTS

### Activation kinetics are delayed in aged MuSCs

Previously, we demonstrated that quantitative measurements of cell motility behavior from time-lapse imaging data are sufficient to resolve states of MuSC activation and state transitions (Kimmel et al., 2018). This approach allows for the direct observation of cell-state transitions during MuSC activation. Cell behavior measurements also have functional relevance in MuSCs, as motility is necessary for MuSCs to translocate to sites of injury.

We applied this cell behavior analysis method to aged and young MuSCs to determine (1) if aged cells occupied distinct behavioral states, and (2) if aged cells exhibit different cell state transition dynamics during activation. MuSCs were isolated from aged (20 months old, *n*=1) and young (3 months old, *n*=1) mice by fluorescence-activated cell sorting (FACS). To ensure the ratio of distinct proliferative histories (Chakkalakal et al., 2012, 2014) in young and aged cells was well represented, we isolated cells after labeling proliferative history developmentally, and sampled representative young and aged cell populations *in silico*. Time-lapse microscopy was performed for 48 h after plating (Fig. 1A). This temporal window captures the early stages of MuSC activation, including the switch from a quiescent *Pax7^+^*/*Myod1^−^* state to a *Myod1^+^* state (Cornelison and Wold, 1997).

We quantified cell behaviors in a 10-35 h window of the time lapse using Heteromotility (Movies 1-4). The visualization of cell behavior state space with t-SNE (van der Maaten and Hinton, 2008) revealed heterogeneous cell behavior states, previously shown to reflect different states of MuSC activation (Fig. 1C) (Kimmel et al., 2018). Hierarchical clustering revealed three cell behavior states (colors in Fig. 1C). Behavior cluster 1 was largely immotile, behavior cluster 2 displayed limited motility behavior, and behavior cluster 3 displayed more extensive and dramatic motility behaviors.

Aged and young cells did not occupy distinct regions in behavioral state space (Fig. 1D). Quantification of the proportion of aged and young cells in each motility state revealed that aged cells show a significant preference for the less motile behavior cluster 1 relative to young cells (Fig. 1E, χ^2^, *P*<0.001). Motility state preferences were consistent across bootstrap samples (Fig. S1A,B) and the influence of age on cluster preference was significant after controlling for proliferative history (logistic regression, *P*<0.01). This result was replicated in a second independent experiment (Fig. S1F). The preference for less motile states among aged cells suggests they exhibit a slower phenotypic change from quiescence to activation.

Comparing individual cell behavior features between aged and young cells confirmed that aged cells are significantly less motile than young cells. Metrics of net motility distance and mean motility speed were significantly higher in young cells (*t*-test, *q*<0.05; Fig. 1F). This result was replicated in a second independent experiment (Fig. S1G).

We next quantified state transition rates for aged and young cells within the motility state space. Aged cells had lower state transition magnitudes (Fig. 1G; *P*<0.05, *t*-test). This result was consistent across random bootstrap samples (Fig. S1C).

Enrichment of aged cells in less activated behavioral states and dampened state transition rates in aged cells indicated that aged cells are delayed in activation relative to young counterparts. We interpret these results as support for the different rates model of MuSC aging noted above, in which aberrant dynamics between states contribute to the functional defects observed in aged MuSCs.

### Transcriptome analysis reveals progressive states of MuSC activation

Cell behavior analysis allows for the inference of cell state at the phenotypic level but does not provide direct insight into the molecular determinants of different cell states. To generate a portrait of the molecular state space of activating MuSCs, we employed scRNA-seq.

**Fig. 2.**
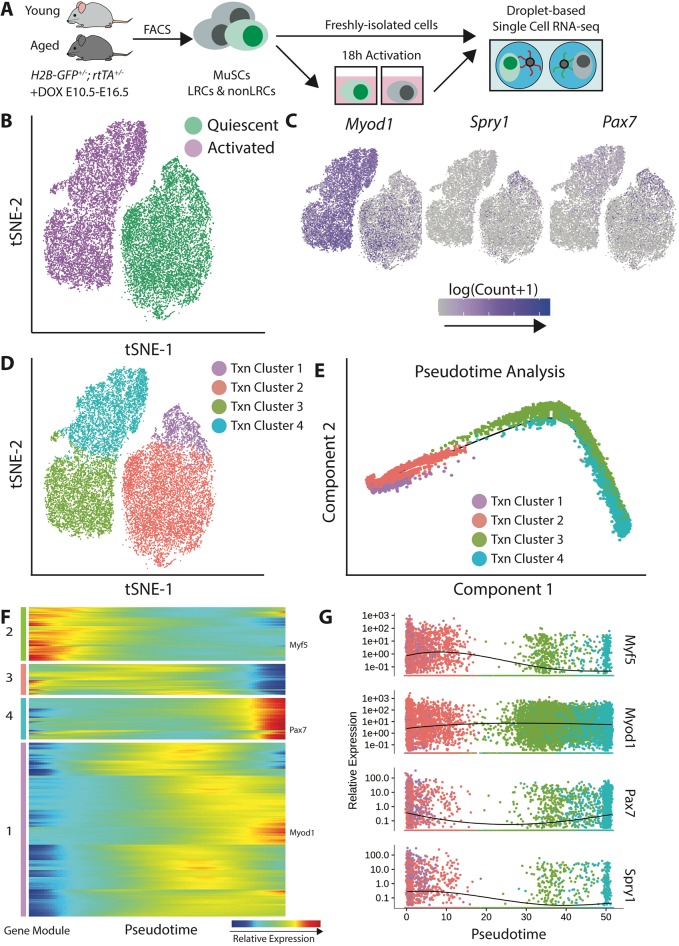
**Single cell RNA sequencing reveals heterogeneous transcriptional states during myogenic activation.** (A) Experimental schematic. Two animals were used for each age. (B) t-SNE visualization of quiescent and activated cells. 21,555 cells were recovered. (C) Overlay of myogenic regulatory genes on t-SNE plots to show that activated MyoD^+^ cells localize in a terminal state. (D) Definition of heterogeneous transcriptional states by unsupervised clustering. (E) Pseudotime analysis of MuSC activation, correctly recapitulating the sequence of ground truth time points. (F) Hierarchical clustering identifies four patterns of pseudotemporal gene expression during MuSC activation. (G) Visualizing myogenic regulatory gene expression across pseudotime reveals that Pax7 does not decrease monotonically.

We transcriptionally profiled MuSCs isolated from both young (3 months old, *n*=2) and aged (20 months old, *n*=2) *H2B-GFP^+/−^;rtTA^+/−^* mice. We previously showed that LRCs, which undergo fewer divisions during development, are more regenerative than nonLRCs, which undergo many divisions (Chakkalakal et al., 2012; Chakkalakal et al., 2014). To capture different states of MuSC activation within these populations, we profiled transcriptomes at two time points: (1) freshly isolated ‘quiescent’ MuSCs were processed for scRNA-seq immediately after FACS isolation, and (2) activated MuSCs were processed for scRNA-seq after 18 h in culture (Fig. 2A).

Recent studies have reported that the FACS-isolated cells used here express some markers of early MuSC activation, even immediately after isolation. Therefore, our ‘quiescent’ cell populations probably experience some early activation stimulus during isolation (Machado et al., 2017; Velthoven et al., 2017). Our *in vitro* activation assay might not fully capture *in vivo* stem cell activation dynamics. However, our *in vitro* assay allows for a homogeneous activation stimulus across all cells, unlike *in vivo* injury models and previous work demonstrating that *in vitro* MuSC activation recapitulates many aspects of *in vivo* activation (Blau et al., 2015; Cornelison and Wold, 1997; Cosgrove et al., 2014; Gilbert et al., 2010).

From 21,555 MuSC transcriptomes (Table S1), we found two discrete clusters of cells in transcriptional space. These discrete clusters correspond to the freshly isolated and 18 h activated cell time points, which we refer to as quiescent and activated cells, respectively (Fig. 2B). The discontinuity between the freshly isolated and activated time points suggests that there are intermediary activation states that are not captured by this method. Quantification of the proportion of variance explained by each experimental factor confirmed the qualitative observation that activation is the largest source of variation (Fig. S2A).

The expression of myogenic genes in the transcriptional space corroborates known MuSC biology, with the quiescence marker *Pax7* localizing largely to the quiescent cell population and activation marker *Myod1* in the activated cell population (Fig. 2C). However, single cell analysis of these markers in our large sample of MuSCs, revealed heterogeneity within both the quiescent and activated populations. Within the quiescent cell population, a subset of *Pax7^+^* cells occupied the opposite end of quiescent state space from a set of *Myod1^+^* cells. Likewise, a subset of cells in the activated population expressed the quiescence marker *Pax7^+^*.

Given this heterogeneity, we utilized unsupervised Louvain community detection to identify subpopulations within quiescent and activated cells (Blondel et al., 2008). Tuning the community resolution parameter yielded four transcriptional clusters. Two transcriptional clusters were identified within the quiescent and activated cell populations, respectively (Fig. 2D).

### Non-monotonic gene expression patterns are present in myogenic activation

We sought to determine how these transcriptional clusters are temporally ordered during activation using pseudotime analysis (Qiu et al., 2017). Pseudotiming revealed a distinct sequential ordering for these transcriptional clusters (Fig. 2E). Myogenic gene levels within each of the clusters corroborated the pseudotiming inference with known myogenic biology (Fig. S2B; Table S2). Transcriptional cluster 1 exhibited the highest levels of quiescence marker *Spry1*, and was appropriately selected as the root of the pseudotime axis. Likewise, transcriptional clusters 3 and 4, from the activated time point, expressed lower levels of the quiescence markers *Pax7*, *Spry1* and *Cd34*, and expressed higher levels of the activation marker *Myod1*. Across all clusters, there were no distinct cell-cycle states observed, consistent with reports of the first division occurring ≥24 hours after our 18 h time point (Gilbert et al., 2010; Rodgers et al., 2014) (Fig. S2D).

Pseudotime analysis placed transcriptional cluster 4 as the endpoint of the progression, despite the fact that it contains a subpopulation of *Pax7^+^* cells, whereas cluster 3 does not (Fig. 2C). This challenges the traditional dogma that *Pax7* levels decrease monotonically with MuSC activation and suggests a more complex temporal regulation of *Pax7* (Fig. 2G). Analysis of the mean expression in each cluster confirmed non-monotonic changes in *Pax7* with activation (Fig. S3A,B). Our data are consistent with previous reports of decreased *Pax7* with activation at the ensemble level (Cornelison and Wold, 1997; De Micheli et al., 2020; Dell'Orso et al., 2019), when we consider only the mean expression of *Pax7* at the quiescent and activated time points (Fig. S3C). When comparing *Pax7^+^* cells in transcriptional cluster 4 with *Pax7^−^* cells in the same cluster, we found that *Pax7^+^* cells showed significant upregulation of *Id1*, *Id2* and *Id3*, which inhibit differentiation (Jen et al., 1992), and of the cell-cycle gene *Ccnd1* (Fig. S3D). These results are consistent with the idea that *Pax7^+^* cells might be more proliferative (Zammit et al., 2006) and less committed than their *Pax7^−^* counterparts.

By contrast, the quiescence marker *Spry1* (Shea et al., 2010) displayed a monotonic decrease with activation and *Myod1* displayed a monotonic increase (Fig. 2G). Previous functional studies with *Pax7* overexpression constructs in MuSCs reported that *Pax7* promotes proliferation in certain contexts (Zammit et al., 2006), consistent with increased *Pax7* as cells enter into cycle later in the activation process. We observed that the commitment marker *Myog* was expressed in only 25 cells in our data set, as expected for this early time point. For this reason, we cannot estimate an accurate pseudotemporal curve (Fig. S4A).

Clustering genes into modules based on pseudotemporal expression patterns revealed that some genes exhibit monotonic increases or decreases with activation, whereas others display non-monotonic behavior (Fig. 2F; Table S3). For example, many genes in modules 1 and 3 displayed maximum expression at points in between the most quiescent and most activated states. Module 1 contained genes related to mRNA processing and splicing, as determined by gene ontology analysis. Module 3 contained genes related to cell cycle regulation and developmental processes (Fig. S4B). By contrast, most genes in module 2 decreased monotonically across pseudotime, and most genes in module 4 monotonically increased across pseudotime.

**Fig. 3.**
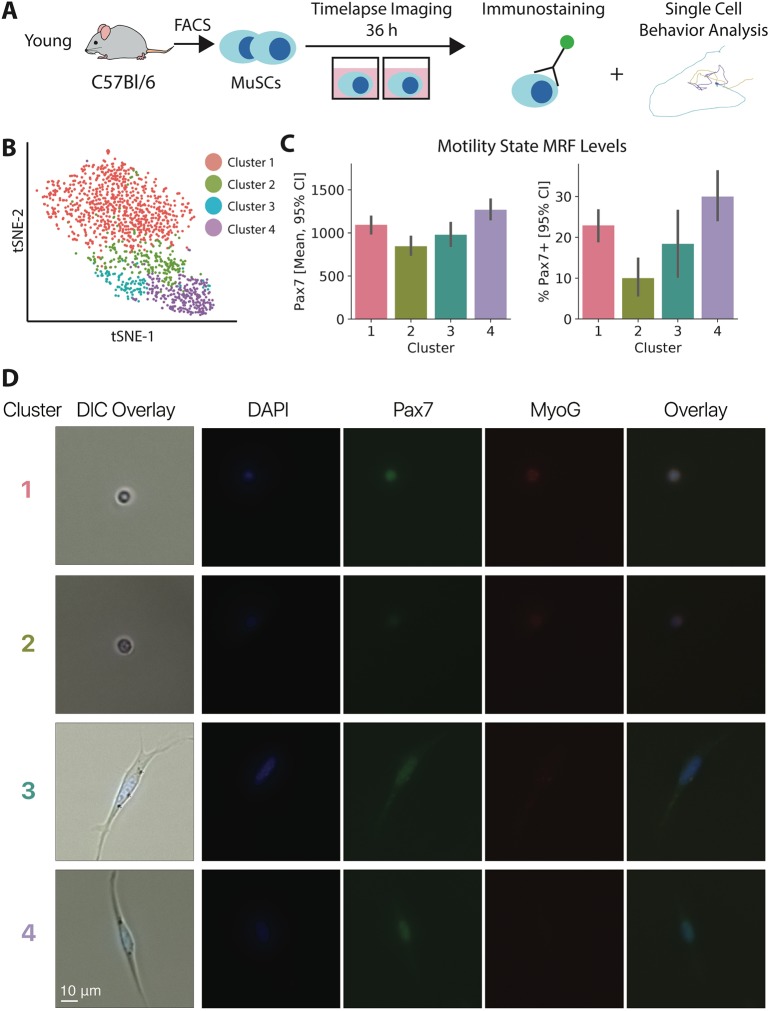
**Pax7 is non-monotonically regulated across MuSC cell behavior states during activation.** (A) Experimental design schematic. MuSCs were isolated, time-lapse imaged in culture for 36 h, and subsequently immunostained. Behavior traces and immunostaining results were matched for each cell by image registration. (B) t-SNE visualization of behavior clusters in motility state space, as defined by hierarchical clustering. Behavior state space was generated by analyzing 12 h of tracking data, from 24 h after isolation to 36 h (*n*=1003 cells, 1 animal). (C) Pax7 immunostaining intensity (cell median) and binary frequency within each cell behavior cluster. Both quantification schemes show a non-monotonic relationship between behavioral activation state and Pax7 intensity (95% confidence intervals from 1000 bootstrap samples). (D) Representative images of Pax7 and MyoG staining in MuSCs after time-lapse imaging for behavior analysis in each behavior cluster. Panels on the far left are the final DIC image from the time lapse, registered and overlaid with fluorescent immunostains. Remaining panels are fluorescent images prior to registration. Fluorescence images are equitably rescaled across cells within each channel for presentation.

As an orthogonal method to confirm cluster ordering and establish a link between the transcriptional clusters and behavioral clusters, we performed immunostaining following single cell behavior measurements. We found that the most motile and most activated cell behavior states are enriched for Pax7 protein (Fig. 3). Pax7^+^/MyoG^+^ cells were rare in this assay, as expected (Fig. S5). This analysis also indicated a non-monotonic regulation for Pax7 across cell behavior states, as inferred from the ordering of transcriptional clusters.

These results support the notion that transcriptional programs during myogenic activation exhibit a variety of temporal behaviors, including non-monotonic and nonlinear temporal regulation. Expression peaks and valleys in the non-monotonically-regulated gene modules provide evidence that there are intermediary transcriptional states of myogenic activation that are not simple interpolations of the initial and final transcriptional states.

We next identified markers of myogenic activation by differential expression between the quiescent and activated cell populations. Differential expression analysis revealed 3864 genes altered by activation. Gene ontology (GO) analysis of the differentially expressed marker genes suggests that these genes largely reflect biosynthetic and metabolic pathways, consistent with the notion that myogenic activation corresponds to a dramatic metabolic and geometric rearrangement of cellular state (Fig. S2C).

This interpretation was further reinforced by weighted gene correlation network analysis (WGCNA) (Langfelder and Horvath, 2008), which elucidated two gene modules during activation (Fig. S6A). The first ‘quiescence module’ was upregulated in quiescent cells and contains genes related to cell stress responses, transcriptional suppression, and negative regulation of cell proliferation, as indicated by GO analysis (Fig. S6B). By contrast, the ‘biosynthetic module’ was upregulated during activation and contains genes related to protein biosynthesis, transcriptional upregulation, ribosome biogenesis, and RNA maturation (Fig. S6C). Together, these results suggest that myogenic activation is heterogeneous among individual cells at multiple time points in the process, and that the activation process can be decomposed into a set of activation states reflecting cellular biosynthetic activity.

### Aged MuSC transcriptomes show modest differences across many transcripts

Aged MuSCs have significantly impaired regenerative capacity. We previously found that the conversion of highly regenerative LRCs to less regenerative nonLRCs is a factor in this regenerative decline (Chakkalakal et al., 2012, 2014). Do aged MuSC transcriptomes reflect these functional deficits? To answer this question, we randomly sampled populations of aged and young MuSCs with physiological ratios of LRCs:nonLRCs to mimic MuSC pools *in vivo* (Fig. S7G).

**Fig. 4.**
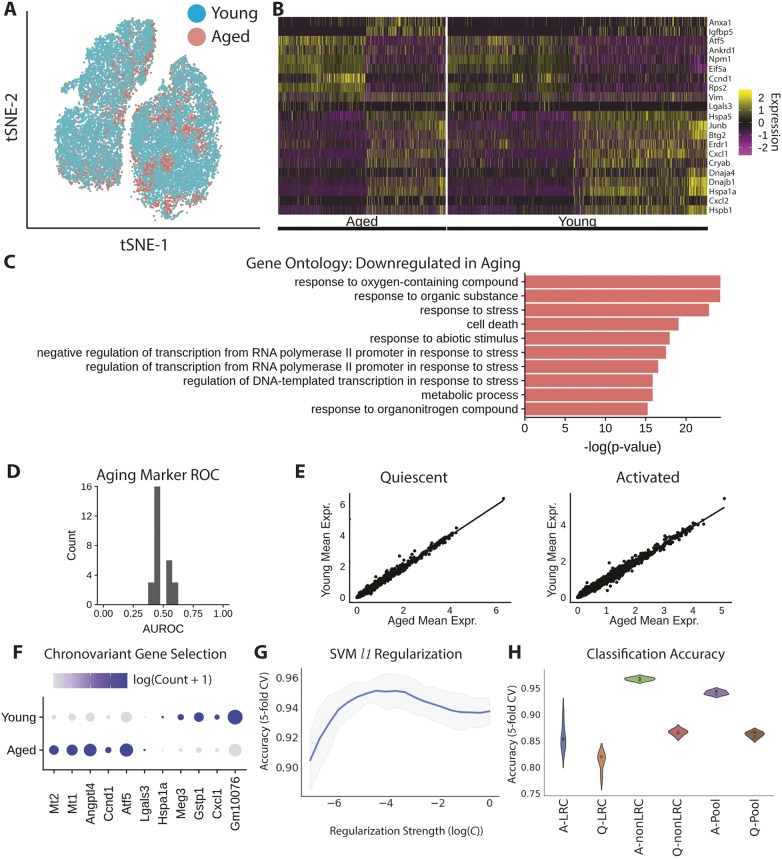
**Aged MuSCs display transcriptional changes across many genes.** (A) Aged and young cells labeled in a t-SNE projection of transcriptional space. (B) Heatmap of differentially expressed genes between aged and young MuSCs. Expression is mean centered and variance scaled for each gene (*q*<0.05, Wilcoxon Rank Sums, Bonferroni corrected). 7128 aged cells; 11,052 young cells in comparisons. (C) Gene ontology analysis for differentially expressed genes. (D) Gene-wise AUROC analysis demonstrates that no single gene is predictive of MuSC age state. (E) Correlation of aged and young transcriptomes. Each axis is the mean expression level in MuSCs of the indicated age and each point is a single gene. (F) Chrono-variant genes displayed on a dot plot. Darker colors indicate higher expression, larger dots indicate expression in a larger proportion of cells. (G) SVM classification accuracy versus regularization (L1) strength identifies a subset of genes for age discrimination. (H) Classification performance for aged versus young LRCs, nonLRCs and the total MuSC pool. A, activated; Q, quiescent.

Similar to our cell behavior analysis, we found that aged MuSCs do not segregate discretely in transcriptional space (Fig. 4A). Furthermore, we found high correlation between the gene expression profiles of young and aged MuSCs (Fig. 4E). There is no apparent preference in aged cells among the activated transcriptional clusters (χ^2^ test, *P*>0.9). This differs from the state preference of aged cells among the behavioral clusters we identified (Fig. 1D), suggesting that either the state preference arises after the 18 h time point captured by scRNA-seq, or that the state preference is less dramatic at the transcriptional level.

We used differential expression analysis to identify 174 differentially expressed genes between aged and young cells when comparing both quiescent and activated cells (Fig. 4B; Table S4). GO analysis of these genes indicated that they represent biosynthetic processes and stress responses, with protein translation processes upregulated with aging and stress responses downregulated (Fig. 4C). Among the differentially expressed genes, young cells displayed elevated levels of the stress response heat-shock proteins *Hspb1* and *Hspa5*, suggesting that aged MuSCs are less able to mount appropriate stress responses.

We also found differentially expressed genes with aging specifically within the quiescent and activated states. In quiescence, we found 200 differentially expressed genes between aged and young MuSCs (Fig. S7A; Table S5). GO analysis suggested that these genes are related to protein folding and cellular responses to environmental stresses. Both of these processes are downregulated in aged cells (Fig. S7B). During activation, we identified 275 differentially expressed genes, suggesting that activation accentuates age-related transcriptional differences (Fig. S7C; Table S6). GO analysis likewise identified that these genes are associated with catabolic processes (downregulated in aging), and stress responses (upregulated in aging) (Fig. S7D). Two of the top differentially expressed genes are associated with increases in longevity (Ladiges et al., 2009; Swindell, 2011).

### Gene expression variance is altered by aging

n addition to information about mean gene expression levels, single cell RNA-seq provides information about the variation in expression within cell populations. Recent work has suggested that aging might increase gene expression variance in immune cells (Martinez-Jimenez et al., 2017; Kolodziejczyk et al., 2015). We found that aged cell gene expression is more variable in the quiescent state (*P*<0.05, Wilcoxon Rank Sums). By contrast, young cell gene expression was more variable than activated aged cells at the activated time point (*P*<0.0001) (Fig. S8). This result suggests that the levels of gene expression variance are altered by aging in a context-dependent manner.

### Discrimination of aged and young MuSCs by machine learning

The field of aging biology seeks biomarkers of aging that can be used as an assay of the aged phenotype. No single gene serves as a good biomarker, as assessed using the area under the receiver operating characteristic (AUROC) (Fig. 4D). As an alternative approach, we used support vector machine (SVM) models with a sparsity penalty to classify aged and young MuSCs by incorporating information from multiple genes. We identified a set of candidate ‘chrono-variant’ genes that change with age, focusing on the case of activated MuSCs where differences are more pronounced (Fig. 4F,G).

We first applied this machine-learning approach to predict age within LRC and nonLRC subsets at each time point. Classifying activated LRC age, we identified a set of 54 genes that yield a prediction accuracy of ∼85% (holdout validation; Table S6) (Fig. 4H). For nonLRC classification, we identified 104 genes that yield a predictive accuracy of ∼96% (Fig. 4H; Table S6; Fig. S9). Of these genes, only 40 are common to both LRCs and nonLRCs, suggesting that transcriptional aging manifests differently in LRC and nonLRC populations. Classification of the activated young and aged MuSC pools (each sampled to model physiological LRC:nonLRC ratios) identified a set of 99 genes that provide ∼95% classification accuracy (Fig. 4H; Table S7). In each case (LRC, nonLRC and pooled), we found that classification of activated cells is more effective than classification of quiescent cells, further suggesting that activation reveals transcriptional aging phenotypes. This classification result represents the first effective assay to discriminate the age of individual muscle stem cells.

### Estimating the contribution of LRC to nonLRC conversion to transcriptional aging

The proportion of LRCs in the MuSC pool is ∼35% in young animals and decreases to ∼15% with age. To estimate the contribution of this cell state conversion to the overall changes we observe with age, we estimated the ‘magnitude’ of age-related changes using a classification-based density-ratio estimation method (Sugiyama et al., 2011). Populations of aged and young cells were simulated by random sampling with either physiologically observed LRC:nonLRC ratios or equal LRC:nonLRC ratios. The overall difference between young and aged cells did not change significantly across these conditions (Fig. S10A,B). The similarity in classification accuracy and divergence magnitude, in the face of changes to the LRC:nonLRC ratio, suggests that LRC-to-nonLRC conversion does not dramatically alter the overall ‘magnitude’ of age-related transcriptional change.

### Activation manifests transcriptional differences due to proliferative history

**Fig. 5.**
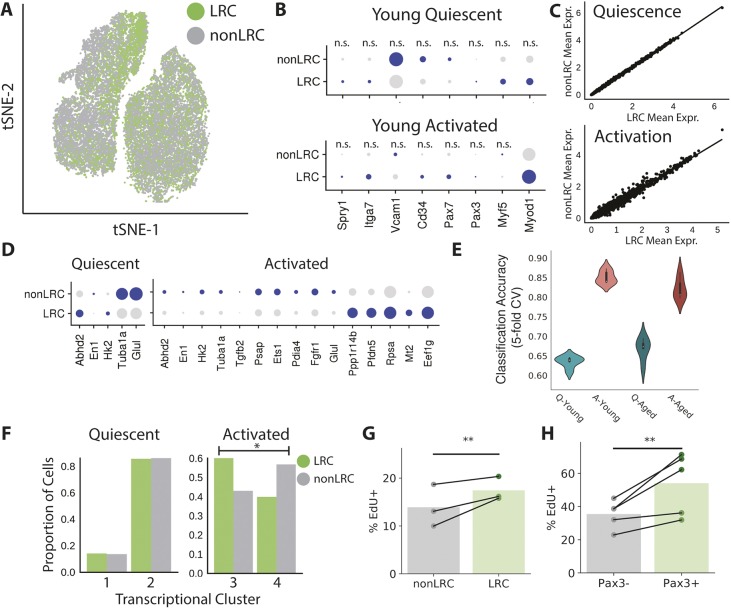
**Activation induces differential responses in LRCs and nonLRCs.** (A) LRCs and nonLRCs labeled in a t-SNE visualization of transcriptional space. (B) Expression of known myogenic regulatory genes in quiescent and activated LRCs and nonLRCs. Larger dots indicate a greater proportion of expressing cells, darker colors indicate higher expression. Only Myod1 differences in activation are significant (*q*<0.05, Wilcoxon Rank Sums, Bonferroni corrected). (C) Correlation between young LRC and nonLRC mean gene expression values in quiescence and activation. 6743 LRCs and 7184 nonLRCs in comparison. (D) Significant differentially expressed genes between LRCs and nonLRCs in quiescent and activated conditions (*q*<0.05, Wilcoxon Rank Sums, Bonferroni corrected). Few transcriptional differences are present in quiescence, but activation manifests differential expression across many genes. (E) LRC:nonLRC classification performance across activation states and ages. Classifiers more readily discriminate LRCs from nonLRCs in activation. A, activated; Q, quiescent. (F) Distribution of quiescent and activated LRCs/nonLRCs in transcriptional clusters indicates a heterogeneous activation response. (χ2 test, LRC × transcriptional cluster contingency table, *P*<0.05, *n*=6872 cells). (G) EdU incorporation by LRCs and nonLRCs during early activation in culture (*n*=3 mice; 10,279 LRCs; 13,464 nonLRCs; **P<0.001, Wald test). (H) EdU incorporation by Pax3^+^ and Pax3^−^ cells during early activation in culture (*n*=5 mice; 5169 Pax^+^ cells; 4130 Pax3^−^ cells; **P<0.001, Wald test).

LRCs are functionally distinct from nonLRCs, and this functional difference persists throughout life (Chakkalakal et al., 2012, 2014). Although we found that the majority of transcriptional differences between aged and young cells are independent of proliferative history, we also investigated whether LRCs and nonLRCs were transcriptionally distinct from one another. Similar to aged and young cells, LRCs and nonLRCs appear to share a transcriptional state space (Fig. 5A). We first investigated expression of known regulators and markers of the myogenic state in LRCs and nonLRCs. We found that the majority of myogenic markers are not differentially expressed between LRCs and nonLRCs in this assay (Fig. 5B). Likewise, the mean gene expression levels between the two states have a near perfect correlation in quiescent cells (*r*=0.99; Fig. 5C).

However, *Myod1* is upregulated in LRCs after activation but not in quiescence (Fig. 5B). Regression analysis of mean gene expression levels between the LRC and nonLRC states revealed a near-perfect correlation in quiescent cells, but in activation the mean gene expression values were less correlated (*P*<0.001, Fisher's *r* to *z* transformation; Fig. 5C). Therefore, activation induces different transcriptional responses in LRCs and nonLRCs.

Differential expression analysis revealed that 97 genes were differentially expressed between the LRC and nonLRC pools (Fig. S11; Table S8). Considering quiescent and activated cells separately, there were only 14 differentially expressed genes in quiescent cells but 195 differentially expressed genes in activated cells (Fig. 5D; Tables S9,S10).

The differentially expressed genes include known stress response genes, such as heat shock proteins and inflammation signatures, largely enriched within the nonLRC population. Resiliency and biosynthetic genes were also differentially expressed, with known longevity factor (Swindell, 2011) *Mt2* and protein translation components *Pfdn5* and *Eef1g* enriched in LRCs. GO terms for nucleoside triphosphate metabolic processes were enriched in genes upregulated in young LRCs, whereas terms for cell death and apoptotic processes were enriched in young nonLRC upregulated genes (Fig. S10D,E). We performed similar differential expression analyses for aged LRCs and nonLRCs (Tables S11-S13). These expression differences suggest that LRCs might possess a biosynthetic advantage over nonLRCs.

The distribution of LRCs and nonLRCs among clusters of activation confirmed a differential activation response. In the quiescent state, LRCs and nonLRCs did not display significant differences in distribution between the two quiescent transcriptional clusters. However, after activation, LRCs were significantly enriched in the most activated transcriptional cluster 4 (χ^2^ test, *P*<0.05). Approximately 60% of activated LRCs were in the most activated transcriptional cluster 4, compared with only 40% of the activated nonLRCs (Fig. 5F).

The higher proportion of LRCs in the most-activated region of transcriptional space suggests that LRCs might activate more quickly than nonLRCs. The enrichment of LRCs in transcriptional cluster 4 may also underlie the non-monotonic behavior of *Pax7* across pseudotime (Fig. 2G). LRCs express higher levels of *Pax7* (Chakkalakal et al., 2012), such that rapid activation of LRCs might give rise to a population structure that present non-monotonic changes in *Pax7* when averaging across all cells at a given activation state.

This result is surprising, as LRCs are believed to be ‘reserve’ stem cells, whereas nonLRCs are presumed to be precocious in their response based on differentiation assays (Chakkalakal et al., 2012, 2014). To confirm this observation with an orthogonal assay, we measured ethynyl-2′-deoxyuridine (EdU) incorporation in young LRCs and nonLRCs during activation in culture. LRCs incorporated EdU at a higher rate (21%) than nonLRCs (17%) in 20,000 cells across three animals (Fig. 5G; *P*<0.001, Wald test, logistic regression). Consistent with earlier cell cycle entry, we observed that *Ccnd1* was one of the most upregulated genes in LRCs relative to nonLRCs (Fig. S11).

We recently identified a population of stress-resistant, highly regenerative *Pax3^+^* MuSCs that overlap with LRCs (Scaramozza et al., 2019). To determine if rapid activation is also observed in this highly regenerative population, we analyzed EdU incorporation in *Pax3^+^* versus *Pax3^−^* MuSCs during early activation (Relaix et al., 2005). Similar to our experiment in LRCs/nonLRCs, we found that highly regenerative *Pax3^+^* MuSCs also activate more rapidly than *Pax3^−^* counterparts (Fig. 5H; *P*<0.001, Wald test, logistic regression), consistent with a report by De Morree et al. (2019). Collectively, these results suggest that LRCs activate more rapidly than nonLRCs, based on both the progression through transcriptional space and the timing of cell cycle entry.

Similar to our classification of MuSCs from different ages, we trained classifiers to discriminate LRCs and nonLRCs. We trained a separate model to classify LRC/nonLRCs at each time point and each age. Classification of quiescent cells performs poorly at both ages (∼65% accuracy), whereas classification of activated cells is more effective – ∼85% accuracy for young cells and ∼80% for aged cells (Fig. 5E; Fig. S10C). As in our age classification experiments, this result suggests that activation reveals differences between cell populations that are masked in quiescence. Our regularized models identified 102 genes that optimize LRC/nonLRC classification of young activated cells, and 72 genes that optimize classification of aged activated cells (Table S14).

### Transcriptional kinetics are aberrant in aged MuSCs

The lack of unique aged transcriptional states and modest differential expression results between aged and young cells are surprising in light of the dramatic differences in functional potential between aged and young cell populations (Chakkalakal et al., 2012; Cosgrove et al., 2014). These results suggested to us that the rate at which aged and young cells activate might be an additional source of variation that contributes to their functional differences. To quantify rates of phenotypic change between the MuSC transcriptional states during activation, we utilized the recently developed RNA velocity method (La Manno et al., 2018). This method estimates a ‘velocity’ of transcription, or rate of change in the transcript level, by estimating mRNA decay rates and transcription rates using ratios of spliced to unspliced reads.

**Fig. 6.**
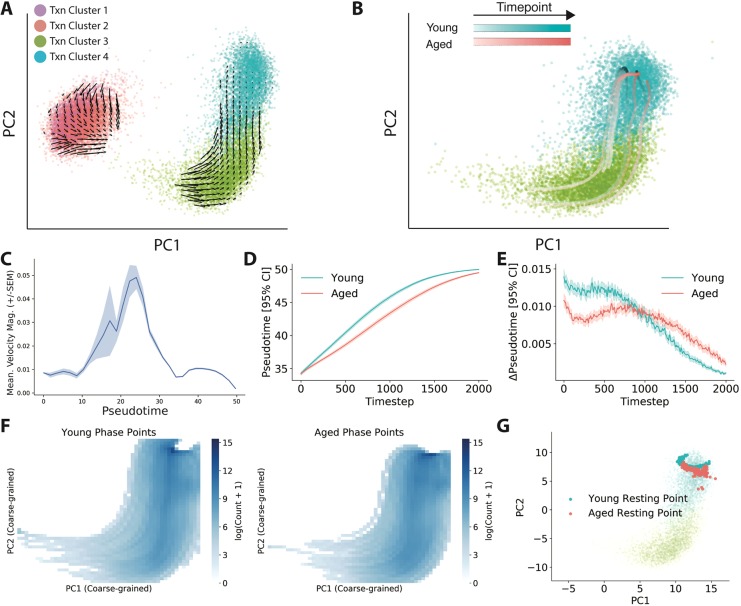
**Aged MuSCs transition aberrantly through transcriptional space.** (A) MuSC transcriptional space overlaid with arrows representing the direction and magnitude of RNA velocity at each state location (20,827 cells). Colors indicate transcriptional clusters. (B) Representative phase point simulations in aged and young RNA velocity fields, overlaid on the activated MuSC cells in a PCA embedding. (C) State transition rates as measured by RNA velocity magnitude across pseudotime using a rolling mean. (D) Predicted pseudotime progression for phase point simulations in either aged (red) or young (blue) velocity fields. Young phase points progress more rapidly than aged cells. Curves cross when young and aged phase points have both reached a steady-state at the end of our observed pseudotime trajectory. *n*=1000 phase points simulated for each age. (E) Change in pseudotime for phase point simulations at each time step. (F) Heatmap representing the mean density of phase points at each point in principal components space across the entire simulation. Young and aged phase simulations show qualitatively similar trajectories through state space. (G) Terminal locations of young and aged phase point simulations in principal components space, overlaid on cell locations. Both young and aged simulations show similar final resting positions.

RNA velocity estimation showed that each state of MuSC transcription gave rise to a neighboring state in the sequence inferred by pseudotiming (Fig. 6A). As an internal validation check, we found that RNA velocity indicated that quiescent cells were moving toward activated cells in transcriptional space.

The magnitude of mean RNA velocity represents the rate of collective phenotypic change at the transcriptional level for a given group of cells. This approach provides an inferred measurement of state transition rates in transcriptional space, similar to the measurement of state transition rates we make by direct observation in cell behavior space. Quantification of the magnitude of RNA velocity across pseudotime in MuSCs revealed that RNA velocity follows a concave curve (Fig. 6C).

Concave transition rates are consistent with a model in which the peak transition rate represents a ‘switch’ between two states, corroborating our earlier observations made by cell behavior analysis (Kimmel et al., 2018). We note that the switch-like process suggested by these results might be unique to our *in vitro* culture setting, and does not necessarily reflect *in vivo* activation kinetics. Consistency in state transition measurements between RNA velocity and cell behavior phenotyping suggests that cell behavior state transitions reflect the underlying transcription state kinetics.

Do aged and young MuSCs move through transcriptional state space differently? To answer this question, we employed a method to model cellular progression through transcriptional space using phase point simulations. Phase point analysis is a dynamical systems method to investigate the properties of a vector field in which a simulation is performed to determine how a particle might flow along a vector field, as if it were a floating leaf carried by currents in a river (Strogatz, 2015). Here, we simulated a set of phase points that begin in the more primitive regions of our ‘activated’ 18 h time point and evolved them over time using velocities inferred from either young or aged cells (Fig. 6B). Given these simulated trajectories through transcriptional space, we investigated whether notable differences are present in phase points simulated using young velocities (‘young phase points’) relative to those simulated using aged velocities (‘aged phase points’).

To assess progress through cell activation, we trained a *k*-nearest neighbors regression model (kNN-R) to map transcriptome principal component analysis (PCA) embeddings to pseudotime coordinates as determined using Monocle 2 (Figs S5; S12A,B). For each time step in a phase point simulation, we predicted a pseudotime coordinate of the point using this model. Comparing these inferred coordinates, young phase points progressed more rapidly through the activation process than aged phase points (Fig. 6D). After many time steps, both young and aged phase points reached similar inferred pseudotime locations as they neared the edge of our observed pseudotime trajectory (Fig. 6D).

The computation of numerical derivatives for pseudotime coordinates, Δpseudotime, showed that young phase points appeared to progress more rapidly from the earliest time steps (Fig. 6E). This result suggests that aged cells might progress more slowly than young cells through the activation process in a similar manner to the phase point simulations. Results were robust to initialization conditions and the introduction of noise (Fig. S12C).

Phase point simulations provide additional information about the location of ‘attractors’ in transcriptional space. Attractors are locations in a state space where phase points tend to converge and come to rest. Visualization of the density of phase points in transcriptional space as the number of phase points to pass through a region, showed there were qualitatively few differences in the shape of trajectories between young and aged cells (Fig. 6F; Movie 5). Focusing on the locations where phase points come to rest, there are likewise modest differences in the specific shapes of attractor states, but overall similar attractor positions between young and aged phase point simulations (Fig. 6G).

Collectively, results of these phase point simulations suggest that the set of intermediate transcriptional states a MuSC visits in the course of activation is largely similar between young and aged cells. However, aged cells appear to activate at a slower rate than young counterparts. Each of these points supports the different rates model of aging pathology outlined above.

### Lineage regression occurs during myogenic activation in a subset of MuSCs

The discovery of reserve cells generated during myogenic commitment more than 20 years ago first presented the idea that MuSCs might revert to earlier stages in the lineage progression under some conditions (Yoshida et al., 1998). It is currently unclear how frequently MuSCs transition ‘backwards’ in the myogenic activation program. We assessed the frequency of MuSCs transitioning backward in the lineage progression by quantifying a ‘change in pseudotime’ (Δpseudotime) for each cell in our young MuSC single cell RNA-seq data set. Δpseudotime was estimated using the kNN-R model referenced above. Pseudotime values were predicted for the ‘future’ transcriptomes inferred by RNA velocity, and the difference between the predicted pseuodotime and observed pseudotime values was taken as the Δpseudotime. We defined a cell as ‘regressing’ in pseudotime if Δpseudotime was more than half a standard deviation below 0.

**Fig. 7.**
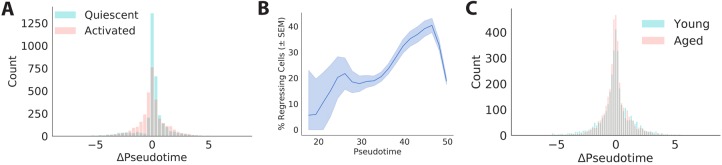
**MuSCs exhibit lineage regression during activation.** (A) Distribution of changes in pseudotime (Δpseudotime) inferred from RNA velocity estimates in young quiescent and activated cells. Activated cells regress more frequently. (B) Proportion of cells moving backward in pseudotime as a function of position along the pseudotime curve for cells in the later experimental time point (18 h *in vitro*). Proportions were computed across coarse-grained regions of pseudotime and Gaussian smoothed for presentation. Data are mean±s.e.m. (C) Distribution of changes in pseudotime for young and aged MuSCs across conditions.

This analysis reveals that ∼16% of young MuSCs are regressing in pseudotime during the period of myogenic activation we measure (Fig. 7A). Regression is more frequent in activated (∼20%) than quiescent (∼15%) MuSCs. Quantification of the frequency of ‘lineage regression’ across pseudotime for cells from our later time point (18 h *in vitro*) revealed that cells regress more frequently in the later stages of activation we observed (Fig. 7B). RNA velocity is a measure of instantaneous change in the cell state, such that these results do not necessarily suggest a subset of cells that permanently fails to activate. Rather, these results suggest that myogenic activation is a two-way process even under growth-promoting conditions, perhaps resembling a biased random walk through transcriptional space.

This regression behavior appears to be robust to age-related changes (Fig. 7C; Fig. S13A). When comparing LRCs with nonLRCs, LRCs regress less frequently than nonLRCs in young and aged animals (Fig. S13B,C; χ^2^ test, *P*<0.0001). This decreased frequency of lineage regression may contribute to the more rapid activation of LRCs we observe based on transcriptional profiles (Fig. 5) and EdU incorporation (Fig. 5G). To determine which genes underlie differences in motion through the activation trajectory, we computed the difference in velocity between forward and backward moving cells. This analysis reveals that forward moving cells have a higher velocity for protein translation and biogenesis genes, whereas backward moving cells have a higher velocity for mitochondrial respiration genes (Fig. S13D, Gene Ontology enrichments; Table S15), consistent with the upregulation of protein synthesis and involvement of oxidative phosphorylation in myogenic activation (Ryall et al., 2015).

## DISCUSSION

Dynamic changes in stem cell phenotypes are essential for both development and regeneration. However, because of the difficulty of measuring single cells over time, quantitative understanding of these processes remains elusive (Almada and Wagers, 2016). Changes in these cell state transition rates might explain some portion of the decreased regenerative potential observed in aged stem cells, as in the muscle stem cell system. Some dynamic information can be inferred from static ensemble measurements, a class that includes destructive molecular assays. This type of inference is unable to assess whether the progression of cells through a set of states is uniform or heterogeneous, whether intermediary states are bistable or transient, or whether a given cellular feature influences velocity (Weinreb et al., 2018). Answering these questions requires some additional dynamical information, i.e. individual cells must be measured at multiple time points.

Here, we used time-lapse imaging and RNA velocity inference from single cell RNA-seq to provide paired measurements of this nature to investigate muscle stem cell activation *in vitro*. We surprisingly found minor transcriptional differences between aged and young MuSCs at a given point in the activation process, consistent with previous reports (Cosgrove et al., 2014; Keyes et al., 2016; Sousa-Victor et al., 2014). Although transcriptional differences with age have been observed previously, it remained unknown if these differences were a large enough source of variation to alter the trajectory of myogenic activation. Our single cell RNA-seq data allows us to observe that age-related changes are much smaller than changes between states of activation, such that the trajectory of activation is preserved with aging.

By measuring stem cell state transitions directly, we observed that aged MuSCs have dampened state transition rates. By behavioral analysis, aged MuSCs displayed a preference for less-motile, less-activated states and decreased rates of transition into more active states. Similarly, phase point analysis of RNA velocity vectors suggests that aged cells transition more slowly through transcriptional states during the earliest phases of activation than young counterparts. These data support a conceptual model in which aging MuSCs exhibit ‘different rates’ of activation, even if they follow the same trajectory. These differences in activation rate suggest that some transcriptional differences between young and aged MuSCs observed at the ensemble level may be the result of differences in the distribution of cells across the activation trajectory.

Our data also reveal heterogeneity in the stem cell activation process. Ensemble read-outs during MuSC activation have demonstrated that MuSCs occupy different transcription factor states, even at a single time point (Cornelison and Wold, 1997). However, these measurements could not explain where in the activation process heterogeneity arose. Here, we find that MuSCs progress through the activation process stochastically, with a nontrivial proportion of the population moving ‘backwards’ through the activation process. This suggests that the heterogeneity of MuSC positions along the activation trajectory arises as an accumulation of differences in the rate of cell state transitions.

These differences appear to be both stochastic and associated with distinctive features between MuSC subpopulations, such as proliferative history. Although the macroscopic processes of muscle development and regeneration proceed without these apparent reversals, these observations indicate that phenotypic change at the cellular level might involve considerably more noise. This is reminiscent of the qualitative differences between the physical motion of macroscopic objects, like a ball rolling down a hill, and microscopic motion, where noise can dominate the movement of small molecules, which often reverse direction completely.

The *in vitro* model we employ allows us to assess cell-intrinsic differences between young and aged MuSCs with a homogeneous activation stimulus, but may not fully recapitulate the *in vivo* biology. For instance, although we do not observe bistable transcriptional states between the most-quiescent and most-activated cells in our experiments, stable intermediary activation states might exist *in vivo* (Rodgers et al., 2014). Future work may explore stem cell activation *in vivo* to determine if cell-extrinsic changes with age exacerbate or dampen the differences in activation we observe between young and aged MuSCs. *In vivo* imaging technologies for MuSCs are nascent (Webster et al., 2016), but technological advances may allow for similar quantitative cell behavior analysis *in vivo* in the future. Future work may also explore the molecular determinants of stem cell activation rates to enable therapeutic rescue of MuSC activation dynamics in the aged context.

## MATERIALS AND METHODS

### Animals

Animals were handled according to the University of California San Francisco (UCSF) Institutional Use and Care of Animals Committee (IUCAC) guidelines. All experimental mice were male *Mus musculus* of the C57Bl/6J background. All mice were born at UCSF and aged in-house. All mice for single cell sequencing experiments harbored *H2B-GFP^+/−^; rtTA^+/−^* alleles and were developmentally labeled for proliferative history by the administration of doxycycline between embryonic day (E) 10.5 and E16.5 (Chakkalakal et al., 2014; Foudi et al., 2009). Aged mice for RNA-seq sequencing experiments were 20 months old and young mice were 3 months old. For one behavior experiment (Fig. 1), one young (3 months old) and one aged (20 months old) *H2B-GFP^+/−^; rtTA^+/−^* mouse with LRCs labeled developmentally as above, were used as sources of young and aged MuSCs, respectively. We cultured LRCs/nonLRCs in separate wells after FACS sorting and reconstituted observed young (35% LRC) and aged (15% LRC) proportions by random sampling *in silico* for downstream analysis. For the second behavior experiment (Fig. S1), we isolated cells from wild-type young (5 months old) and aged (23 months old) mice. Adult (3-4 months old) *Pax3-GFP/^+^* reporter mice were used for MuSC activation experiments (Relaix et al., 2005).

### Cell isolation and culture

Muscle stem cells were isolated by FACS using a triple-negative CD31^−^/CD45^−^/Sca1^−^ and double-positive VCAM^+^/*α*7-integrin^+^ strategy as described previously (Chakkalakal et al., 2012). Antibody clones, sources and catalog numbers are described in supplementary Materials and Methods. Cells were seeded in sarcoma-derived extracellular matrix (ECM)-coated 96-well plates in rich growth medium [F10 (Gibco), 20% fetal bovine serum (FBS) (Gibco), (5 ng/ml) FGF2 (R&D Systems)] for behavior analysis. For single cell sequencing experiments, cells were seeded in sarcoma-derived ECM-coated 6-well plates and allowed to activate in plating media [Dulbecco's modified eagle media (DMEM), 10% horse serum] for 18 h prior to library preparation.

Cells for behavior analysis were seeded at 850 cells/well on sarcoma-derived ECM (Sigma-Aldrich) in 96-well plates. Cells were maintained in rich growth media [F10 (Gibco), 20% FBS (Gibco), (5 ng/ml) FGF2 (R&D Systems)]. For single cell sequencing experiments in which cells were activated, cells were seeded in sarcoma-derived ECM-coated 6-well plates and allowed to activate in plating media (DMEM, 10% horse serum) for 18 h prior to library preparation.

### Time-lapse imaging and cell behavior analysis

MuSCs were imaged in 96-well plates on an incubated microscopy platform (Okolab) for 48 h. Images were collected with DIC contrast every 6.5 min to track cell movement (supplementary Materials and Methods). Cell behavior was analyzed using Heteromotility, as described previously (Kimmel et al., 2018; available at github.com/cellgeometry/heteromotility) (supplementary Materials and Methods).

### Paired immunohistochemistry and time-lapse imaging

For paired behavior-immunocytochemistry experiments, cells were fixed in 4% paraformaldehyde (PFA) for 10 min immediately following the imaging timecourse and stained with using a standard protocol (supplementary Materials and Methods). After staining, cells were returned to the same time-lapse microscopy system used for behavioral imaging, and fluorescent images were captured. We performed segmentation of the final brightfield image from the behavioral time course, and the nuclear channel of the fluorescence-stained image, then performed image registration using nearest neighbors to match immunofluorescence signals to cell behavior tracks. Stain intensity was quantified as the median intensity of a fluorescent signal within the nucleus of each cell.

### EdU staining

For LRC/nonLRC experiments, muscle stem cells were isolated as described above from three *H2B-GFP^+/−^; rtTA^+/−^* mice (4 months old, labeled developmentally as above) and cultured in plating media (DMEM, 10% horse serum) for 50 h. EdU (Carbosynth) was pulsed into the media at 12 h and 2 h (10 µM final concentration) prior to fixation with 4% PFA for 15 min. Staining followed the Click-iT EdU Alexa Fluor 647 kit protocol (Thermo Fisher Scientific). For *Pax3*^+^/*Pax3*^−^ experiments, *Pax3*^+^ and *Pax3*^−^ MuSCs from forelimbs and pectoralis muscles were collected by FACs from *Pax3-GFP/^+^* adult mice, plated on ECM-coated chamber-slides and cultured in high-serum conditions (20% FBS, F10) for 42 h. EdU (10 µM) was added during the final 12 h. EdU was detected by fluorescence microscopy. We tested the significance of EdU incorporation using logistic regression models and the Wald test (supplementary Materials and Methods).

### Single cell RNA-sequencing

LRC and nonLRC MuScs were isolated by FACS based on GFP intensity, as described previously (Chakkalakal et al., 2014). Half of the collected cells were immediately transferred to a 10x Genomics Chromium system for library preparation using the 10x 3′ Single Cell v. 2 chemistry. The remaining cells were activated by *in vitro* cell culture (as described in the ‘Cell isolation and culture’ section above), then dissociated using Cell Dissociation Buffer, (Gibco, 13151014) stained with propidium iodide (PI), and sorted by FACS to remove dead cells. Live activated cells were transferred to the 10x Chromium system for identical library preparation. Libraries were pooled and sequenced using an Illumina NovaSeq platform.

### Single cell transcriptome analysis

Raw sequencing data were demultiplexed using Illumina bcl2fastq. Demultiplexed sequencing reads were aligned to the mouse transcriptome using the STAR aligner (Dobin et al., 2013). Individual unique molecular identifiers (UMIs) were detected and assigned to corresponding cell barcodes using 10x Genomics cellranger, samplewise. Droplets containing cells were identified and individual libraries were aggregated using cellranger.

A genes×cells count matrix was generated from the aggregated libraries using cellranger. Suspected dead cells were removed when a high proportion of total UMIs in the cell mapped to mitochondrial genes (Ilicic et al., 2016) (>10% mitochondrial reads). Putative doublets were removed as outliers on a histogram of UMIs/cell and genes/cell (Brennecke et al., 2013) (>5000 genes/cell). Prior to normalization, the annotated transcripts *Gm42418* and *AY036118* were removed from the count matrix. These transcripts overlap an unannotated *Rn45s* rRNA locus, and may include counts from rRNA molecules that were amplified during library preparation despite polyA-selection.

Raw counts were log normalized using Seurat (Satija et al., 2015). Variable genes were identified using ‘FindVariableGenes’ in Seurat and PCA was performed on the variable gene set. *t*-SNE was performed on the principal components with perplexity *P*=30. Community detection was performed with the Louvain algorithm (Blondel et al., 2008). Small clusters of contaminating cells without myogenic marker genes were removed from subsequent analysis.

### Contribution of factors to transcriptional variation

The proportion of variation explained by each experimental factor in our multi-factor experiment was estimated with linear models as described previously (Robinson et al., 2015) (supplementary Materials and Methods). We scored cell cycle states using the approach in Seurat, introduced in Tirosh et al. (2016).

### Overdispersion analysis

Overdispersion scores were computed using the difference from the median (DM) method (Kolodziejczyk et al., 2015). We eliminated all genes with a mean expression lower than µ=0.1, as technical noise for genes with very low mean expression is known to be high (Kolodziejczyk et al., 2015) (supplementary Materials and Methods).

### Estimation of LRC to nonLRC contribution to transcriptional change

We estimated the ‘magnitude’ of transcriptional change with aging between a set of young and aged transcriptomes by training probabilistic classifiers to estimate the density ratio between distributions of young and aged transcriptomes (Rosca et al., 2017 preprint; Sugiyama et al., 2011). We used estimated density ratios to compute a Kullback–Leibler divergence between two samples (supplementary Materials and Methods).

### Pseudotiming

Pseudotime analysis was performed using the Monocle 2 package (Qiu et al., 2017). Genes for pseudotemporal ordering were determined by differential expression analysis between the transcriptional clusters, as described in the following section. Pseudotiming was performed on all 20,000+ cells that passed quality control simultaneously in the same transcriptional state space utilizing the DDRTree method with two components. We further clustered genes into gene modules based on their pseudotemporal behavior using the Monocle 2 function ‘plot_pseudotime_heatmap’. For this clustering analysis, we focused on genes that were significantly differentially expressed across pseudotime (*q*<0.001 in *n*≥3000 cells), as determined using negative binomial regression models fitted with the Monocle 2 command ‘differentialGeneTest’ with a full model formula of ‘sm.ns(Pseudotime)’ and a reduced model formula of ‘1’.

### Differential expression analysis

Differentially expressed genes between two populations, *A* and *B*, were determined based on a significant difference detected by the Wilcoxon Rank Sum Test. Genes with a fold change less than 0.15, or genes expressed in fewer than 10% of cells, were filtered out from the analysis to refine the gene set. Multiple hypothesis test correction was performed with the Bonferroni procedure. Receiver operating characteristic (ROC) scores were generated for each gene *g* based on a simple binary threshold classifier trained on only the expression of gene *g*.

### Gene ontology and pathway analysis

Gene ontology enrichment analysis was performed using g:Profiler and the gProfileR package, considering enriched gene ontology terms for biological processes and KEGG pathways.

### Support vector machine classification

Support vector machine (SVM) classification models to discriminate cell age and LRC status were trained using scikit-learn implementations (Pedregosa et al., 2011) (supplementary Materials and Methods). Accuracies were assessed using a held-out test set after optimizing regularization strength (supplementary Materials and Methods).

### RNA velocity analysis and dynamical simulations

RNA velocity was inferred using velocyto (La Manno et al., 2018) with default parameters (supplementary Materials and Methods). The magnitude of RNA velocity relative to pseudotime was quantified by binning cells along the pseudotime axis and computing the magnitude of the mean RNA velocity for each bin.

We trained kNN-R to predict pseudotime coordinates from PCA coordinates (*r*^2^>0.96 performance). To determine differences between aged and young velocity fields, phase point simulations were performed with numerical methods. A set of 1000 initial positions in the 2D PCA embedding for both young and aged cells was sampled from observed cellular positions in the primitive region of the activated time point. Phase point positions were evolved for *t*=5000 time steps, using a *k*-nearest neighbors approach to compute a velocity estimate and update point position at each iteration (supplementary Materials and Methods). For simulations in young and aged velocity fields, only young or aged cells, respectively, were considered at this step.

### Change in pseudotime analysis

The ‘change in pseudotime’ (Δpseudotime) was estimated for each cell using the kNN-R (supplementary Materials and Methods). Future transcriptional states *x*_t+1_ were inferred by RNA velocity as above, and the pseudotimes for these states were predicted using the kNN-R model. ΔPseudotime is defined as the difference between the inferred future and measured present pseudotime for each cell:
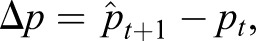
where 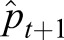 is the inferred pseudotime using RNA velocity and the kNN-R model and *p*_*t*_ is the observed pseudotime at the experimental time point. Cells were defined to be undergoing ‘lineage regression’ if they displayed a Δpseudotime<σ, where σ is the standard deviation of the Δpseudotime distribution.

### Statistical considerations

We defined sample size as the number of cells in single cell RNA-seq, single cell behavior experiments, and single cell imaging experiments. We also report the number of independent animals from which cells were isolated. Investigators were not blinded during the study. Appropriate statistical tests were employed for each data modality.

## Supplementary Material

Supplementary information

Reviewer comments
