# Supplementary material for: Aging induces aberrant state transition kinetics in murine muscle stem cells
Source: Development. 2020 May 5;147(9):dev183855. doi: 10.1242/dev.183855 (PMC7225128; doi:10.1242/dev.183855)
Supplement: Supplementary information [file develop-147-183855-s1.pdf]

1 Supplemental Files

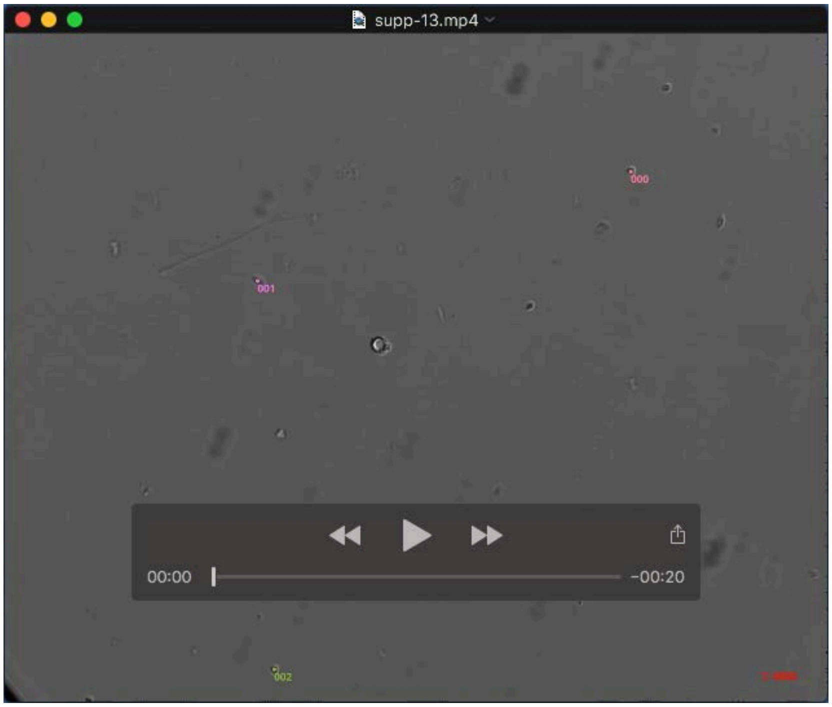

Movie 1 Muscle stem cells from a young animal, tracked for 25 hours during early activation. Numeric labels follow single cells that are confidently tracked and used for analysis.

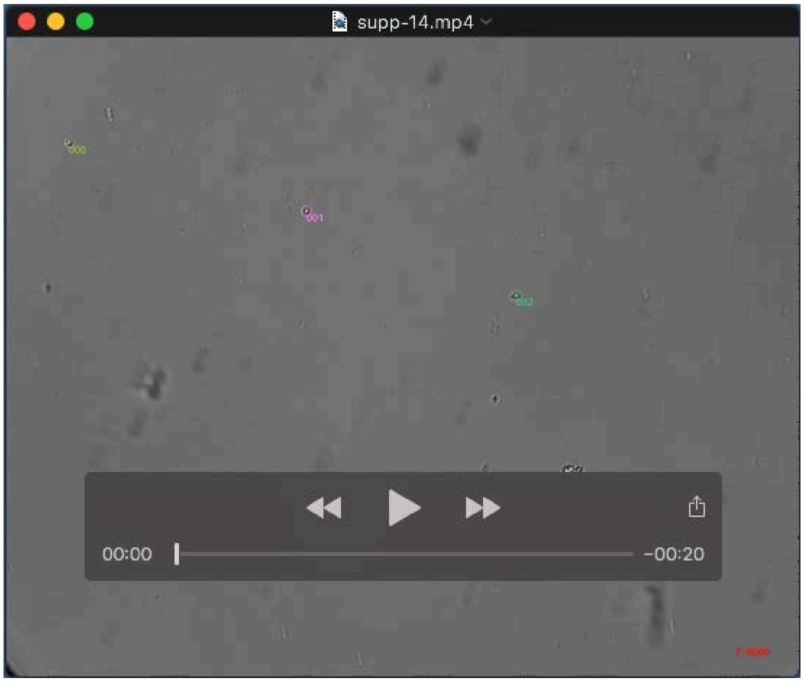

Movie 2 Muscle stem cells from a young animal, tracked for 25 hours during early activation. Numeric labels follow single cells that are confidently tracked and used for analysis.

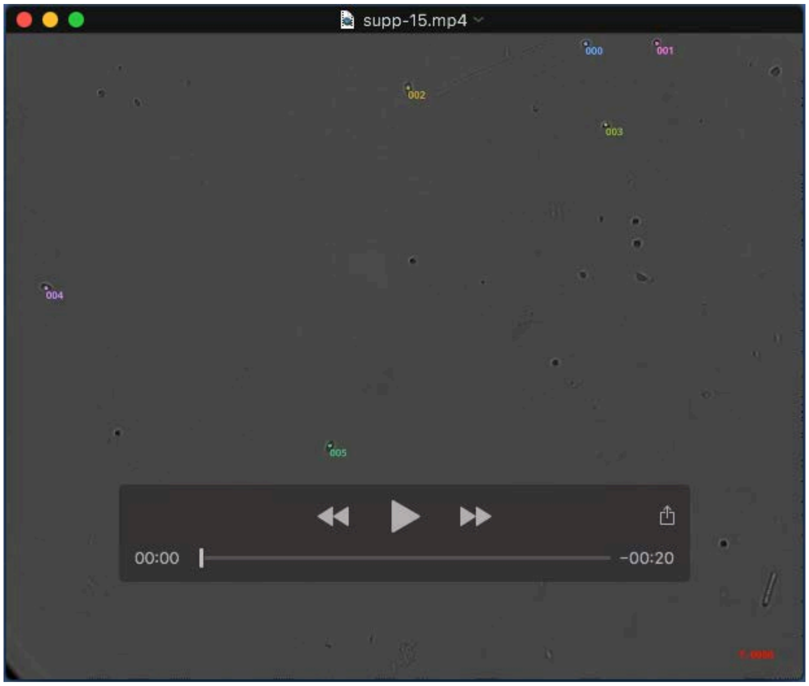

Movie 3 Muscle stem cells from an aged animal, tracked for 25 hours during early activation. Numeric labels follow single cells that are confidently tracked and used for analysis.

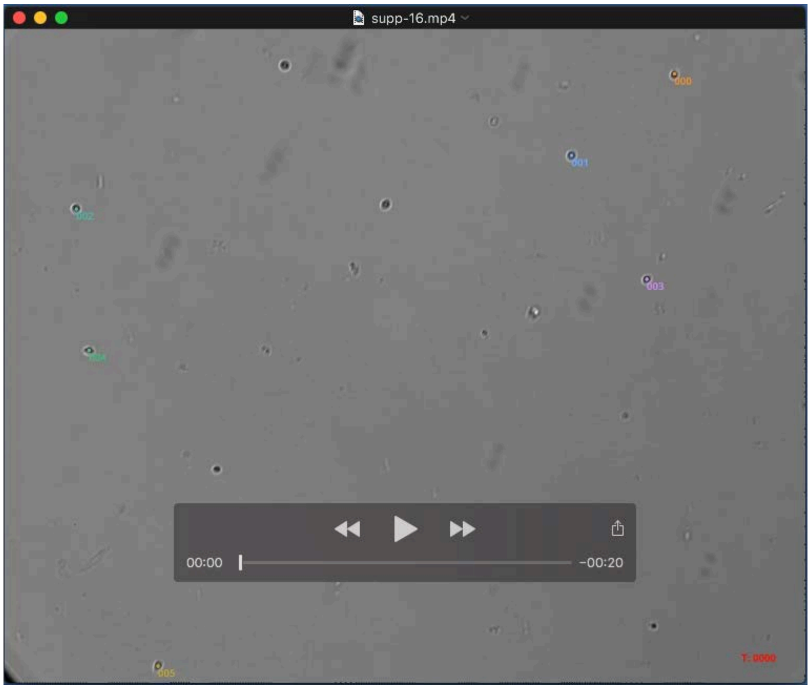

Movie 4 Muscle stem cells from an aged animal, tracked for 25 hours during early activation. Numeric labels follow single cells that are confidently tracked and used for analysis.

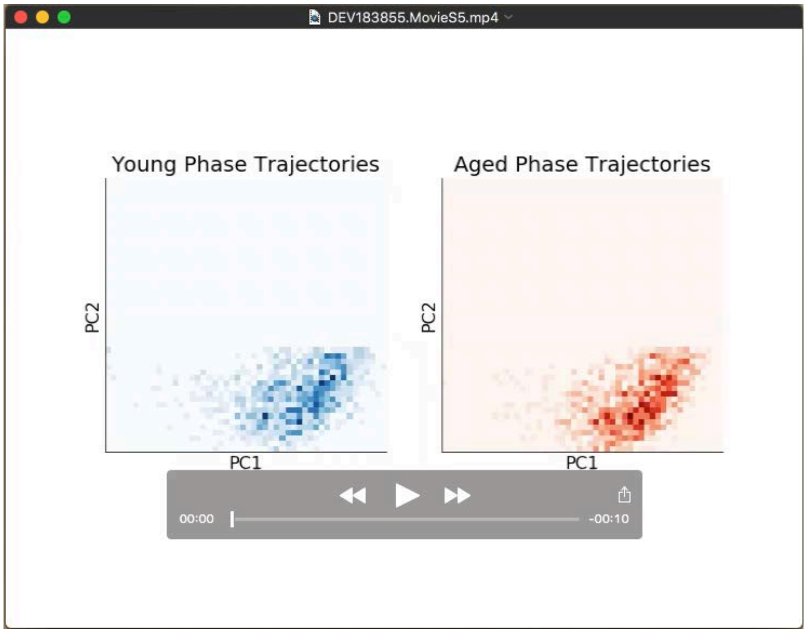

Movie 5 Heatmaps representing the density of phase points over time in transcriptional space during phase analysis simulations in young (left) and aged (right) velocity fields. Transcriptional space is represented using the first two principal components. Darker colors indicate higher cell densities for both color maps.

2 Supplementary Figures

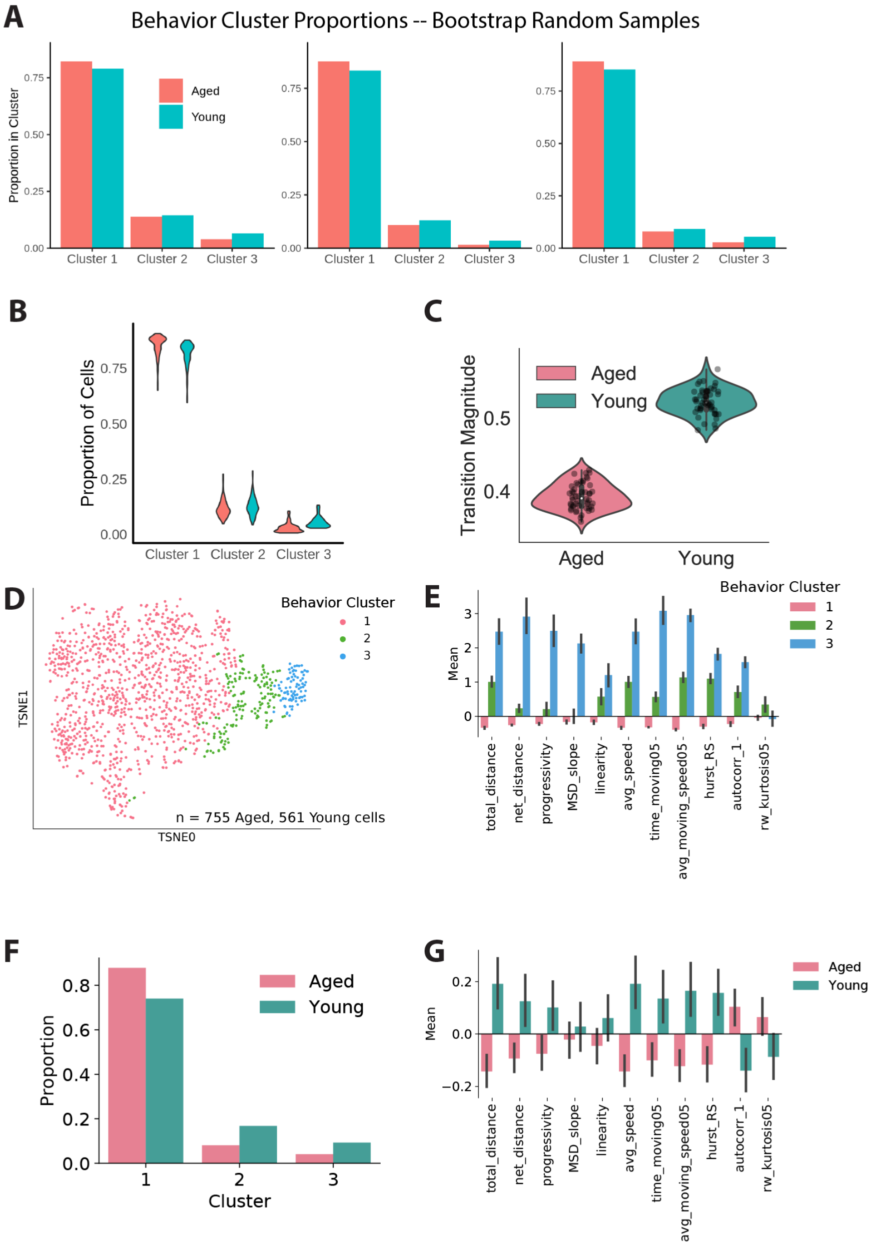

Figure S1: **Cell behavior analysis reveals aged MuSCs prefer less motile states.** **(A)** Proportion of young and aged MuSCs in each of three motility clusters across 3 bootstrap samples. Each bootstrap sample is representative of young and aged proliferative history ratios and contains roughly 60% of all observed cells. Cluster 1 is the least motile, Cluster 3 is the most motile. Motility clusters were defined independently for each bootstrap sample. **(B)** Proportion of young and aged MuSCs in each of three motility states across 100 bootstrap samples. **(C)** Mean behavior state transition magnitudes computed across 100 bootstrap samples. Young MuSCs consistently have higher transition magnitudes than aged MuSCs. **(D)** We repeated the experiment in Fig. 1 using young (3 m.o.) and aged (23 m.o.) wild-type C57Bl/6 mice. Cells were tracked for 32 hours, beginning 15 hours after plating. t-SNE projection of behavior features from a second independent behavior experiment. Points are cells, with colors indicating Behavioral Clusters. 755 aged and 561 young cells were tracked. **(E)** Behavior feature centered and scaled mean values for clusters in **D**. Behavior Cluster 1 is immotile, Behavior Cluster 2 is semi-motile, and Behavior Cluster 3 is highly motile. **(F)** Proportion of young and aged MuSCs in each Behavioral Cluster in the second independent experiment. Young MuSCs are significantly enriched in more motile clusters (χ^2 test, $p < 0.01$). **(G)** Behavior feature centered and scaled mean values for cells of each age. Young cells travel a larger total distance and have higher average speeds.

Development • Supplementary information

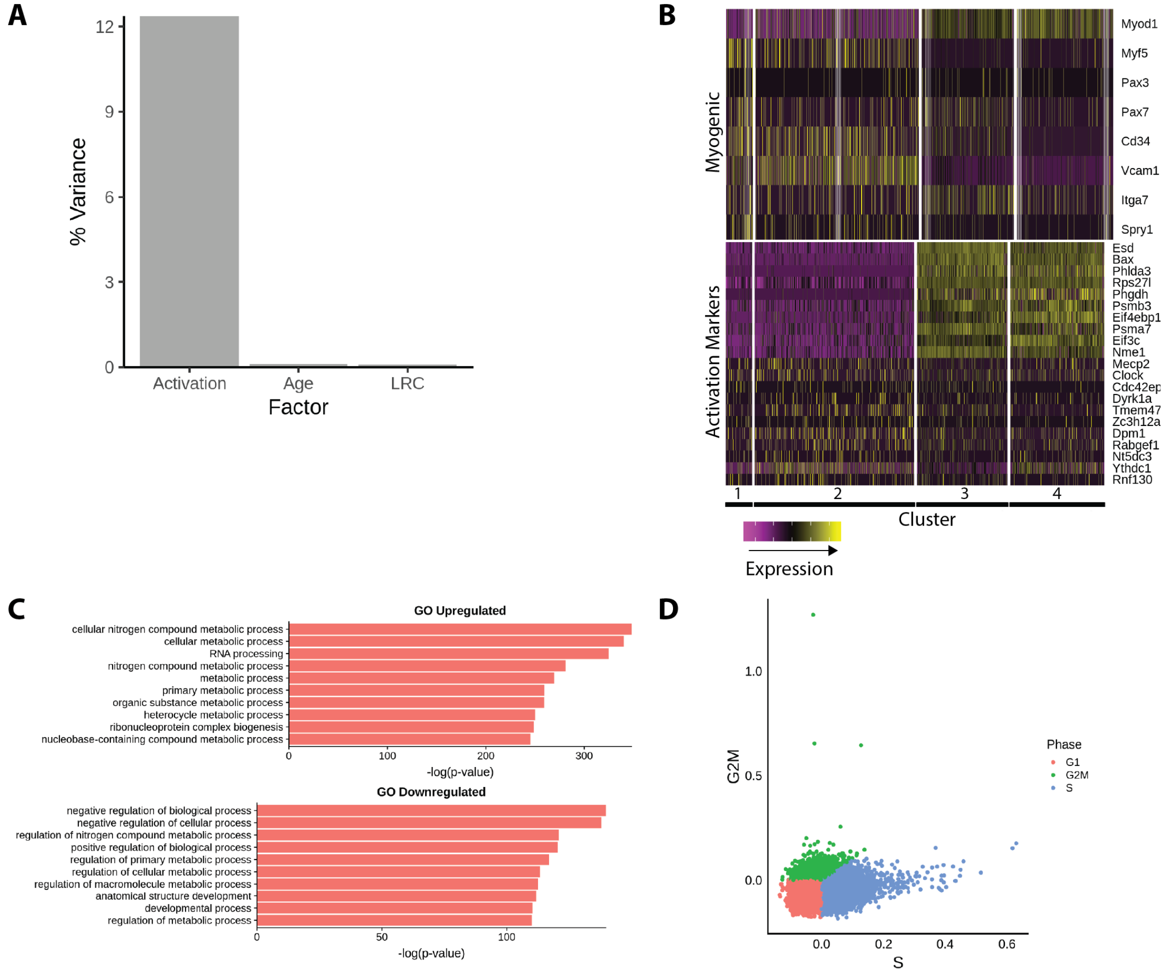

Figure S2: **Myogenic activation engages biosynthetic transcriptional programs.** **(A)** Proportion of variation in transcriptomes explained by each of the 3 factors in the single cell RNA-seq experiment: Age, proliferative history (LRC status), and Activation state (time in culture, 0 hr or 18 hr). Activation is a much greater source of variation than aging or proliferative history. **(B)** Heatmap of myogenic genes and activation markers across transcriptional clusters. Expression is mean centered and variance scaled for each gene. **(C)** GO enrichment analysis for genes upregulated and downregulated in activation relative to quiescence. **(D)** Cell Cycle scoring demonstrating little difference in cycle state across our population.

Development • Supplementary information

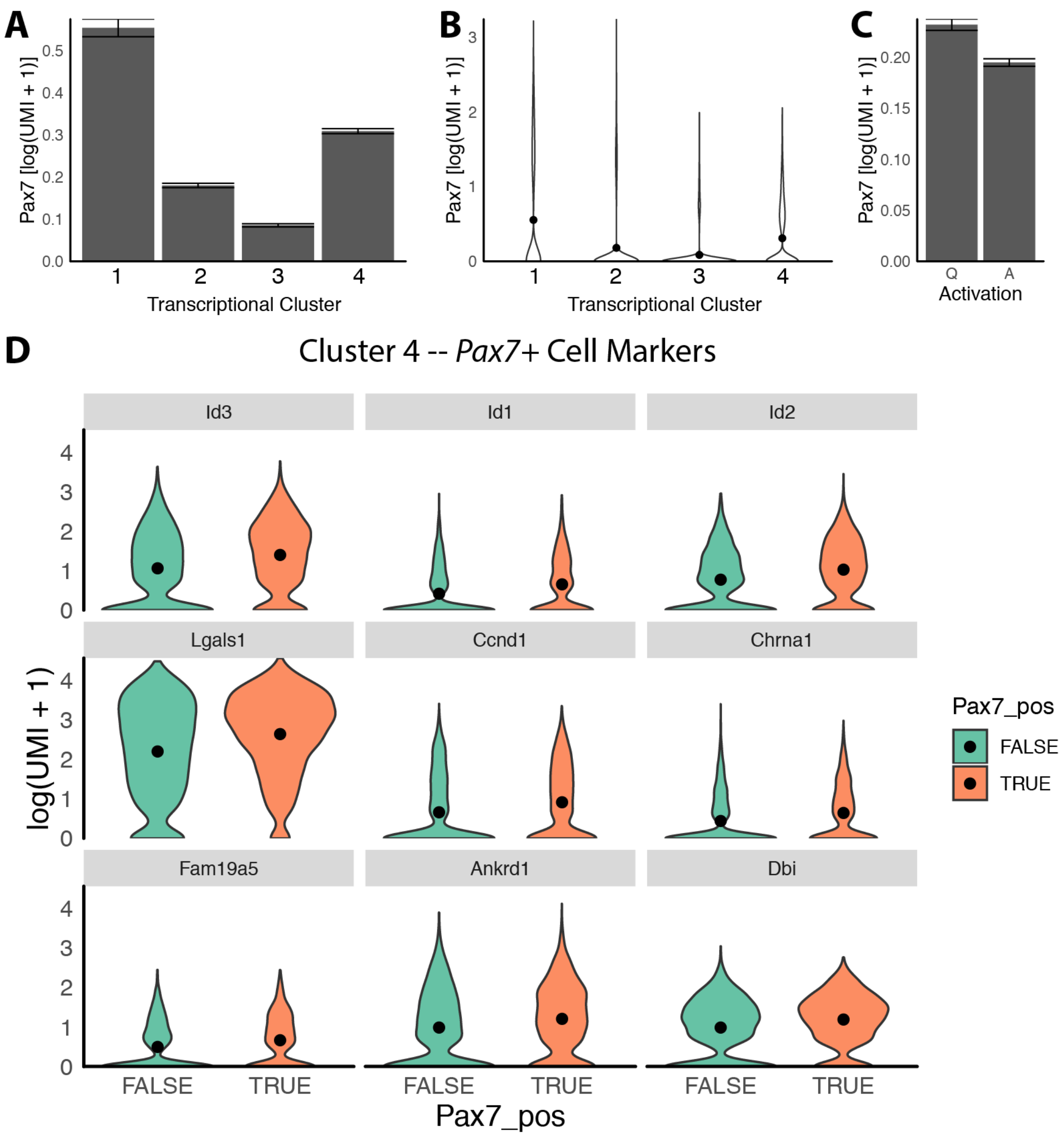

Figure S3: ***Pax7* is lower in activated MuSCs than freshly-isolated MuSCs.** (**A**) Expression of *Pax7* in each transcriptional cluster, ordered by pseudotime. *Pax7* expression decreases in early activation (Cluster 3, 0, 1), then increases slightly in the final activation cluster (2). **(B)** Data from **(A)**, presented as violin plots. **(C)** Expression of *Pax7* in quiescent, freshly-isolated MuSCs and activated MuSCs after 18 hours *in vitro*. Considering all cells at each timepoint, *Pax7* expression is decreased in activated cells. **(D)** Significant differentially expressed genes within the *Pax7*+ population of the activated Transcriptional Cluster 4 ($q < 0.05$, Wilcoxon Rank Sums). We found inhibitors of myogenic differentiation *Id1, 2, 3* and cell cycle gene *Ccnd1* were enriched in *Pax7*+ cells.

Development • Supplementary information

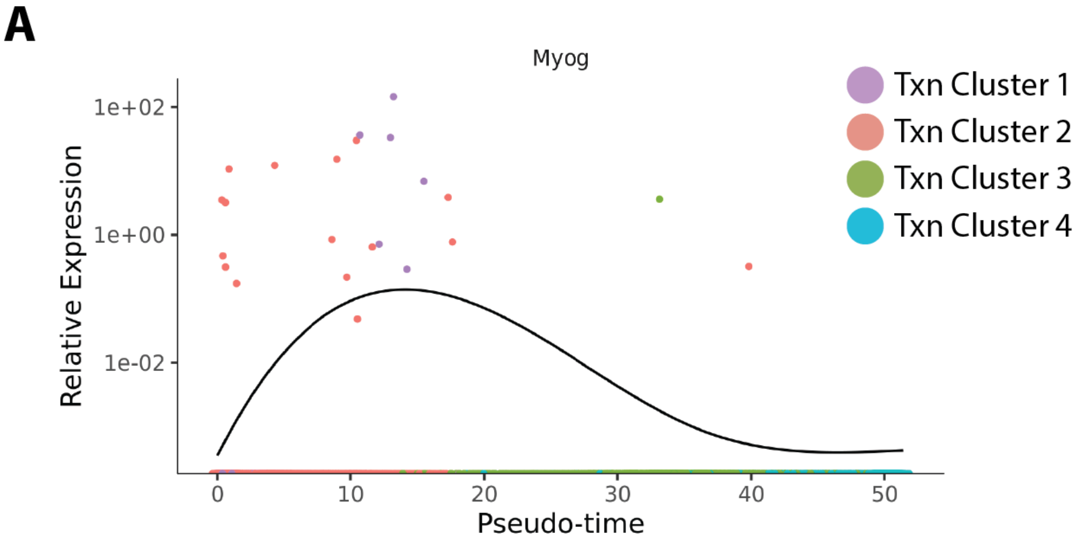

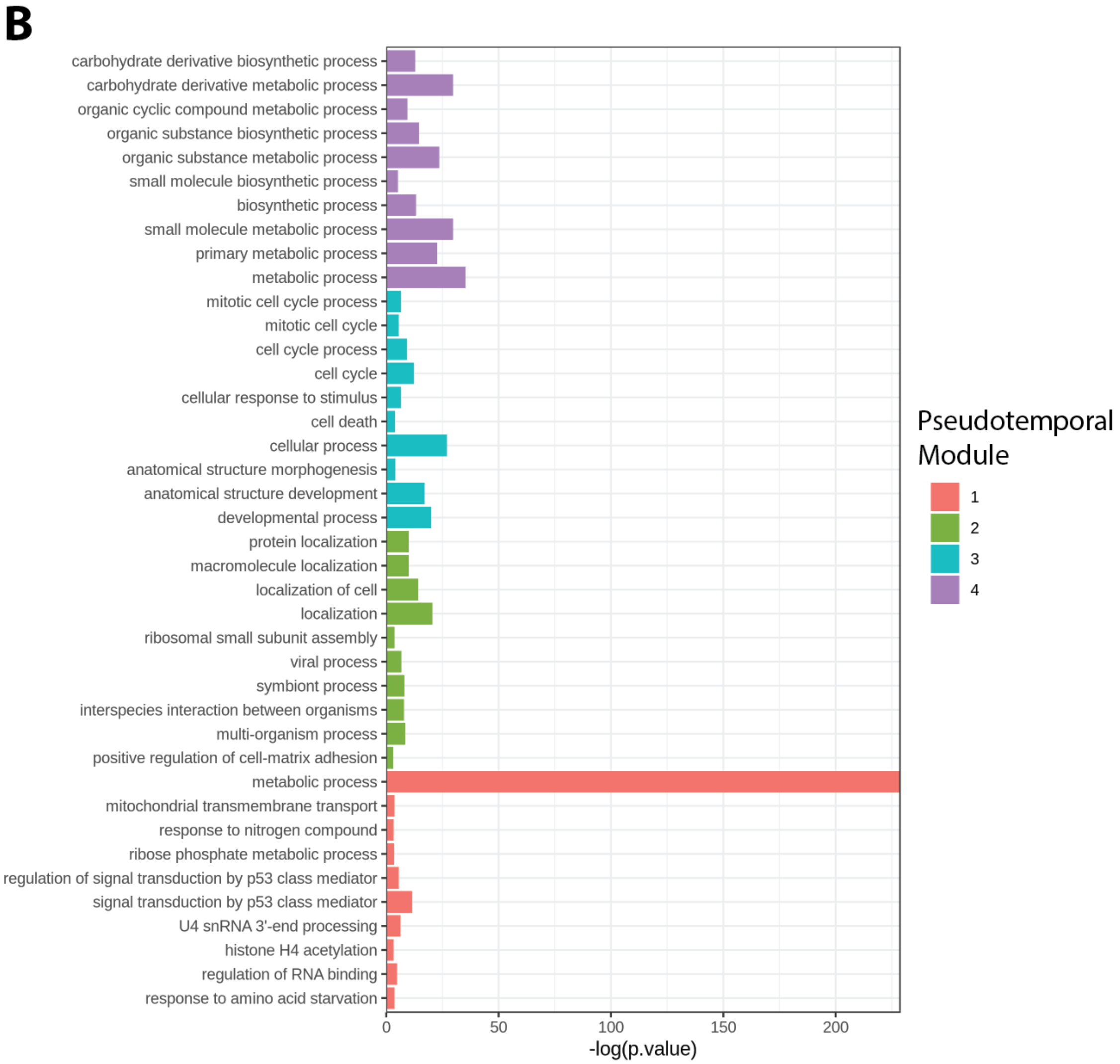

Figure S4: **Pseudotemporal analysis identifies gene modules with coordinated kinetics.** **(A)** Spline fit of *Myog* expression over pseudotime. Only 25 cells in the experiment express *Myog* at detectable levels. **(B)** Gene ontology enrichment analysis for Pseudotemporal Modules, suggesting coherent groups of co-regulated genes.

Development • Supplementary information

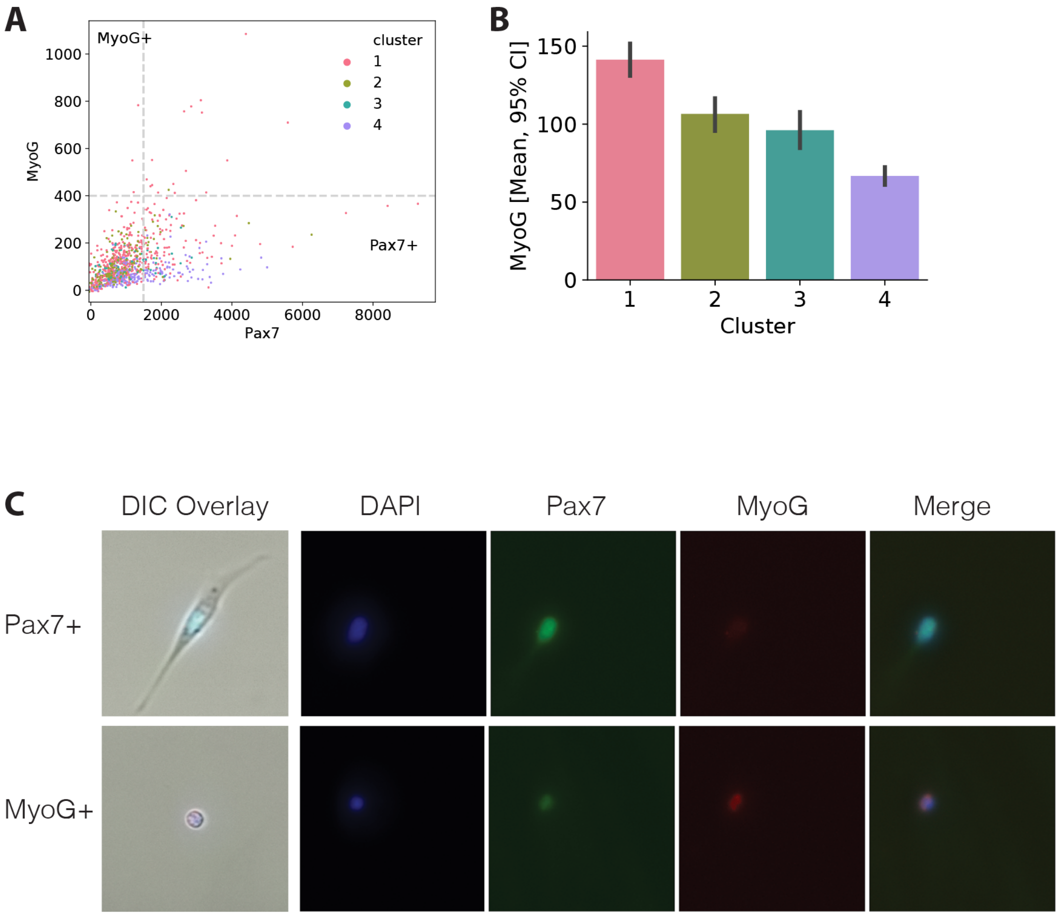

Figure S5: **MuSC behavior states are associated with myogenic transcription factor levels.** **(A)** Pax7 and MyoG intensities for cells with a paired cell behavior recording. Intensities are presented as the median pixel intensity inside the cell nucleus. Pax7 and MyoG protein are rarely co-expressed, as expected. **(B)** MyoG protein intensity (cell median) in each cell behavior cluster. MyoG levels decrease slighly with activation, though all clusters show low expression. **(C)** Representative images of Pax7/MyoG staining in cells after timelapse imaging. Panels on the far left are the final DIC image from the timelapse, registered and overlaid with fluorescent immunostains. Remaining panels are fluorescence images prior to registration. Fluorescence images are equitably rescaled across cells within each channel for presentation.

Development • Supplementary information

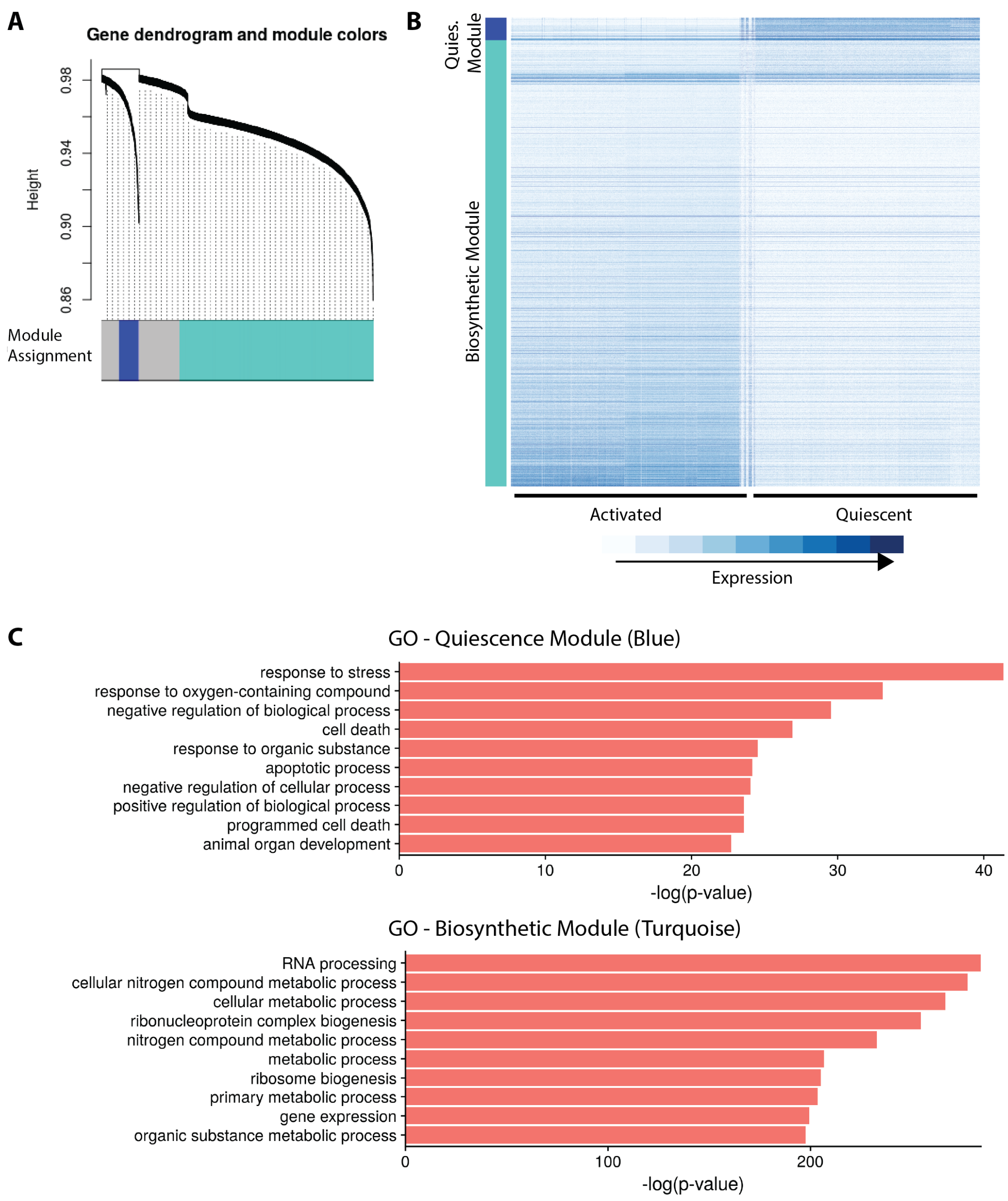

Figure S6: **Weighted gene correlation network analysis.** **(A)** Module identification in the weighted gene correlation network, with significant modules indicated by colored labels. **(B)** Heatmap of genes in the identified Quiescence and Biosynthetic modules in quiescent and activated cells. **(C)** Gene ontology enrichment analysis for genes in the identified Quiescence and Biosynthetic modules. The Quiescence module is notably enriched for stress response and cell death regulation genes, while the Biosynthetic module is enriched for RNA and protein biosynthesis.

Development • Supplementary information

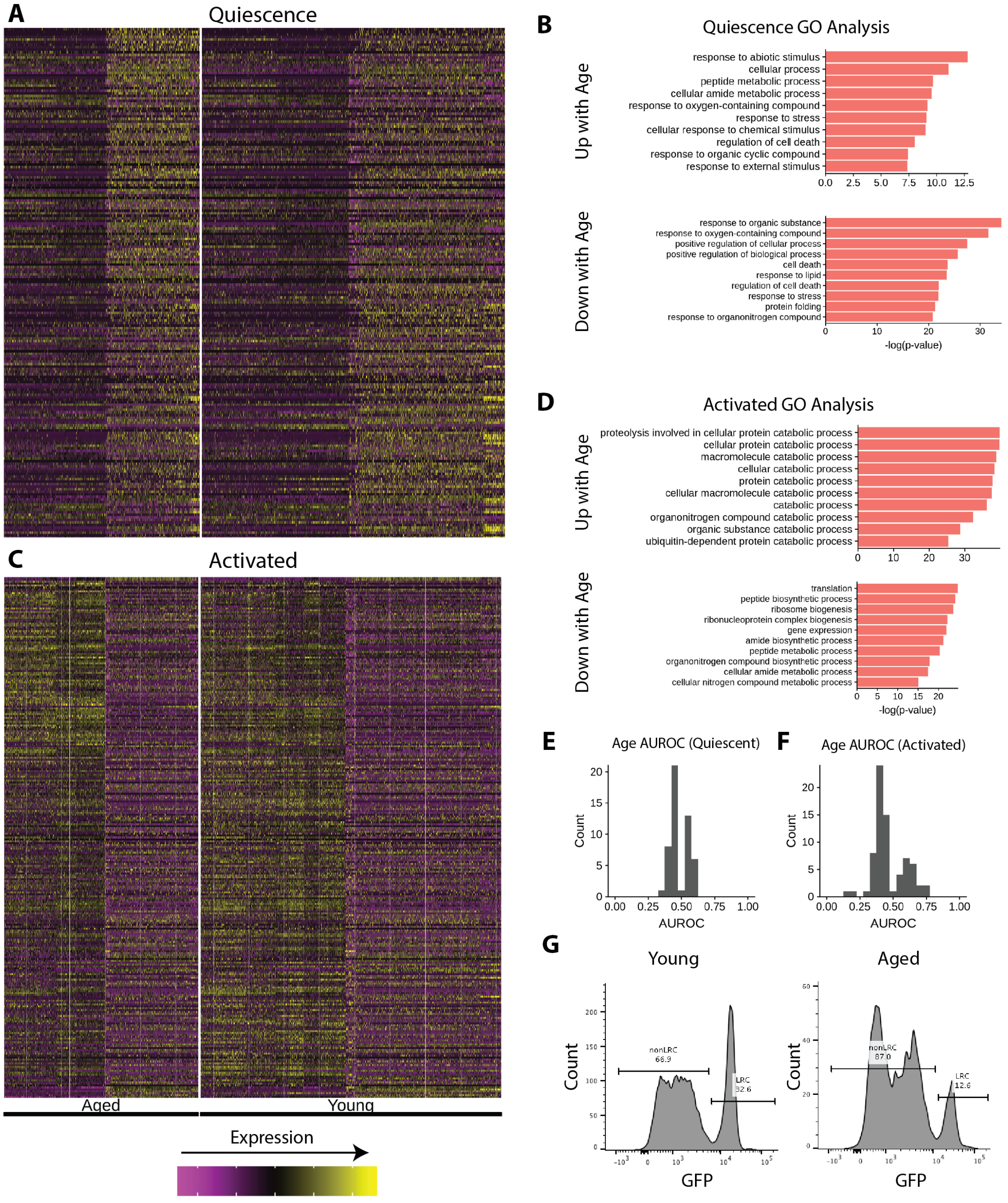

Figure S7: **Aged MuSCs display differential activation responses in many genes.** **(A)** Heatmaps of differentially expressed genes between aged and young quiescent MuSCs, and **(C)** activated MuSCs. As indicated by the heatmaps, there are no individual markers with high discriminatory ability, and activation increases transcriptional differences between aged and young MuSCs. Expression is mean centered and variance scaled for each gene. **(B)** Gene ontology enrichment analysis for differentially expressed genes between aged and young quiescent MuSCs. **(D)** Gene ontology enrichment analysis for differentially expressed genes between aged and young activated MuSCs. **(E)** Distribution of age classification AUROC scores for individual genes in quiescent and **(F)** activated MuSCs. **(G)** Representative flow cytometry measurements of the proportion of LRC (GFP high) and nonLRC (GFP low) MuSCs.

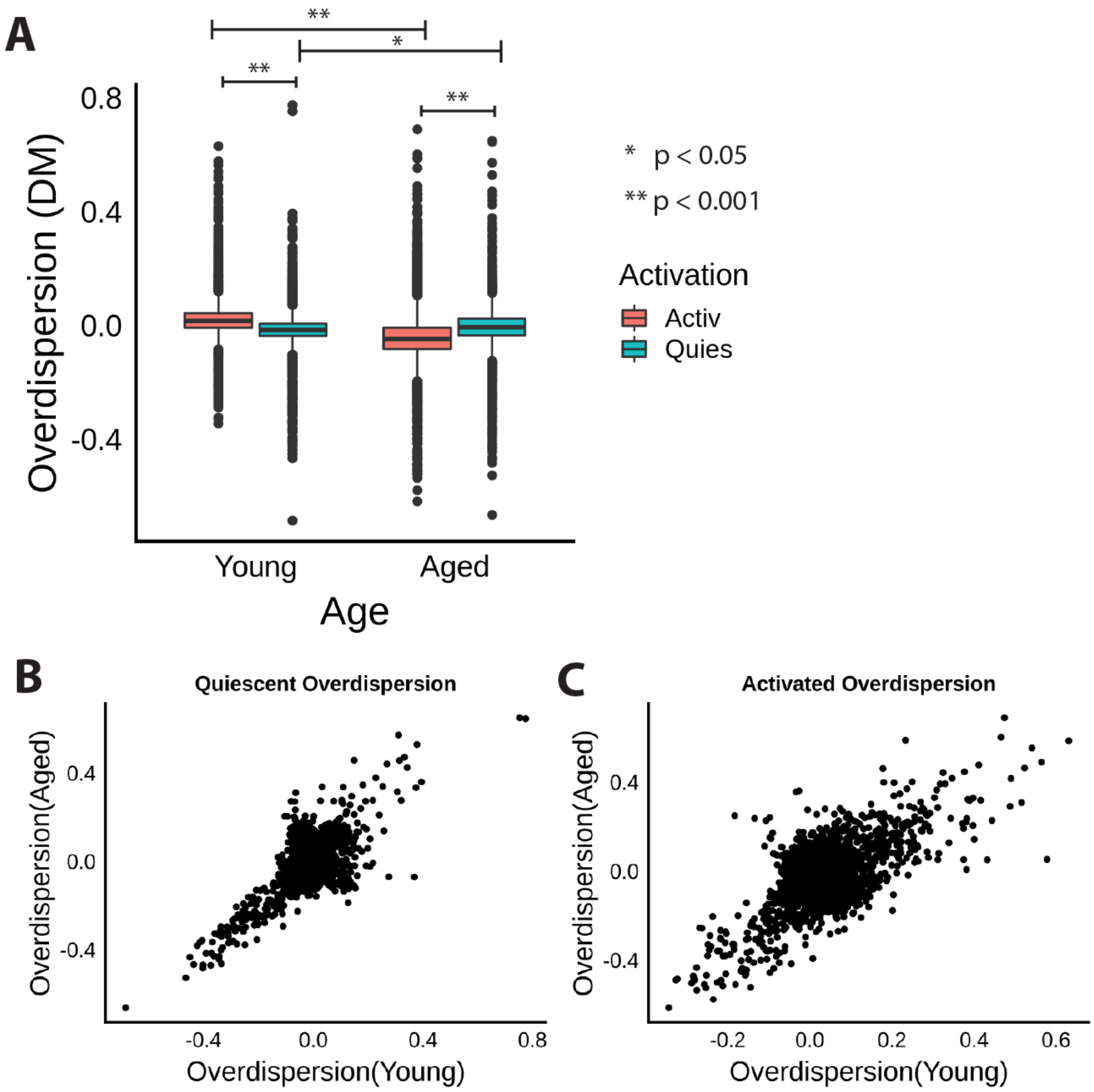

Figure S8: **Aging changes gene expression variation in a context dependent manner.** **(A)** Overdispersion distributions for young and aged MuSCs in both quiescent and activated conditions. Each underlying point represents the overdispersion estimate for a single gene. Gene expression variance increases with activation in young cells, but decreases with activation in old cells. **(B)** Comparison of overdispersion estimates for each gene between young and aged cells in quiescent and **(C)** activated conditions in quiescent and activated cells.

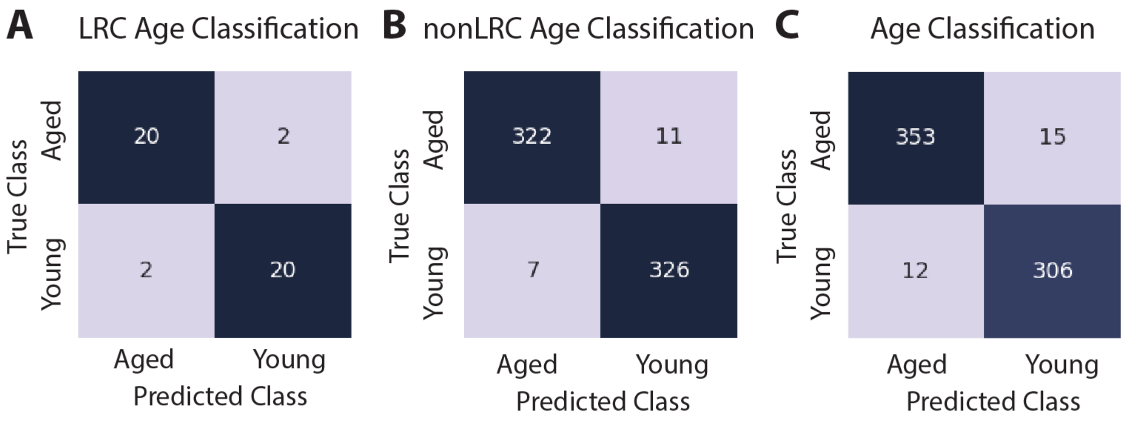

Figure S9: **Confusion matrices for age classifiers trained on LRC and nonLRC populations.** **(A)** Confusion matrices for classification of age in activated LRCs, **(B)** nonLRCs, and **(C)** the total MuSC pool using our feature selection pipeline and support vector machine classifier. Cell count values shown are for the held-out test set only. We find robust classification in all conditions.

Development • Supplementary information

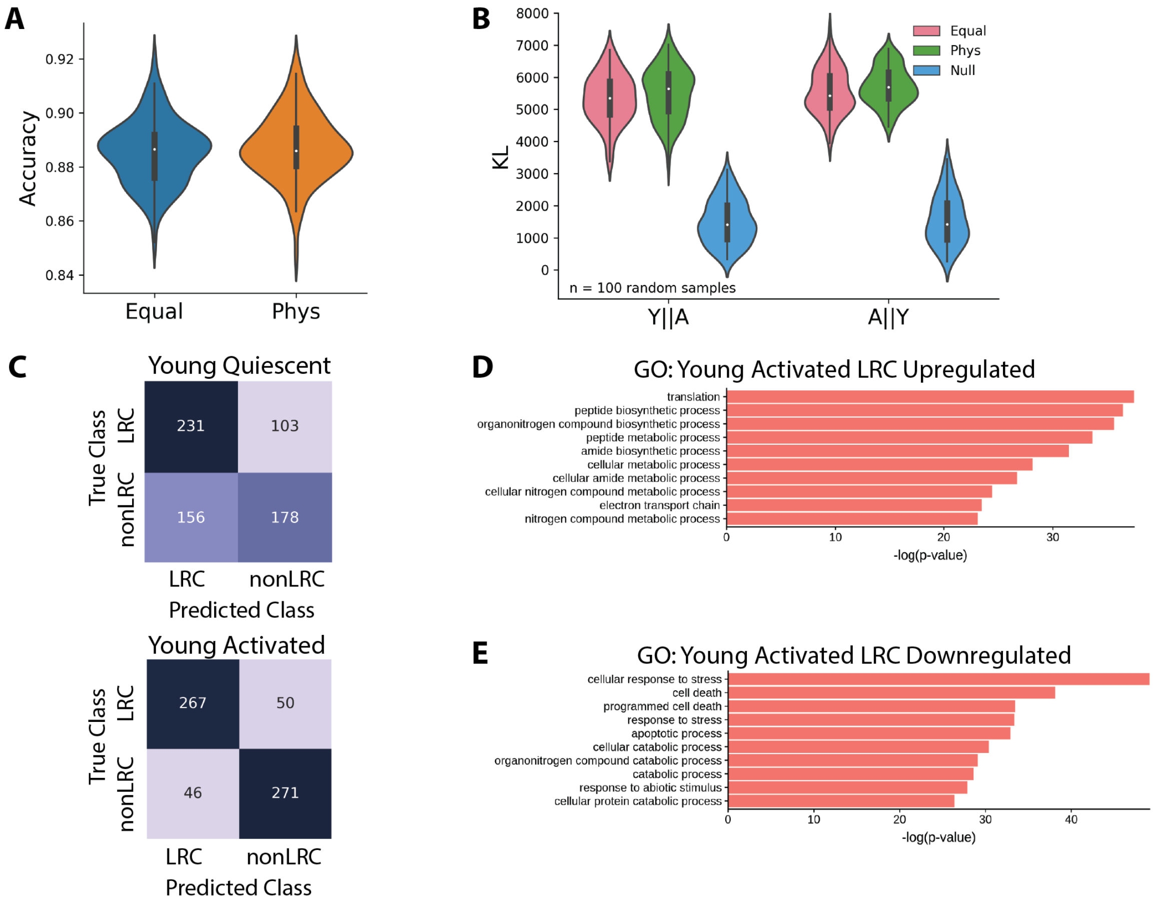

Figure S10: **LRCs and nonLRCs are discriminated by small differences in many genes. (A)** Classification accuracy for a probabilistic neural network classifier trained to discriminate young vs. aged MuSCs with either (1) equal ratios of LRC:nonLRC (35:65) at both ages or (2) physiologically observed ratios (35:65 in young, 15:85 in aged). Classification accuracy is equal between the two, suggesting that a change in LRC ratios is a minor contribution to the "magnitude" of aging. Accuracies presented are the mean of a 5-fold cross-validation split. **(B)** Estimated Kullback-Leibler divergence between young and aged MuSCs in random samples with either (1) equal LRC:nonLRC ratios (35:65) or (2) physiologically observed ratios. The divergence is equal in both contexts in both directions (the KL divergence is asymmetric) suggesting that changes in LRC proportions do not dramatically alter the magnitude of transcriptional change with age. **(C)** Confusion matrices for LRC:nonLRC classifiers. Confusion is higher in the quiescent condition. **(D)** GO enrichment analysis in young MuSCs for terms upregulated in LRCs and **(E)** downregulated in LRCs.

Development • Supplementary information

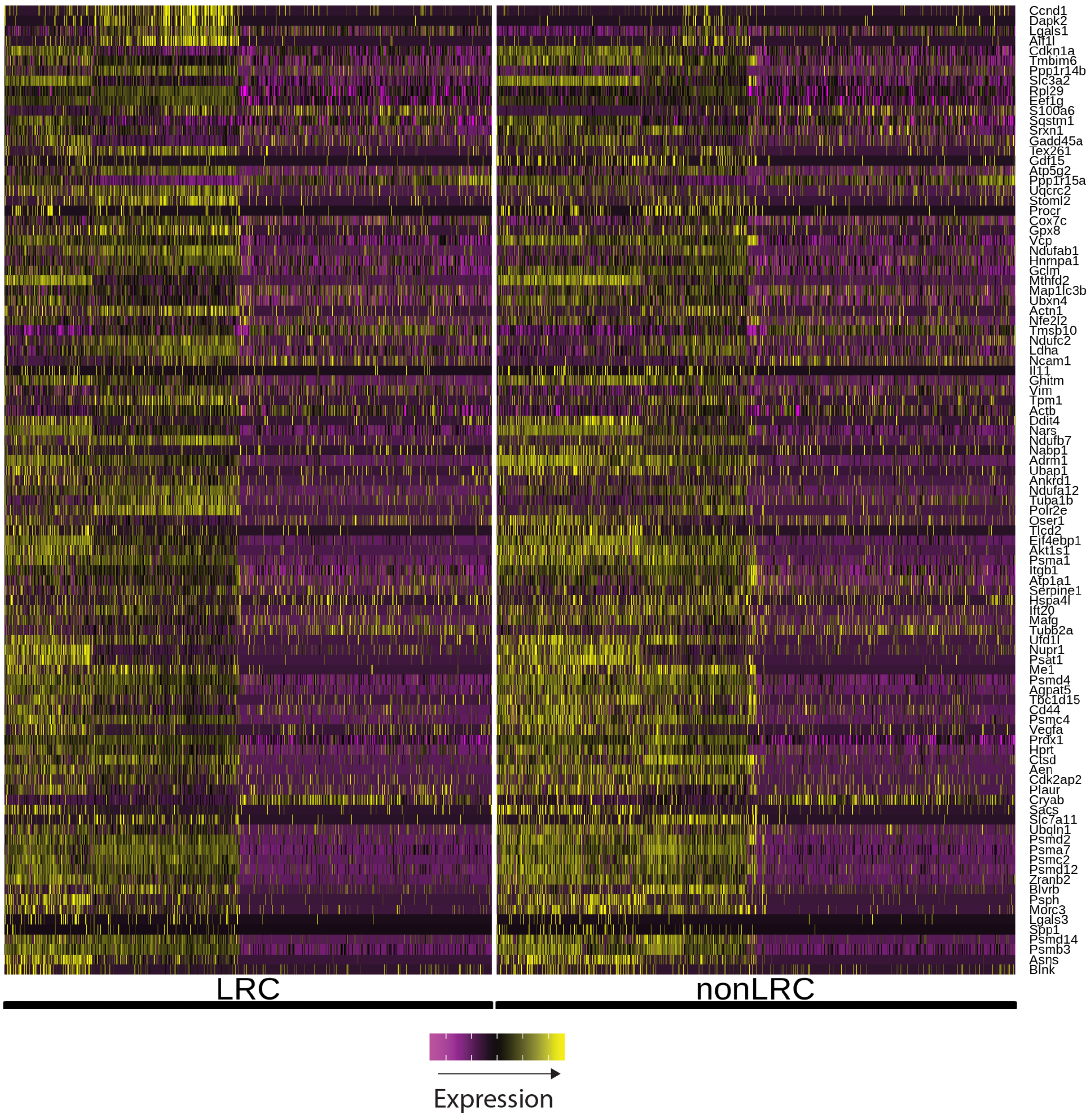

Figure S11: **LRCs and nonLRCs are discriminated by small differences in many genes.** Heatmap presenting differentially expressed genes between young LRCs and nonLRCs. We note that *Ccnd1* is upregulated in LRCs relative to nonLRCs. Expression is mean centered and variance scaled for each gene.

Development • Supplementary information

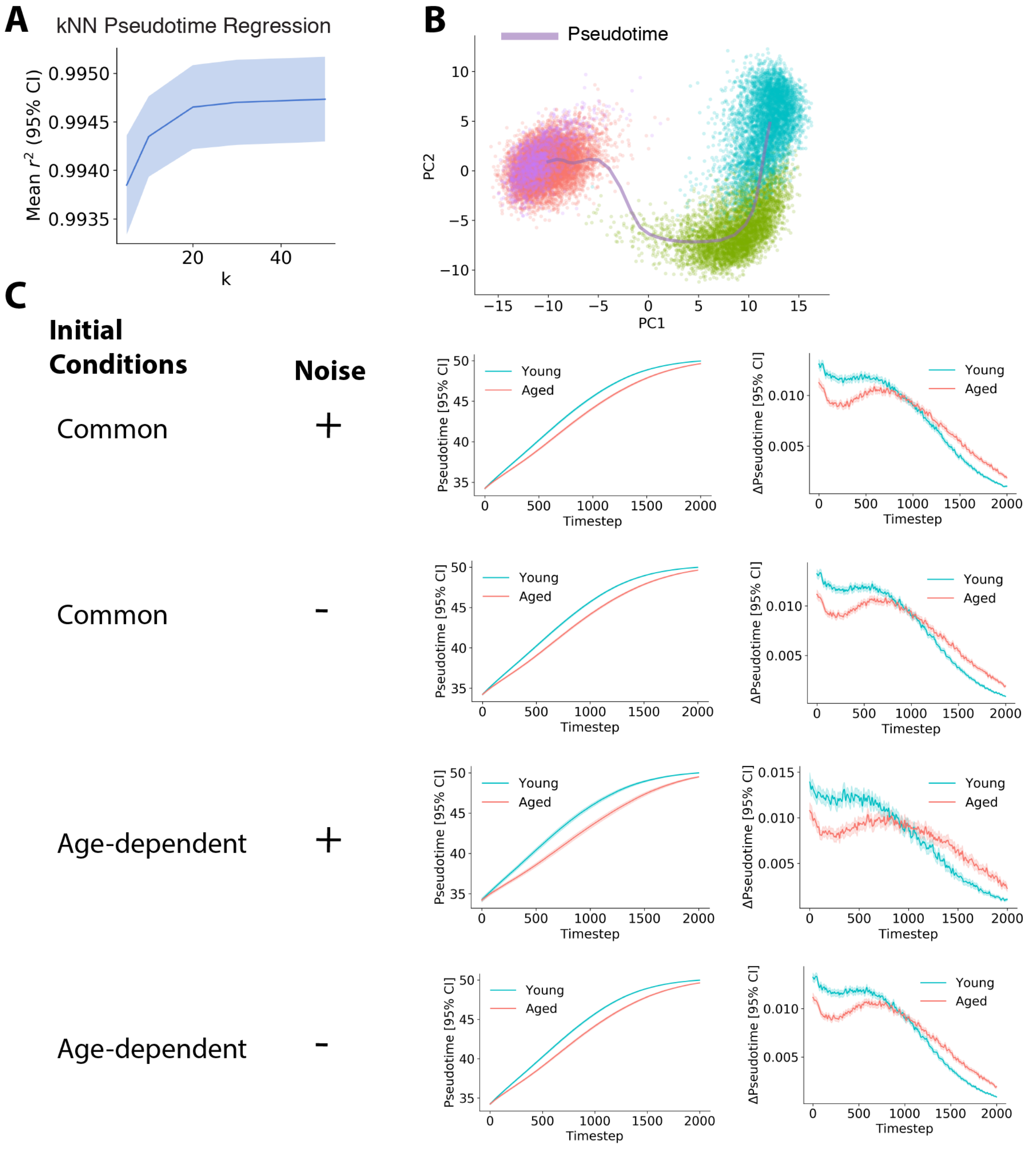

Figure S12: **Phase point simulations reveal differences in state dynamics between aged and young MuSCs.** **(A)** Prediction accuracy measured using Pearson's r^2 for a k-nearest neighbors regression model mapping transcriptional principal component scores to pseudotime values. Validated with 5-fold cross-validation. Data are presented as the mean and 95% confidence interval. **(B)** Visualization of the pseudotime curve in PCA space, estimated by computing a rolling mean of PC scores across the 1D pseudotime coordinate. Colors represent transcriptional state clusters. **(C)** Progression through pseudotime for young and aged phase points simulated with varying model parameters. Initial conditions were either (1) "common," sampled from all observed cell positions in transcriptional space, with each initial condition simulated using aged or young velocities, or (2) "age-dependent," where all intial conditions simulated using young velocities were sampled exclusively from positions observed among young cells, and vice-versa. Simulations either included Gaussian noise η scaled by the standard deviation of velocity in the local neighborhood, or no noise.

Development • Supplementary information

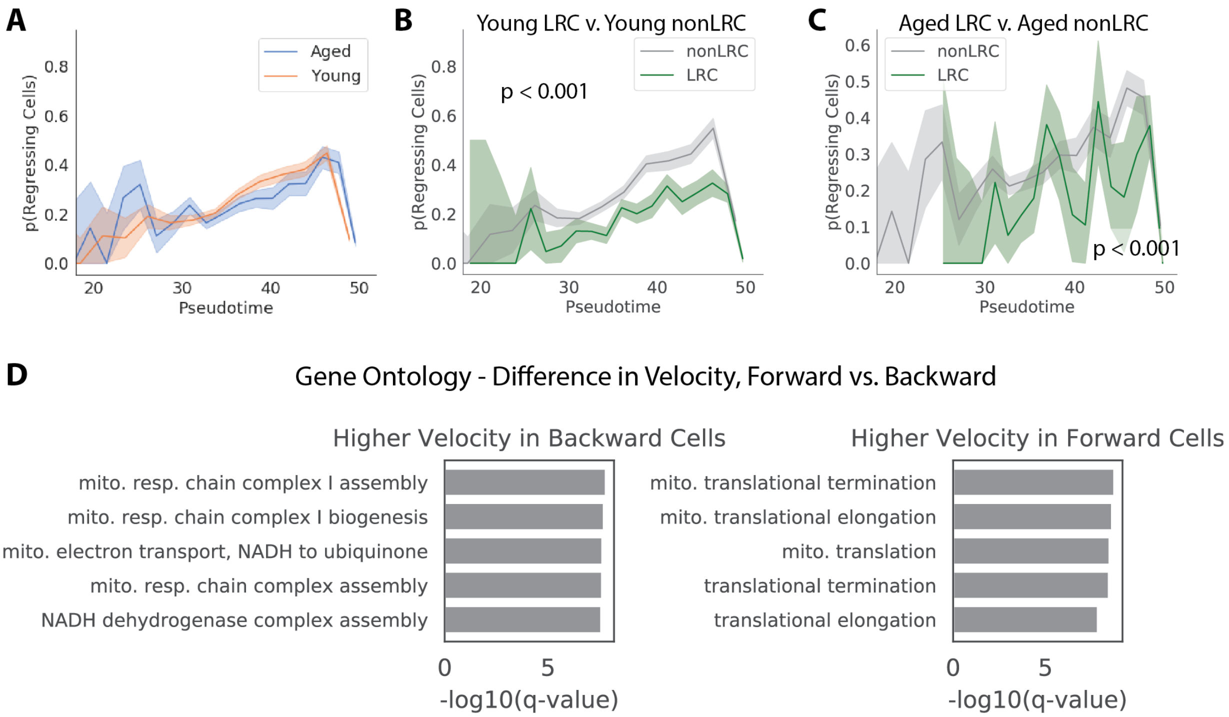

Figure S13: **Lineage regression frequency with age (A)** The proportion of "regressing" cells across pseudotime in aged and young MuSCs, revealing little age dependence. Proportions were computed by coarse-graining pseudotime into equally sized bins. **(B)** Proportion of "regressing" cells across pseudotime in young LRCs and nonLRCs. nonLRCs regress more frequently late in the activation process (χ^2 test, $p < 0.0001$. **(C)** The difference in lineage regression frequency between LRCs and nonLRCs is also present in MuSCs from aged animals (χ^2 test, $p < 0.0001$). **(D)** We computed the difference in velocity for each gene between cells moving "forward" and "backward" in the activation trajectory. Gene Ontology enrichments suggest forward moving cells have higher velocity for protein translation and biogenesis programs, while backward moving cells have higher velocity for mitochondrial oxidative phosphorylation programs.

Development • Supplementary information

3 Supplementary Tables

Table S1: **Cell counts by condition.**

Activation	Age	LRC	Cell Count
A	Aged	LRC	495
A	Aged	nonLRC	3362
A	Young	LRC	3236
A	Young	nonLRC	3636
Q	Aged	LRC	1074
Q	Aged	nonLRC	2697
Q	Young	LRC	3507
Q	Young	nonLRC	3548

Table S2: **Number of cells expressing genes of interest.**

Txn Cluster	Pax7	Spry1	Myf5	Myod1	Myog	Myf6
1	511	436	586	313	6	13
2	1125	812	2279	3346	16	27
3	516	256	442	4979	1	2
4	2041	606	551	5039	2	16

Table S3 Gene Modules with similar kinetic behavior during activation identified by pseudotime analysis.

Click here to Download Table S3

Table S4 Differentially expressed genes between young and aged MuSCs.

Click here to Download Table S4

Table S5 Differentially expressed genes between quiescent young and aged MuSCs.

Click here to Download Table S5

Development • Supplementary information

Table S6 Differentially expressed genes between activated young and aged MuSCs.

Click here to Download Table S6

Table S7 Genes used by age classifiers.

Click here to Download Table S7

Table S8 Differentially expressed genes between young LRCs and nonLRCs.

Click here to Download Table S8

Table S9 Differentially expressed genes between quiescent young LRCs and nonLRCs.

Click here to Download Table S9

Table S10 Differentially expressed genes between activated young LRCs and nonLRCs.

Click here to Download Table S10

Table S11 Differentially expressed genes between aged LRCs and nonLRCs.

Click here to Download Table S11

Table S12 Differentially expressed genes between quiescent aged LRCs and nonLRCs.

Click here to Download Table S12

Table S13 Differentially expressed genes between activated aged LRCs and nonLRCs.

Click here to Download Table S13

Table S14 Genes used by LRC classifiers.

Click here to Download Table S14

Table S15 Difference in velocity for each gene computed between forward and backward moving cells.

Click here to Download Table S15

4 Supplementary Methods

4.1 Cell Isolation

For cell isolation we used the following antibodies: anti-CD31 (Clone 390, PE-Cy7, 1:500, BD Pharmigen, 561410 AB10612003); anti-CD45 (Clone 30-F11, PE-Cy7, 1:500, BD Pharmigen, 552848 AB394489); anti-Ly6A/E (Clone D7, APC-Cy7, 1:100, BD Pharmigen, 560654 AB1727552); anti-Vcam1 (Clone 429, PE, 1:100, Invitrogen, RMCD10604 AB255657); anti-α7-integrin (Clone R2F2, APC, 1:100, Ab Labs, 67-0010-05).

4.2 Timelapse Imaging and Cell Behavior Analysis

MuSCs were imaged in 96-well plates on an incubated microscopy platform (Oko Lab) for 48 hours. Images were collected with DIC contrast every 6.5 minutes to track cell movement. Thirty rasterized fields-of-view at 20X magnification were collected from each well using an Andor Zyla 4.2 camera with pixel size of 6.5 μm. Images were segmented using a fully-convolutional DenseNet-103 neural network model, following the architecture of [1]. The model was implemented in PyTorch and trained on manually segmented images from each experiment. Code for our implementation is available at **https://github.com/jacobkimmel/fcdensenet_pytorch**. Cell tracking was performed using a custom bipartite tracking implementation that utilizes a Kalman filter motion model. Python code for our tracking implementation is available at **https://github.com/jacobkimmel/musc_tracker**.

Cell behavior was analyzed using *Heteromotility*, as previously described [2] and available at **https://github.com/cellgeometry/heteromotility**. GNU parallel was used to parallelize multiple portions of the analysis [7]. For the first experiment (Fig. 1), cells were tracked for 100 frames (25 hours) beginning 10 hours after plating. For the second experiment, cells were tracked for 131 frames (32.75 hours) beginning 15 hours after plating.

We used a set of 57 motility features from *Heteromotility*, excluding features that focus on turning angles. Features displayed in Fig. 1 include: “Total Distance,” a measure of the total distance a cell traveled; “Net Distance,” a measure of the net distance a cell traveled; “Linearity,” or the r^2 value of a linear regression through cell positions, “Rank Correlation,” as computed using a Spearman coefficient; “Progressivity,” computed as the ratio of net distance to total distance; “Mean Speed,” computed as the total distance over time; “Mean Squared Displacement,” a metric of the distance moved by a cell relative to the starting location; “Hurst Coefficient,” a description of the self-similarity between cell behaviors when modeled using a fractional Brownian motion process; “Non-Gaussian Coefficient,” measuring the degree to which cell displacements do not follow a Gaussian distribution; “Kurtosis $\tau = 1$” the kurtosis of the displacement distribution with a time lag of 1; “Kurtosis $\tau = 5$” the kurtosis of the displacement distribution with a time lag of 5; “Mean Moving Speed,” the mean speed of a cell computed only during times the cell is not stationary;

Development • Supplementary information

"Average Time Moving," the proportion of time a cell spends moving; and "Autocorrelation," a measure of self-similarity within the displacement time series.

We defined Behavior Clusters using hierarchical clustering on motility features after standard scaling and PCA dimensionality reduction, as previously described [2]. We tested the significance of Behavior Cluster preferences using a χ^2 test of the Age $\times$ Behavior Cluster contingency table. We additionally coded binary variables to represent the presence of each cell in the most motile cluster (Behavior Cluster 3), or either of the more motile clusters (Behavior Cluster 2 or 3) as defined for the full data set. We fit logistic regressions to model the likelihood of a cell being present in these more motile Behavior Clusters as a function of age and proliferative history (LRC/nonLRC) using the formulas "BehaviorCluster3   Age + LRC" and "BehaviorCluster2or3   Age + LRC" and the R function "glm", where "BehaviorCluster3" and "BehaviorCluster2or3" are the coded binary variables described above. Significance tests were performed for each coefficient using Wald's test. Both coding schemes returned similar results.

For the first behavior experiment (Fig. 1), we generated young and aged MuSC populations with representative proliferative histories *in silico* by randomly sampling LRCs and nonLRCs to match observed physiological ratios (35% LRC for young, 15% LRC for aged). For bootstrap resampling performed in Fig. S1A, B, we randomly sampled 100 populations of young and aged cells with representative LRC:nonLRC ratios (35%, 15%). From these populations, we defined Behavior Clusters and examined the relative distribution of young and aged cells across clusters. For bootstrapping analysis in Fig. S1C, we randomly drew samples of young and aged cells with representative LRC/nonLRC ratios as above, using only 80% of the cells in each population for each sample. We then computed mean transition vector magnitudes for aged and young samples. We repeated this procedure 100 times to estimate the variability in mean transition vector magnitude measurements.

4.3 Paired Immunohistochemistry and Timelapse Imaging

For paired behavior-immunocytochemistry experiments, cells were fixed in 4% paraformaldehyde for 10 minutes immediately following the imaging timecourse. All steps were carried out at room temperature, unless otherwise noted. Cells were washed in PBS 3X, using gentle pipette aspiration (without vacuum) to remove buffer. We found that vacuum aspiration tends to dislodge a large number of cells. Cells were subsequently permeabilized with 0.2% PBSX (PBS + Triton X-100) in two 5 minute washes. After permeabilization, cells were blocked in 10% goat serum in PBSX for 60 minutes. We added primary antibodies for Pax7 (Mouse, Developmental Studies Hybridoma Bank) and MyoG (Rabbit, Santa Cruz Biotechnology Cat sc-576 AB 2148908) at 1:100 concentrations in 10% goat serum/PBSX overnight at 4°C. Cells were washed 4X in PBSX, then blocked a second time by incubation in 10% goat serum/PBSX for 60 minutes. Cells were incubated with secondary antibodies anti-mouse Alexa 488 (Thermo Fischer) and anti-rabbit Alexa 647 (Thermo Fischer) for one hour. Cells were finally washed 3X in PBSX, 5 min each, then 3X in PBS, 5 min each, incubated with Hoescht 33342 (5 μg/mL in PBS) for 10 minutes, and washed with PBS again.

4.4 EdU Incorporation Statistical Testing

We tested the significance of differences in EdU incorporation by fitting logistic regression models of the form: EdU $\sim$ Mouse + LRC and EdU $\sim$ Mouse + Pax3, where EdU is a binary response variable indicating the EdU status of each cell (+/-), Mouse is a dummy-coded categorical covariate indicating the animal of origin for each cell, and LRC, Pax3 are binary covariates indicating the LRC and Pax3 status of each cell respectively. These coefficients in these regressions account for the specific effect of LRC and Pax3 status on EdU incorporation, controlling for animal of origin. Regressions were fit with the **glm** function in **R**. We tested coefficient significance using Wald's test.

4.5 Contribution of experimental factors to transcriptional variation

Linear models were fit for each gene in the form:

$$\text{NormCount}_g(c) = \beta_1\text{Activation}(c) + \beta_2\text{LRC}(c) + \beta_3\text{Age}(c)$$

where Activation, LRC, and Age are binary vectors indicating the activation status, LRC status, and age of each cell, c is a cellular index, and g is a gene index. The proportion of variance attributable to each of these factors was calculated using an analysis of variance (ANOVA).

4.6 Overdispersion Analysis

We define an overdispersion score DM as:

Development • Supplementary information

$$\mathrm{DM(g)} = \log_{10}(\mathrm{CV}^2) - f(g)$$

where g is a gene in the set of analyzed genes G and $f(\cdot)$ is a rolling median. We compute the rolling median on the mean expression ($\log_{10}(\mu)$) vs. coefficient of variation squared ($\log_{10} \mathrm{CV}^2$) plot using a bin size of $n = 50$ genes and a stride of $s = 1$ along the mean expression axis and calculating the median $\log_{10} \mathrm{CV}^2$ of each bin. An additional parameter α was computed as the proportion of cells expressing a given gene (elsewhere referred to as the "proportion of non-zero cells").

4.7 Estimation of LRC to nonLRC contribution to transcriptional change

We estimate the "magnitude" of transcriptional change with aging between a set of young and aged transcriptomes by training probabalistic classifiers to estimate the density ratio between distributions of young and aged transcriptomes. This method is commonly employed in machine learning and is known as the "Density Ratio Trick" [6, 5].

We generate populations of aged and young MuSCs by random sampling with $n = 1000$ cells per age. We sample populations with either physiologically observed LRC:nonLRC ratios (35:65 young, 15:85 aged) or equal ratios for both ages (35:65 young, 35:65 aged). The latter sampling scheme simulates a condition where LRC proportions do not change with age. Fully-connected neural networks with 3 hidden layers, each containing 100 hidden units are trained to output probabilities that a given transcriptome is either young or aged using a softmax activation. Networks are trained using a crossentropy objective and the Adam optimizer with the *scikit-learn* implementations. Networks were trained for a maximum of 1000 epochs using early stopping with a patience of 50 epochs using 10% of training data as test data for model selection. Minibatch sizes of 128 transcriptomes were used. Training was performed using 5-fold cross-validation, such that each predicted probability for a given cell was produced using a classifier that did not see that cell during training. All training parameters were chosen empirically without hyperparameter optimization.

Once trained, these probabilistic classifiers output a probability $p(x)$ that a given cell x comes from the distribution of young transcriptomes, as well as a probability $q(x) = 1 - p(x)$ that the cell comes from the distribution of young transcriptomes. The "density ratio" for each cell is simply the ratio of these two probability distributions.

$$r(x) = \frac{q(x)}{p(x)}$$
$$\log r(x) = \log q(x) - \log p(x)$$

From this ratio, the Kullback-Leibler (KL) divergence can be estimated:

$$\mathcal{D}_{\mathrm{KL}}(p(x)||q(x)) = -\sum_{x \in X} p(x) \log r(x)$$

We use this estimate of the KL divergence as a measure of the magnitude of difference between young and aged transcriptomes. Because the KL divergence is asymmetric, we present the divergence measures for both directions ($\mathcal{D}_{\mathrm{KL}}(p(x)||q(x))$) and $\mathcal{D}_{\mathrm{KL}}(q(x)||p(x))$)). An estimate of the divergence obtained simply due to cell-cell variation within each age is computed by training classifiers on two random samples of young cells or two random samples of aged cells, where classification should perform poorly as both samples are drawn from the same distribution. As expected, our estimate of the KL divergence from these null classifiers is significantly lower than the estimates we find in classifiers trained to discriminate young from aged random samples with either physiological or equal LRC:nonLRC ratios.

4.8 Support Vector Machine Classification

Support vector machine (SVM) classification models to discriminate cell age and LRC status were trained using **scikit-learn** implementations [4]. For age classification, activated MuSCs were subsampled to match the physiologically observed LRC:nonLRC ratio across ages (young, 35:65; aged, 15:85). The total count matrix was split by random sampling into a 10% held out validation set and a 90% train/test set. "Chrono-variant" genes were identified based on *only* the train/test set to avoid information leakage from the validation set. All genes that showed a > 0.1 fold change on a natural log scale and were expressed in at least 3 cells were considered chrono-variant, yielding 667 genes. SVM classification models were trained with L_1 regularization to enforce sparsity. Regularization strength $\lambda \sim C^{-1}$ was optimized by performing a line search using 5-fold cross-validation within the train/test set. The number of non-zero weight coefficients in each trained, L_1-regularized classifier was considered to be the number of genes utilized by that

Development • Supplementary information

classifier. Validation accuracies were obtained by training a classifier on the entire train/test set with the optimized regularization strength, and performing prediction on the held-out validation set.

4.9 RNA Velocity Analysis and Dynamical Simulations

RNA velocity was inferred using **velocyto** [3] with default parameters. Gene expression levels were first imputed using a k-nearest neighbors approach, as outlined [3]. The magnitude of RNA velocity relative to pseudotime was quantified by binning cells along the pseudotime axis and computing the magnitude of the mean RNA velocity for each bin.

A k-nearest neighbors regression model (kNN-R) was trained on PCA embeddings for experimentally measured single cell transcriptomes and their corresponding pseudotime assignments. Using 5-fold cross validation, a range of values for k were estimated and $k = 30$ was chosen to optimize the regression r^2 while minimizing computational expense. k-NNR model fit was estimated at $r^2 > 0.96$ by 5-fold cross validation, indicating high performance for estimation.

To determine differences between aged and young velocity fields, phase point simulations were performed with numerical methods. A set of initial positions in the 2D PCA embedding for both young and aged cells was sampled from observed cellular positions. For these experiments, the set of initial positions was restricted to cells in the "activated" 18 hour time point to prevent simulations from encountering the low density region between quiescent and activated cells where we have little information for velocity inference. Additionally, we restrict initial positions to observed cells with a PC1 embedding score < -3, which corresponds to the more primitive cells in transcriptional Cluster 2.

Phase points were initiated at positions x_0 and evolved for $t = 5,000$ timesteps. At each timestep, phase point velocity v_t was computed as the mean velocity of the $k = 100$ nearest cells to the phase point in the observed cell embeddings. For simulations in young and aged velocity fields, only young or aged cells were considered at this step, respectively. New phase point positions x_{t+1} were computed as the sum of the velocity v_t and current phase point position x_t, plus a noise term η:

$$x_{t+1} = x_t + v_t + \eta$$

where noise is drawn from a multidimensional normal distribution $\eta \sim \mathcal{N}(0, \sigma_t)$ with a standard deviation σ_t computed as the standard deviation of the velocity from the $k = 100$ nearest cells to the phase point. When specified, this noise term was set to 0 for some experiments.

4.10 Change in Pseudotime Analysis

The "change in pseudotime" (ΔPseudotime) was estimated for each cell using the k-nearest neighbors regression model. Future transcriptional states x_{t+1} were inferred by RNA velocity as above, and the pseudotimes for these states were predicted using the kNN-R model. ΔPseudotime is defined as the difference between the inferred future and measured present pseudotime for each cell:

$$\Delta p = \hat{p}_{t+1} - p_t$$

where $\hat{p}_{t+1}$ is the inferred pseudotime using RNA velocity and the kNN-R model and p_t is the observed pseudotime at the experimental timepoint.

Cells were defined to be undergoing "lineage regression" if they displayed a ΔPseudotime $< \sigma$, where σ is the standard deviation of the ΔPseudotime distribution. Code is available in http://github.com/jacobkimmel/myodyn.

References

[1] S. Jegou, M. Drozdzal, D. Vazquez, A. Romero, and Y. Bengio. The One Hundred Layers Tiramisu: Fully Convolutional DenseNets for Semantic Segmentation. In *2017 IEEE Conference on Computer Vision and Pattern Recognition Workshops (CVPRW)*, pages 1175–1183. IEEE, 2017.

[2] J. C. Kimmel, A. Y. Chang, A. S. Brack, and W. F. Marshall. Inferring cell state by quantitative motility analysis reveals a dynamic state system and broken detailed balance. *PLoS computational biology*, 14(1):e1005927–29, Jan. 2018.

[3] G. La Manno, R. Soldatov, A. Zeisel, E. Braun, H. Hochgerner, V. Petukhov, K. Lidschreiber, M. E. Kastriti, P. Lonnerberg, A. Furlan, J. Fan, L. E. Borm, Z. Liu, D. van Bruggen, J. Guo, X. He, R. Barker, E. Sundstrom, G. Castelo-Branco, P. Cramer, I. Adameyko, S. Linnarsson, and P. V. Kharchenko. RNA velocity of single cells. *Nature*, 560(7719):494–498, Aug. 2018.

[4] F. Pedregosa, G. Varoquaux, A. Gramfort, V. Michel, B. Thirion, O. Grisel, M. Blondel, P. Prettenhofer, R. Weiss, V. Dubourg, J. Vanderplas, A. Passos, D. Cournapeau, M. Brucher, M. Perrot, and É. Duchesnay. Scikit-learn: Machine Learning in Python. *Journal of Machine Learning Research*, 12(Oct):2825–2830, 2011.

[5] M. Rosca, B. Lakshminarayanan, D. Warde-Farley, and S. Mohamed. Variational approaches for auto-encoding generative adversarial networks. *arXiv preprint arXiv:1706.04987*, 2017.

[6] M. Sugiyama, T. Suzuki, and T. Kanamori. Density-ratio matching under the Bregman divergence: a unified framework of density-ratio estimation. *Annals of the Institute of Statistical Mathematics*, 64(5):1009–1044, Nov. 2011.

[7] O. Tange. GNU Parallel-The Command-Line Power Tool. The USENIX Magazine. 2011; 36 (1): 42–7, 2011.

Development • Supplementary information
