# Supplementary material for: Aging induces aberrant state transition kinetics in murine muscle stem cells
Source: Development. 2020 May 5;147(9):dev183855. doi: 10.1242/dev.183855 (PMC7225128; doi:10.1242/dev.183855)
Supplement: Reviewer comments [file develop-147-183855_review_history.pdf]

Aging induces aberrant state transition kinetics in murine muscle stem cells

Jacob C. Kimmel, Ara B. Hwang, Annarita Scaramozza, Wallace F. Marshall and Andrew S. Brack
DOI: 10.1242/dev.183855

Editor: Gordon Keller

Review timeline

Original submission:	16 August 2019
Editorial decision:	11 October 2019
First revision received:	10 January 2020
Accepted:	17 February 2020

Original submission

First decision letter

MS ID#: DEVELOP/2019/183855

MS TITLE: Aging induces aberrant state transition kinetics in murine muscle stem cells

AUTHORS: Jacob C. Kimmel, Ara B. Hwang, Wallace F. Marshall, and Andrew S. Brack

I have now received all the referees' reports on the above manuscript, and have reached a decision. The referees' comments are appended below, or you can access them online: please go to BenchPressand click on the 'Manuscripts with Decisions' queue in the Author Area.

As you will see, the referees express considerable interest in your work, but have some significant criticisms and recommend a substantial revision of your manuscript before we can consider publication. If you are able to revise the manuscript along the lines suggested, which may involve further experiments, I will be happy receive a revised version of the manuscript. Your revised paper will be re-reviewed by one or more of the original referees, and acceptance of your manuscript will depend on your addressing satisfactorily the reviewers' major concerns. Please also note that Development will normally permit only one round of major revision.

Please attend to all of the reviewers' comments and ensure that you clearly highlight all changes made in the revised manuscript. Please avoid using 'Tracked changes' in Word files as these are lost in PDF conversion. I should be grateful if you would also provide a point-by-point response detailing how you have dealt with the points raised by the reviewers in the 'Response to Reviewers' box. If you do not agree with any of their criticisms or suggestions please explain clearly why this is so.

Reviewer 1

Advance summary and potential significance to field

The manuscript titled "Ageing induces aberrant state transition kinetics in murine muscle stem cells" by Kimmel et al. assesses muscle stem cell dynamics by predominately using single cell RNA sequence and presents a unique perspective. The success of this manuscript is the combinatorial assays that provide some functional validation to their bioinformatics inferences, such as the use of

© 2020. Published by The Company of Biologists under the terms of the Creative Commons Attribution License (https://creativecommons.org/licenses/by/4.0/).

their imaging platform, EdU assays and assessing different muscle stem cell populations (label and non-label retaining cells, LRC and non-LRCs respestively). The authors broadly attempt to answer how aging affects the quiescence to activation transitions by first asking whether aged vs young satellite cells undergo different paths/states towards activation or merely different kinetics. Interestingly, aged and young muscle stem cells are transcriptomically similar, while they differ in the activation transitions. They also demonstrate that genes vary in their expression dynamics during activation: from monotonic increase and decrease to non-monotonic behaviour.
The authors also examine LRCs and non-LRCs in the context of aging and activation. Through the use of many bioinformatic packages, such as Monocle and RNA velocity, the authors conclude that muscle stem cells predominantly undergo the same transitions during aging, but this aging delays these transitions.

Comments for the author

Although this study is largely well executed, there are some questions that would be necessary to answer to further substantiate their claims. One of bigger issues is the use of in vitro conditions to trigger an activation state. While this may be necessary for their experimental design, it can also hamper the interpretation of results and their conclusions. Further details and suggestions are stated below.

Concern 1: furthering the claims of non-monotonic gene expression The authors show that Pax7 does not monotonically decrease in expression over the course of activation (Figure 2G). In parallel, they present parallel assays showing that even though motility behaviour can be ordered, Pax7 is still non-monotonic. A few concerns are raised, since other single cell analyses show the opposite: Pax7 decreases upon activation. In the Dell'Orso et al. (2019) study, they found 2 groups of MuSCs: one being Pax7Hi and MyodLow and the other being Pax7Low and MyodHigh. In another study by De Micheli et al. (2019, bioRxiv), Pax7 also drastically drops upon activation (Figure 3D). These two studies seem to conflict the findings presented here. Could the "activation" state that the authors present here be merely an in vitro artefact?
Similarly, Pax7+/MyoG+ cells are rarely observed in floating ex vivo cultured myofiber assays (Zammit et al., 2004) and the dogma suggests that MyoG+ cells are Pax7- (Yin et al., 2013). The data in Figure 3 suggests a high degree of overlap (Figure 3C-D); otherwise, the data should be represented differently. Though it would be technically challenging, the authors should normalize their intensity of expression to a ubiquitously expressed protein that does not change during activation as normalization is unclear. If Myogenin is expressed as intensely as is Pax7, it suggests an in vitro artefact or a staining issue. Figure 3D should also be revised to show comparable images of each motility state. Also, inclusion of motility videos with traces would be helpful.
The authors mention that pseudotemporal gene expression patterns determined by Monocle (Figure 2F) identify other non-monotonic behaviour. Which gene modules are these (Figure 2F)? To my eye, none display this pattern. Also, where is Pax7 in the heat map? What is myogenin expression like in the spline-plot (Figure 2G), as it is used as a readout in the following figure.
In the spline plots (Figure 2G), there seems to be a big gap along the psuedotime axis. For instance, between 15-30 on the psuedotime/x-axis, few cells are expressing Myf5, Pax7, or Spry1. Could it be that this gap of expression account of the pattern of non-monotonic expression for Pax7? Or could is gap be due to in vitro culturing/activation, whereas, in vivo activation would show a smooth transition (without gaps)? One suggestion would be to conduct analyses on freshly-isolated myotoxin-injured muscle stem cells or compare their data with other published datasets.
Finally, could the non-monotonic expression of Pax7 be attributed to the dynamics of LRCs? In Figure 5F LRCs make up the majority of transcriptional cluster 2 and show significantly differential expression of Pax7.
Therefore, the increase of Pax7 in the spline plots (Figure 2G) could simply be explained by LRCs dominating that cluster. Do LRCs predominantly make up an activated muscle stem cell population in vivo? If not, this suggests that the in vitro culturing biases for LRCs and may skew the authors' interpretations.

Concern 2: Figures and accompanying text need to be carefully proofread and edited to be more rational. Some of the figures are missing scale bars (Figure 2F, Figure S1A). Accompanying text refers to supplemental figures as the whole figure; while, it would be better to point the reader to the specific data mentioned to support in-text claims. Figures are also mentioned in random order,

© 2020. Published by The Company of Biologists under the terms of the Creative Commons Attribution License (https://creativecommons.org/licenses/by/4.0/).

for example: reference to Figure 2G before Figure 2F (page 7), no reference/mention of Figure 4E, as well as figure legends are mentioned in random order (Figure S4).
The labelling of clusters should also be more logical. One issue is that from figure to figure, the clustering is re-drawn (as expected, as it is a different assay) yet the same cluster # is used and colour scheme and sometimes can be confusing. This can be amended by changing the colour schemes or remaining them.
Likewise, in Figure 2E, it would be more logical to have clusters numbers ascend accordingly; whereas it is currently 3 -> 0 -> 1 -> 2 (from quiescence to activation).Similarly, in Figure 3C, the most differentiated cell type would be highest for Myogenin intensity, so logically speaking, it would be furthest right on the x-axis and re-labelled as state #4 as being least motile. Again, numbers should be edited. Reference to Fig S3 is supposed to be Fig S2 (page 7). In the legend for Figure 2C, the authors mention the overlay of MRFs but only 1 MRF is displayed (Myod), while Pax7 and Sprouty are not MRFs. Reference #13 and #14 are the same. Some of the in figure text is not interpretable (for example Figure 1F, the motility features are not described and are coded), Figure 4B heat map text is too small to read.
Though these issues individually are not glaring per se, altogether, they are detracting to the reader and the authors' data. The authors should also carefully check for other issues, as there are likely more edits beyond the ones highlighted here.

Concern 3: Lineage regression by psuedotime analyses and differences in aged contexts The authors state that lineage regression occurs robustly in both young and aged satellite cells, but do LRCs and non-LRCs differ? Further in the discussion, they also seem to contradict themselves by saying that there are no bi-stable states. If cells are regressing, would that not indicate that they are bi-stable? Perhaps this "basin of attraction" or missing primed state cannot be captured in vitro. Moreover, Galert cells can revert back to a QSC state (Rodgers et al., 2014) as well as the existence of reserve and self-renewing muscle stem cells would suggest dynamisms and not a simple path down of activation.
Is there significant differential expression of cell cycle genes in LRCs compared to non-LRCs, especially if LRCs comprise of the more of the activated cluster? Are the genes displayed in Figure 5B significant? And what is the significance cutoff? Was if 0.15 log2 (page 7)? If so, why was such a lowcut off chosen?
Aging largely did not change the transcriptional profiles, but rather their rate of activation. However, studies have shown transcriptional and functional differences (reviewed in Blau et al., 2015); thus, suggesting there is a difference in their final state. The authors should amend their claims or resolve these confounding results by addressing it in the discussion.

Reviewer 2

Advance summary and potential significance to field

By combining time lapse imaging and scRNA-seq, Kimmel and coworkers provide a comprehensive and detailed analysis of how young, aged, label-retaining (LRCs), and label-non retaining (nonLRCs) muscle stem cells (MuSCs) are activated in culture. The datasets presented in the manuscript complement and significantly extend existing scRNA-seq datasets providing a rich source of information that will greatly benefit the scientific community. As such, the manuscript is an ideal candidate for publication in Development.

Comments for the author

Clarifications of some points will help the readership.

COMMENTS:

1. Young and aged MuSCs are observed to have distinct activation with aged MuSCs displaying delayed kinetics (Figure 1). This observation may suggest that, given sufficient time, aged MuSCs may catch up with young MuSCs. Have the authors tried to culture MuSCs for longer times (>48 hrs) to see if this is the case? Do young MuSCs always retain a temporal advantage over old MuSCs? The

© 2020. Published by The Company of Biologists under the terms of the Creative Commons Attribution License (https://creativecommons.org/licenses/by/4.0/).

inability of older mice to repair injured muscles, even left to recover for long time, would suggest that this is the case.

2. Figure 2. The authors report the interesting observation that Pax7 is expressed in activated MuSCs. What are the UMIs for Pax7 in quiescent (cluster 3, Figure 2D) and activated (cluster 3 Figure 2D) clusters? Which other genes (myogenic and non-myogenic) are co-expressed in the Pax7+ cells within cluster 2? Also, of the total profiled, how many cells within quiescent and activated clusters (Figure 2C) express Pax7 and/or Spry1?

3. Figure 4E. What do the values represented x- and y- axis represent?
In Figure 4D legend, shouldn't it be "Gene-wise AUROC analysis demonstrates that single gene is NOT predictive of MuSC age state"?

4. Throughout the manuscript genes are reported to be differentially expressed in different cell states (quiescence and activation) and in young and aged MuSCs. For instance, expression of 2,631 is increased and that of 1,034 decreased during activation (page 7). 174 differentially expressed genes between aged and young MuSCs (page 10, Figure 4B). 200 differentially expressed genes in quiescent aged and young MuSCs (page 10). 275 differentially expressed genes in activated aged and young MuSCs (page 10) and in many other instances (Figure 4H, Figure 5D, Figure 5E,G). It would be very helpful if the authors could develop Tables in form of Excel spreadsheet where all these genes and the different cell conditions are indicated.

5. Figure 6C. For the readership less familiar with scRNA-seq analysis, can the authors elaborate on how the shape of RNA velocity curve suggests a switch-like process for myogenic activation?

6. My understanding is the reserve cells fail to enter differentiation. What's the evidence that MuSCs transition "backwards" rather than failing going "forward"? Can the authors indicate which genes are differentially modulated in the ~ 16% of young MuSCs regressing in pseudotime (Figure 7) and correlate these genes with those expressed in the different clusters reported in Figure 2?

7. Figure 6E. The authors may want to elaborate on young and aged MuSC curves crossing-over after 1000 timestep.

8. Figure S4. There is no H panel.

Reviewer 3

Advance summary and potential significance to field

The Kimmel et al submission seeks to apply a combination of single cell behaviour tracking scRNAseq, and machine learning to untangle secrets in the functional discrepancies observed when comparing young to aged mouse muscle stem cells. To my knowledge, no others researchers in the muscle stem cell field have applied this type of combinatorial approach to the study of muscle stem cells, and certainly not in the context of aging, which provides elements of novelty and timeliness.

Comments for the author

Major Issues
In spite of potential enthusiasm, unfortunately the manuscript in its current state is almost entirely descriptive and speculative, while, in the opinion of this reviewer, not offering enough experimental evidence to support claims made in the study. And those claims that are supported by data (related to single cell tracking) support rather than substantially extending work done by others characterizing young and aged muscle stem cell behaviors using time-lapse based clonal studies (examples include: Gilbert, Science, 2010; Cosgrove Nat Med, 2014, and others).

It should also be noted that the manuscript is rather overwhelming to read as it is quite dense and moves between a number of intellectually stimulating, though rather distinct topics and queries, with little depth, but at a substantial speed, and with quite some length.

© 2020. Published by The Company of Biologists under the terms of the Creative Commons Attribution License (https://creativecommons.org/licenses/by/4.0/).

The work may be of importance to the community, but in its current state, it is difficult to pinpoint one or two of the speculative statements as ‘key’ and to offer a series of wet lab experiments that would bring it up to a level appropriate for a Development reader.
Ultimately the work requires focus and should be split into at least two smaller stories that are interrogated in more depth.

Minor Issue
Be sure to edit Figures and Figure Legends for typos.
The abstract should be rewritten to highlight novel aspects of the study. It is not accurate to state that ' it remains unclear if the transition states and rates of activation are uniform across cells, or how features of this process may change with age' in light of single cell studies by others that clearly show the time to first division is quite varied for young muscle stem cells (Gilbert, 2010) and that with age, there are populations of cells that failed to enter cell cycle and produce a colony in the same time frames observed for young cells (Cosgrove, 2014). The current work is showing something a bit different than what they have stated and it is important to make this clear for the reader.

First revision

<u>Author response to reviewers' comments</u>

Response to Reviews: [DEVELOP/2019/183855] – Aging induces aberrant state transition kinetics in murine muscle stem cells

We thank the reviewers for providing helpful feedback that has greatly improved the manuscript. We have performed several additional analyses in response to reviewer insights, and we have likewise modified the main text to clarify our intentions and moderate claims based on reviewer feedback.

We have outlined responses to specific reviewer comments below, with reviewer feedback quoted in gray boxes.

Reviewer 1 Comments

> One of bigger issues is the use of in vitro conditions to trigger an activation state. While this may be necessary for their experimental design, it can also hamper the interpretation of results and their conclusions. Further details and suggestions are stated below.

We agree with the reviewer that the *in vitro* activation assay we use may not perfectly reflect the *in vivo* behavior of muscle stem cells. However, we believe that *in vitro* systems provide useful, if incomplete models of cell biology, as evidenced by the long history of discovery through *in vitro* modeling in the myogenesis field.

In the context of our study, the *in vitro* model we employ allows us to ensure that all cells experience uniform stimulus, unlike the heterogeneous stimulus introduced by an *in vivo* injury model. Likewise, this model provides us with a platform we can interrogate using timelapse imaging approaches to complement our molecular work. Similar cell behavior studies are unfortunately intractable in the *in vivo* context [Siegel et al., 2009, Gilbert et al., 2010, Cosgrove et al., 2014, Blau et al., 2015].

In order to ensure that our readers understand the limitations of our model, we have added text to the Results and Discussion to highlight this point. We have inserted a copy of these additions below.

© 2020. Published by The Company of Biologists under the terms of the Creative Commons Attribution License (https://creativecommons.org/licenses/by/4.0/).

Results:

Our *in vitro* activation assay may not fully capture *in vivo* stem cell activation dynamics. However, our *in vitro* assay allows for a homogeneous activation stimulus across all cells, unlike *in vivo* injury models and previous work demonstrates that *in vitro* MuSC activation recapitulates many aspects of *in vivo* activation.

Discussion:

The *in vitro* model we employ allows us to assess cell-instrinsic differences between young and aged MuSCs with a homogenous activation stimulus, but may not fully recapitulate the *in vivo* biology. Future work may explore stem cell activation *in vivo* to determine if cell-extrinsic changes with age exacerbate or dampen the differences in activation we observe between young and aged MuSCs. *In vivo* imaging technologies for MuSCs are nascent [Webster et al., 2016], but technological advances may allow for similar quantitative cell behavior analysis *in vivo* in the future.

A few concerns are raised, since other single cell analyses show the opposite: Pax7 decreases upon activation. In the Dell'Orso et al. (2019) study, they found 2 groups of MuSCs: one being Pax7Hi and MyodLow and the other being Pax7Low and MyodHigh. In another study by De Micheli et al. (2019, bioRxiv), Pax7 also drastically drops upon activation (Figure 3D). These two studies seem to conflict the findings presented here. Could the "activation" state that the authors present here be merely an in vitro artefact?

We agree that when averaging across all cells at a given timepoint, *Pax7* decreases with activation. Classic studies of single cell myogenic transcription (DD Cornellison et. al., 1999) have demonstrated this point beautifully.

Similar to Dell'Orso 2019 and De Micheli 2019, we find that *Pax7* levels are lower in our 18 hour time point relative to our 0 hour timepoint. We have presented this point more clearly in a new supplemental figure panel showing *Pax7* expression within each transcriptional cluster.

© 2020. Published by The Company of Biologists under the terms of the Creative Commons Attribution License (https://creativecommons.org/licenses/by/4.0/).

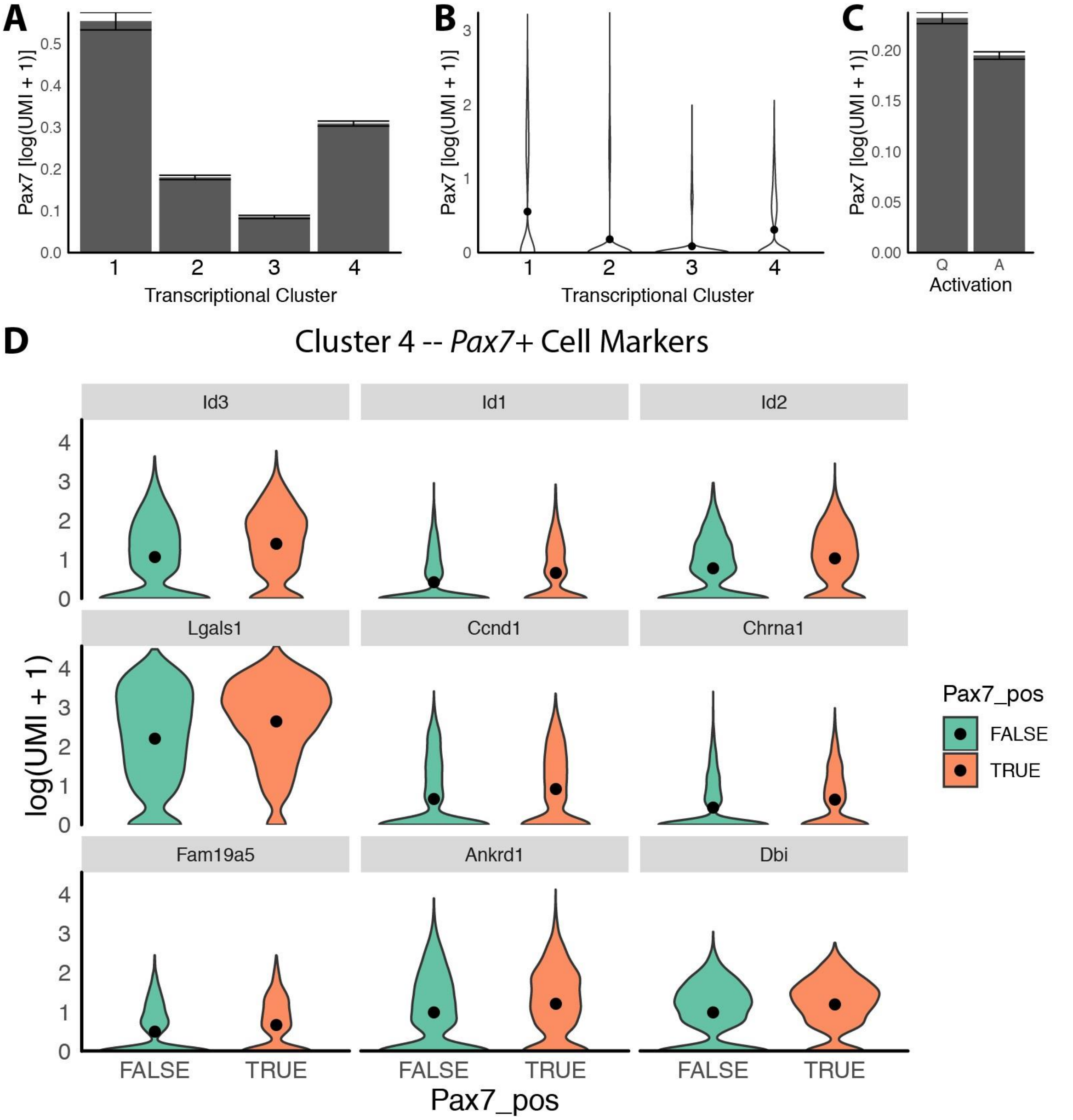

Supp. Fig. S3: ***Pax7* is lower in activated MuSCs than freshly-isolated MuSCs.** (**A**) Expression of *Pax7* in each transcriptional cluster, ordered by pseudotime. *Pax7* expression decreases in early activation (Cluster 3, 0, 1), then increases slightly in the final activation cluster (2). **(B)** Data from **(A)**, presented as violin plots. **(C)** Expression of *Pax7* in quiescent, freshly-isolated MuSCs and activated MuSCs after 18 hours *in vitro*. Considering all cells at each timepoint, *Pax7* expression is decreased in activated cells. **(D)** Significant differentially expressed genes within the *Pax7*+ population of the activated Transcriptional Cluster 4 (q < 0.05, Wilcoxon Rank Sums). We found inhibitors of myogenic differentiation *Id1, 2, 3* and cell cycle gene *Ccnd1* were enriched in *Pax7*+ cells.

We see that in our data, both clusters in the activated timepoint have lower *Pax7* than the clusters in the quiescent time point, similar to results in Dell'Orso 2019, De Micheli 2019. Likewise, we find that one cluster at the quiescent time point has higher *Pax7* than the other. However, examining expression of *Pax7* in the clusters from our 18 hour time point, we find that the more activated cluster actually displays higher *Pax7* than the less activated cluster at the 18 hour timepoint. In a coarse-grained way, this analysis recapitulates the result we found through pseudotime – *Pax7* decreases with activation, but the decreases is not simply monotonic, rather some later stages of activation display higher

© 2020. Published by The Company of Biologists under the terms of the Creative Commons Attribution License (https://creativecommons.org/licenses/by/4.0/).

expression levels than some earlier stages.

The time points in our study (0, 18 hours) are much closer together than those in Dell'Orso 2019 (0, 60 hours) or De Micheli 2019 (0, 120, 168 hours). If the slight increase in *Pax7* we observe (occurring at 18 and 36 hours in our scRNA-seq and imaging assays respectively) is transient, this phenomenology may not be observed in these previous data sets due to the difference in time points. We believe this difference in timing may account of the lack of non-monotonic behavior in the pseudotemporal analysis of De Micheli 2019. Because the earliest activated timepoint in that study is at 120 hours, a transient increase in *Pax7* may have already passed and cells at the region of the pseudotemporal curve where this occurs may be poorly populated.

To address this discrepancy, we have amended the Results of our manuscript in the relevant section to discuss this point. A section of the additions are included below:

Analysis of the mean expression in each cluster confirms non-monotonic changes in *Pax7* with activation (Fig. S3B, C). Our data are consistent with previous reports of decreased *Pax7* with activation at the ensemble level [Cornelison and Wold, 1997, Dell'Orso et al., 2019, De Micheli et al., 2019] when we consider only the mean expression of *Pax7* at the quiescent and activated timepoints
(Fig. S3A).
If Myogenin is expressed as intensely as is Pax7, it suggests an in vitro artefact or a staining issue.

> Similarly, Pax7+/MyoG+ cells are rarely observed in floating ex vivo cultured myofiber assays (Zam- mit et al., 2004) and the dogma suggests that MyoG+ cells are Pax7- (Yin et al., 2013). The data in Figure 3 suggests a high degree of overlap (Figure 3C-D); otherwise, the data should be represented differently.

In our fluorescent microscopy setup, we measure Pax7 and MyoG using separate fluorescent channels. For this reason, the intensity of fluorescence from each protein is not directly comparable. For example, the exposure times of the relevant channels are independent of one another, such that the absolute intensity of each fluorophore is arbitrary. We therefore focus only on the relative changes of each protein across the clusters in our cell behavior assay.

We agree that the normalization scheme as presented is confusing and thank the reviewer for pointing this out. We normalized the data such that the intensity of each protein in first cluster was equal to 1 to highlight the dynamics of expression. However, this leads to a false sense that Pax7/MyoG levels are of similar intensity.

We have amended the main text figure to show the raw Pax7 intensities, as well as a binarized version of these intensities as is common in the field. We have also provided a panel showing that Pax7+/MyoG+ cells are *very rare* in our data, as expected from the literature the reviewer cites. We have copied the amended main text figure and new supplemental figure below.

© 2020. Published by The Company of Biologists under the terms of the Creative Commons Attribution License (https://creativecommons.org/licenses/by/4.0/).

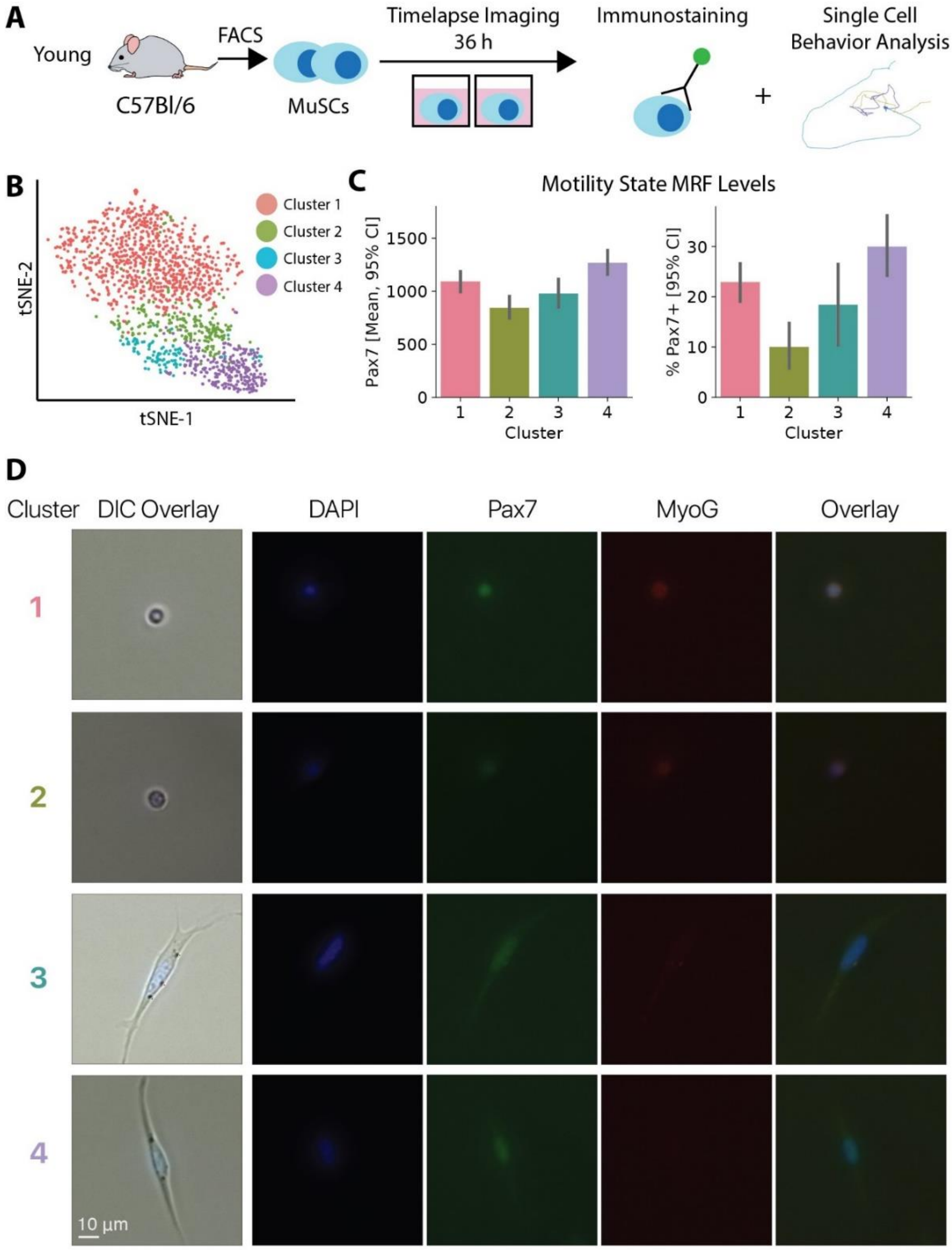

Fig. 3: **Pax7 is non-monotonically regulated across MuSC cell behavior states during activation.** **(A)** Experimental design schematic. MuSCs were isolated, timelapse imaged in culture for 36 hours, and subsequently immunostained. Behavior traces and immunostaining results were matched for each cell by image registration. **(B)** t-SNE visualization of cell behavior states in motility state space, as defined by hierarchical clustering. Behavior state space was generated analyzing 12 hours of tracking data, from 24 hours after isolation to 36 hours. n = 1, 003 cells. **(C)** Pax7 immunostaining intensity (cell median) and binary frequency within each cell behavior cluster. Both quantification schemes show a non-monotonic relationship between behavioral activation state and Pax7 intensity. **(D)** Representative images of Pax7/MyoG staining in MuSCs after timelapse imaging for behavior analysis. Panels on the far left are the final DIC image from the timelapse, registered and overlaid with fluorescent immunostains. Remaining panels are raw images prior to registration.

© 2020. Published by The Company of Biologists under the terms of the Creative Commons Attribution License (https://creativecommons.org/licenses/by/4.0/).

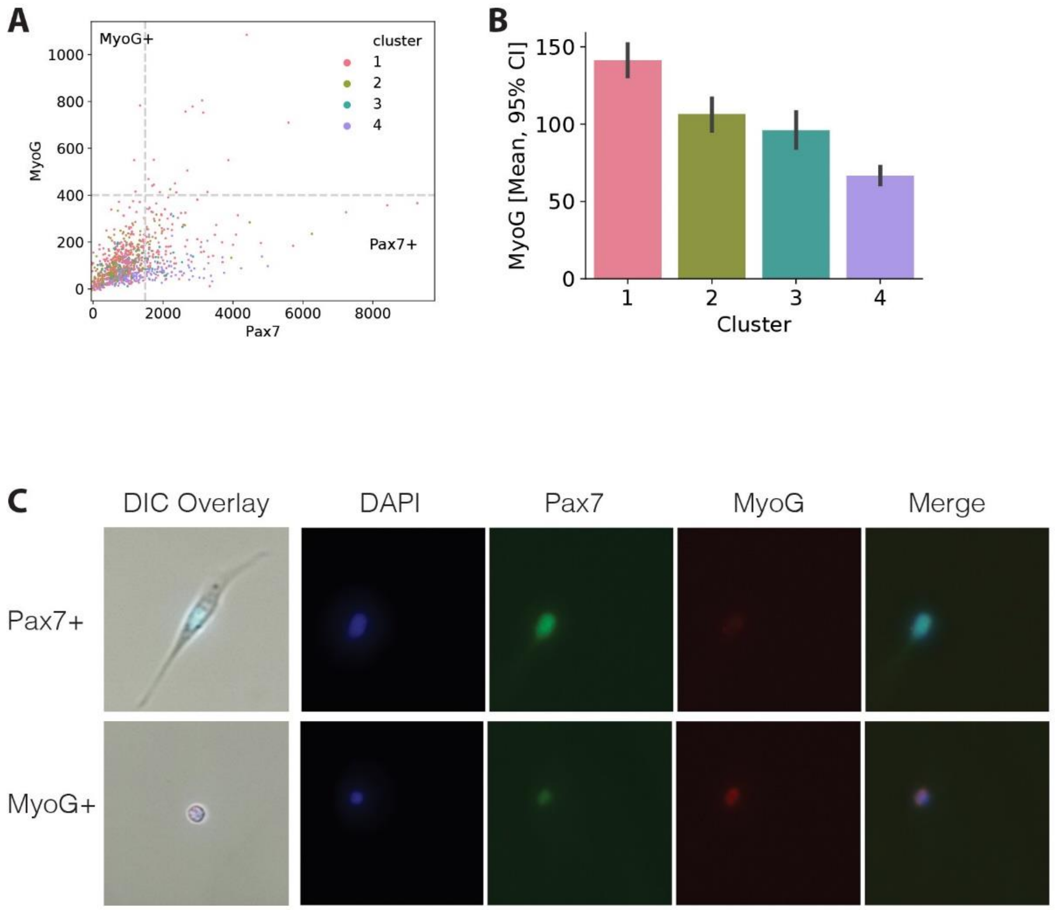

Supp. Fig. S5: **MuSC behavior states are associated with myogenic transcription factor levels.** **(A)** Pax7 and MyoG intensities for cells with a paired cell behavior recording. Intensities are presented as the median pixel intensity inside the cell nucleus. Pax7 and MyoG protein are rarely co-expressed, as expected. **(B)** MyoG protein intensity (cell median) in each cell behavior cluster. MyoG levels decrease slighly with activation, though all clusters show low expression. **(C)** Representative images of Pax7/MyoG staining in cells after timelapse imaging. Panels on the far left are the final DIC image from the timelapse, registered and overlaid with fluorescent immunostains. Remaining panels are fluorescence images prior to registration. Fluorescence images are equitably rescaled across cells within each channel for presentation.

> Figure 3D should also be revised to show comparable images of each motility state. Also, inclusion of motility videos with traces would be helpful.

We agree that inclusion of staining images for each cluster would be helpful for readers. We have amended Figure 3D to include panels showing images of each motility state (inserted above). We have also included cell motility videos as supplemental material.

> The authors mention that pseudotemporal gene expression patterns determined by Monocle (Figure 2F) identify other non-monotonic behaviour. Which gene modules are these (Figure 2F)? To my eye, none display this pattern

We thank the reviewer for pointing out this oversight. We have added text to the Results to highlight modules which display non-monotonic behavior (Module 1, 3).

© 2020. Published by The Company of Biologists under the terms of the Creative Commons Attribution License (https://creativecommons.org/licenses/by/4.0/).

Genes with non-monotonic behavior are evident in both modules where the highest expression level (orange-red) is located near the center of the pseudotime trajectory, rather than at either end. By contrast, Module 2 shows monotonically decreasing genes, and Module 4 shows monotonically increasing genes.

We have amended the results to clarify these points. A sample of the additions are copied below

Results:

For example, many genes in Modules 1 and 3 display maximum expression at points in between the most quiescent and most activated states. A peak of expression at an intermediary point indicates that gene expression in these modules is non-monotonic during activation, first increasing then decreasing as cells transition through the activation trajectory. Module 1 contains genes related to mRNA processing and splicing, as determined by gene ontology analysis (see Methods). Module 3 contains genes related to cell cycle regulation and developmental processes (Fig. S3). By contrast, most genes in Module 2 decrease monotonically across pseudotime, and most genes in Module 4 monotonically increase across pseudotime.

Also, where is Pax7 in the heat map?

We have highlighted the location of *Pax7* in the pseudotemporal heatmap, along with *Myod1* and *Myf5*.

What is myogenin expression like in the spline-plot (Figure 2G), as it is used as a readout in the following figure.

We have included a spline-plot showing *Myog* expression in the supplemental material. At our 18 hour timepoint where we performed single cell RNA-seq, we detect very little *Myog* expression, as expected from previous work [Cornelison and Wold, 1997]. In the entire 20,000+ cell dataset, only 22 cells have a detectable level of *Myog* transcript. The pseudotemporal curve inferred for *Myog* expression is therefore based on very little data and may not reflect the true expression dynamics. We have embedded this new supplemental figure panel below for convenience.

We note that the following figure (Figure 3) is taken at a later time point (36 hours) than our single cell RNA- seq data (18 hours) and therefore we expect the abundance of MyoG to be higher in the behavior experiment than the abundance of *Myog* in the scRNA-seq experiment. We observe that both are nonetheless low.

© 2020. Published by The Company of Biologists under the terms of the Creative Commons Attribution License (https://creativecommons.org/licenses/by/4.0/).

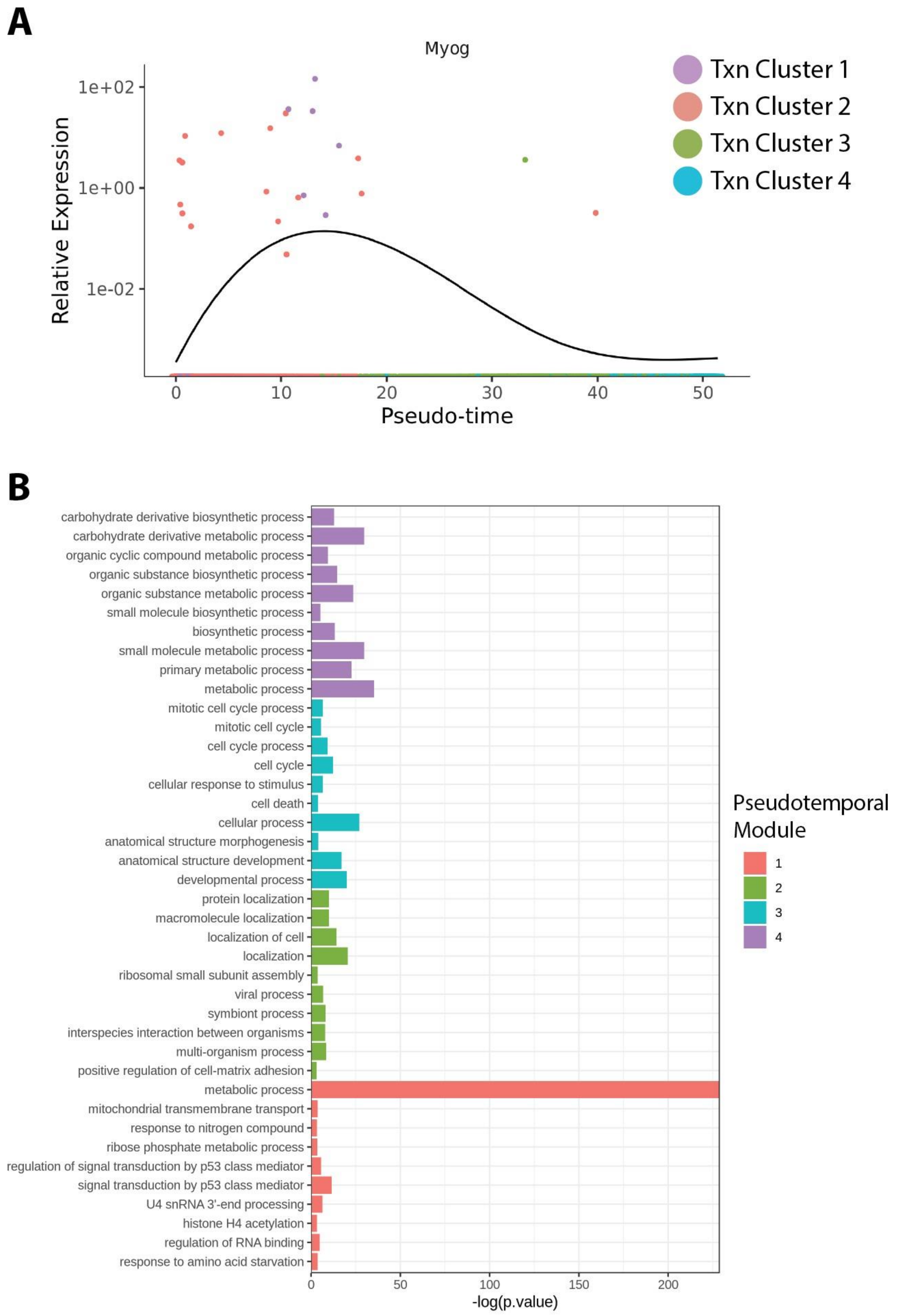

Supp. Fig. S4: **Pseudotemporal analysis of myogenic activation reveals non-monotonic regulation. (A)** Spline fit of *Myog* expression over pseudotime. Only 25 cells in the experiment express *Myog* at detectable levels. **(B)** Gene ontology enrichment analysis for Pseudotemporal Modules, suggesting coherent groups of co-regulated genes.

© 2020. Published by The Company of Biologists under the terms of the Creative Commons Attribution License (https://creativecommons.org/licenses/by/4.0/).

In the spline plots (Figure 2G), there seems to be a big gap along the psuedotime axis. For instance, between 15-30 on the psuedotime/x-axis, few cells are expressing Myf5, Pax7, or Spry1. Could it be that this gap of expression account of the pattern of non-monotonic expression for Pax7? Or could is gap be due to in vitro culturing/activation, whereas, in vivo activation would show a smooth transition (without gaps)?

We thank the reviewer for highlighting this point. The "gap" in our pseudotemporal trajectory represents regions of state space where we do not collect cells. This indicates that there are states of activation between those we observe at 0 hours and 18 hours. As such, this is an effect of our sampling times rather than the *in vitro* nature of our assay. Similar gaps in pseudotemporal trajectories are present in many studies where the sampling times are not dense enough to capture all intermediary states (Dell'Orso 2019, De Michelli 2019).

We have amended the Results to reflect this point.

Results:

The discontinuity between the freshly-isolated and activated time points suggests that there are intermediary activation states we do not capture.

Finally, could the non-monotonic expression of Pax7 be attributed to the dynamics of LRCs? In Figure 5F, LRCs make up the majority of transcriptional cluster 2 and show significantly differential expression of Pax7. Therefore, the increase of Pax7 in the spline plots (Figure 2G) could simply be explained by LRCs dominating that cluster. Do LRCs predominantly make up an activated muscle stem cell population in vivo? If not, this suggests that the in vitro culturing biases for LRCs and may skew the authors' interpretations.

We agree that LRC/nonLRC dynamics may contribute to the non-monotonic Pax7 expression we observe. We believe LRCs are activating faster than nonLRC based on multiple lines of evidence (scRNA-seq, EdU staining). An intrinsically higher level of *Pax7* in LRCs may account for a higher level of *Pax7* in the most activated cells which are disproportionately LRCs. We also know that LRCs provide a greater contribution to muscle repair after transplantation [Chakkalakal et al., 2012, Chakkalakal et al., 2014], consistent with improved regeneration associated with a rapid activation response [Rodgers et al., 2014, Rodgers et al., 2017, Scaramozza et al., 2019]. We have amended the Results to discuss this possibility.

If this is the case, it would represent a scenario where heterogeneous MuSC subpopulation dynamics give rise to non-monotonic gene expression behavior at the ensemble level. Regardless of which model is correct (non-monotonic behavior in every cell, or LRCs "racing ahead" of nonLRCs), we believe this phenomenon suggests the current model of lower Pax7 in more activated cells is incomplete.

A sample of our amendments to the Results is pasted below.

Results:

The enrichment of LRCs in Transcriptional Cluster 4 may also underlie the non-monotonic behavior of *Pax7* across pseudotime (Fig. 2G). LRCs express higher levels of *Pax7* [Chakkalakal et al., 2012], such that rapid activation of LRCs may give rise to a population structure that present non- monotonic changes in *Pax7* when averaging across all cells at a given activation state.

© 2020. Published by The Company of Biologists under the terms of the Creative Commons Attribution License (https://creativecommons.org/licenses/by/4.0/).

Figures and accompanying text need to be carefully proofread and edited to be more rational. Some of the figures are missing scale bars (Figure 2F, Figure S1A). Accompanying text refers to supplemental figures as the whole figure; while, it would be better to point the reader to the specific data mentioned to support in-text claims. Figures are also mentioned in random order, for example: reference to Figure 2G before Figure 2F (page 7), no reference/mention of Figure 4E, as well as figure legends are mentioned in random order (Figure S4).

We thank the reviewer for taking the time to provide such detailed feedback. We have amended the references to Supp. Figures to include specific subpanels, as well as reorganized the main and supplemental figures to improve clarity. We have likewise changed panel names to reflect the order in which data are mentioned in the text wherever possible.

The labelling of clusters should also be more logical. One issue is that from figure to figure, the clustering is re-drawn (as expected, as it is a different assay) yet the same cluster number is used and colour scheme and sometimes can be confusing. This can be amended by changing the colour schemes or remaining them.

We agree with the reviewer that this is confusing. We've changed the cluster names such that higher numbers always indicate more activated clusters. We've also prepended assay level descriptors (e.g. “Behavior Cluster 1”, “Transcriptional Cluster 1”) to differentiate clusters in the different assays.

Some of the in figure text is not interpretable (for example Figure 1F, the motility features are not described and are coded), Figure 4B heat map text is too small to read.

We thank the reviewer for highlighting these issues. We have amended the font sizes to ensure all in figure text is readable. In particular, we have increased the font size for heatmap labels and provided descriptive names for motility features. We have amended the motility features panel in Fig. 1 to use semantic names. We have also added descriptions of our motility features in the Supplemental Methods. This description is copied below for convenience:

We used a set of 57 motility features from *Heteromotility*, excluding features that focus on turning angles. Features displayed in Fig. 1 include: “Total Distance,” a measure of the total distance a cell traveled; “Net Distance,” a measure of the net distance a cell traveled; “Linearity,” or the r^2 value of a linear regression through cell positions, “Rank Correlation,” as computed using a Spearman coefficient; “Progressivity,” computed as the ratio of net distance to total distance; “Mean Speed,” computed as the total distance over time; “Mean Squared Displacement,” a metric of the distance moved by a cell relative to the starting location; “Hurst Coefficient,” a description of the self-similarity between cell behaviors when modeled using a fractional Brownian motion process; “Non-Gaussian Coefficient,” measuring the degree to which cell displacements do not follow a Gaussian distribution; “Kurtosis $\tau = 1$” the kurtosis of the displacement distribution with a time lag of 1; “Kurtosis $\tau = 5$” the kurtosis of the displacement distribution with a time lag of 5; “Mean Moving Speed,” the mean speed of a cell computed only during times the cell is not stationary; “Average Time Moving,” the proportion of time a cell spends moving; and “Autocorrelation,” a measure of self-similarity within the displacement time series.

© 2020. Published by The Company of Biologists under the terms of the Creative Commons Attribution License (https://creativecommons.org/licenses/by/4.0/).

In the legend for Figure 2C, the authors mention the overlay of MRFs but only 1 MRF is displayed (Myod), while Pax7 and Sprouty are not MRFs. Reference 13 and 14 are the same.

We thank the reviewer for pointing out this oversight. We meant to indicate "myogenic regulatory genes," whereas MRFs obviously do not include *Pax7, Spry1*. This has been corrected in the text and figure legends.

The authors state that lineage regression occurs robustly in both young and aged satellite cells, but do LRCs and non-LRCs differ?

This is a fascinating question. To address this possibility, we have performed lineage regression analysis in LRCs and nonLRCs from young and aged animals in our study. We find that both LRCs and nonLRCs exhibit "lineage regression" in that some cells of each population appear to be moving backward in pseudotime. In young animals, nonLRCs appear to move "backward" more often than LRCs in later portions of the activation trajectory. Quantitatively, roughly 25% of young nonLRCs are moving backward while only 12% of young LRCs move backward (x^2 test $p < 0.0001$). In aged animals, LRCs and nonLRCs similarly exhibit differences in lineage regression frequency (aged LRC 12%, aged nonLRC 21%; x^2 test $p < 0.0001$). These data suggest that the more rapid activation we observe in LRCs may in part be due to a lower frequency of lineage regression during the activation process.

We have summarized these findings in additions to the supplementary material (copied below for convenience). We have also amended the Results and Discussion to address this interesting point.

Results:
This regression behavior appears robust to age-related changes (Fig. S13A). Comparing LRCs to nonLRCs, LRCs regress less frequently than nonLRCs in young and aged animals (Fig. S13B, C; x^2 test, $p < 0.0001$). This decreased frequency of lineage regression may contribute to the more rapid activation of LRCs we observe based on transcriptional profiles (Fig. 5) and EdU incorporation (Fig. S10C).

Discussion:

Here, we find that MuSCs progress through the activation process stochastically, with a non-trivial proportion of the population moving "backwards" through the activation process. This suggests that the heterogeneity of MuSC positions along the activation trajectory arises as an accumulation of differences in the rate of cell state transitions. **These differences appear to be both stochastic and associated with distinctive features between MuSC subpopulations, such as proliferative history.**

Further in the discussion, they also seem to contradict themselves by saying that there are no bi-stable states. If cells are regressing, would that not indicate that they are bi-stable? Perhaps this "basin of attraction" or missing primed state cannot be captured in vitro. Moreover, Galert cells can revert back to a QSC state (Rodgers et al., 2014) as well as the existence of reserve and self-renewing muscle stem cells would suggest dynamisms and not a simple path down of activation.

We thank the reviewer for highlighting our lacking of clarity on this point. Lineage regression does not necessarily imply that a bistable state exists between the most quiescent and most activated states we observe. The regression we observe occurs a

© 2020. Published by The Company of Biologists under the terms of the Creative Commons Attribution License (https://creativecommons.org/licenses/by/4.0/).

minority of the time across the pseudotime trajectory, reminiscent of a biased random walk. We define a bistable state here as a state where stability decreases in both the forward and backward direction in a local neighborhood, such that the state forms a “basin” of stability. If such a state were present, we might expect to see a location between the most quiescent and activated state where RNA velocity vectors point inward from positions just behind and ahead of the stable point.

We do not observe such a point here, but as the reviewer suggests, it is entirely possible that such a point exists in other conditions, such as *in vivo*. We have amended the Results and Discussion to highlight this important caveat of our results. Most prominently, we have amended our Discussion of implications related to G_{alert}. The activation trajectory we observe is in the context of a strong and constant activation stimulus (*in vitro* culture, Gilbert 2010). Other conditions such as homeostasis *in vivo* may allow for bistable states to emerge between the quiescent and fully-activated MuSC state.

We have amended the Results and Discussion to address this point. A sample of the additions are copied below. We have additionally removed portions of the text that may be misleading in light of this consideration.

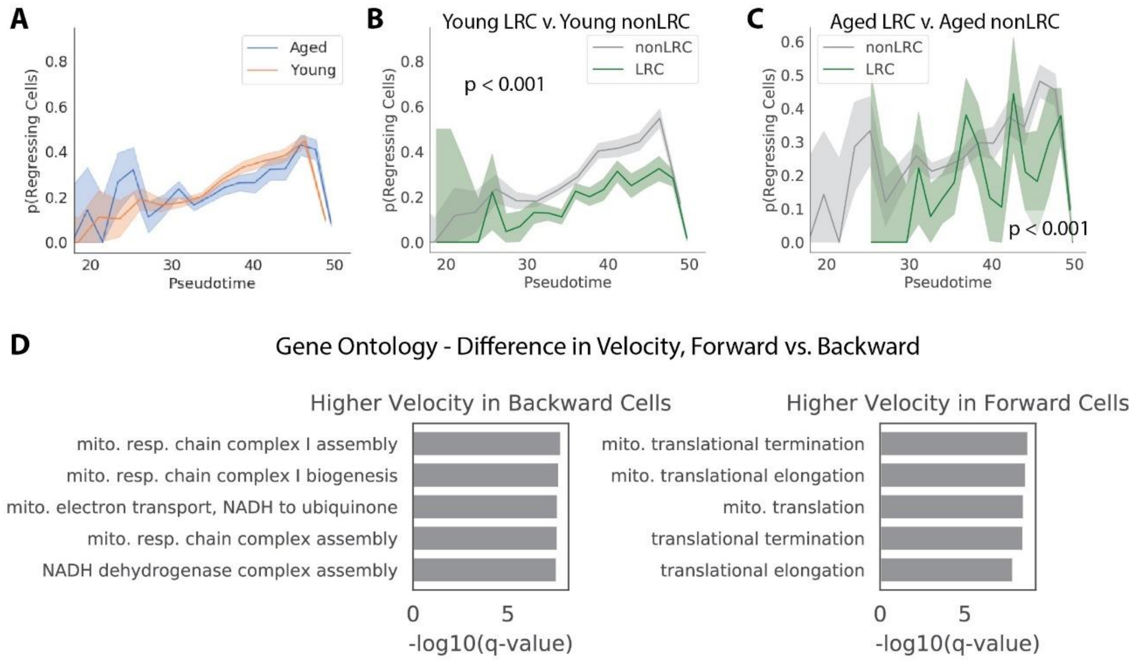

Supp. Fig. S13: **Lineage regression frequency with age (A)** The proportion of “regressing” cells across pseudotime in aged and young MuSCs, revealing little age dependence. Proportions were computed by coarse-graining pseudotime into equally sized bins. **(B)** Proportion of “regressing” cells across pseudotime in young LRCs and nonLRCs. nonLRCs regress more frequently late in the activation process (x^2 test, p < 0.0001. **(C)** The difference in lineage regression frequency between LRCs and nonLRCs is also present in MuSCs from aged animals (x^2 test, p < 0.0001). **(D)** We computed the difference in velocity for each gene between cells moving “forward” and “backward” in the activation trajectory. Gene Ontology enrichments suggest forward moving cells have higher velocity for protein translation and biogenesis programs, while backward moving cells have higher velocity for mitochondrial oxidative phosphorylation programs.

Results:

We note that the switch-like process suggested by these results may be unique to our *in vitro* culture setting, and does not necessarily reflect *in vivo* activation kinetics.

© 2020. Published by The Company of Biologists under the terms of the Creative Commons Attribution License (https://creativecommons.org/licenses/by/4.0/).

Discussion:

The *in vitro* model we employ allows us to assess cell-instrinsic differences between young and aged MuSCs with a homogenous activation stimulus, but may not fully recapitulate the *in vivo* biology. For instance, while we do not observe bi-stable transcriptional states between the most quiescent and most activated cells in our experiments, stable intermediary activation states may exist *in vivo* [Rodgers et al., 2014].

Is there significant differential expression of cell cycle genes in LRCs compared to non-LRCs, especially if LRCs comprise of the more of the activated cluster? Are the genes displayed in Figure 5B significant? And what is the significance cutoff? Was if 0.15 log2 (page 7)? If so, why was such a lowcut off chosen?

We observe that *Ccnd1* (Cyclin D1) is significantly enriched in LRCs relative to nonLRCs (log2 FC 0.7, $q < 0.001$), consistent with more rapid cell cycle entry in LRCs. We have amended the Results and Supplementary Figures to highlight this result.

Only *Myod1* differences in activation are signficant in Fig. 5B ($q < 0.05$). We have added indicators to the figure and specified the lack of significance for other contrasts in the legend.

We used log2FC 0.15 as a fold-change cutoff. Genes that are significantly different between LRCs and nonLRCs may be meaningful, even if the fold-change is small. For instance, microRNAs typically repress most targets by < 30%, but the cumulative effects of these small fold-changes are biologically meaningful [Ma et al., 2018, Lim et al., 2005]. For this reason, we prioritize that statistical significance of gene expression changes when reporting results, rather than selecting a large arbitrary effect size at which to threshold our determination of importance.

Aging largely did not change the transcriptional profiles, but rather their rate of activation. However, studies have shown transcriptional and functional differences (reviewed in Blau et al., 2015); thus, suggesting there is a difference in their final state. The authors should amend their claims or resolve these confounding results by addressing it in the discussion.

We appreciate the reviewer raising this important point. Similar to previous studies, we observe some transcriptional differences between young and aged cells. However, we find that these differences do not alter the overall trajectory of activation in transcriptional space, as recovered in low-dimensional embeddings, unsupervised clustering, and using pseudotime analysis. These results are consistent with previous findings of modest transcriptional differences between young and aged MuSCs during early activation [Cosgrove et al., 2014]. These results show that while transcriptional differences are present across ages, MuSCs of both ages share the same set of transcriptional states during activation, rather than each age exhibiting a distinct activation trajectory. Further, these results show that differences between ages - which exist even for cells in similar states of activation - are a much smaller source of variation than differences between a shared set of activation states.

We have amended our Discussion to clarify that our results are consistent with the existence of transcriptional differences between young and aged cells and serve to contextualize these results in light of transcriptome wide measurements. Only given our measurements of the whole transcriptome can we appreciate that age-related transcriptional changes within a given state are much smaller than changes between

© 2020. Published by The Company of Biologists under the terms of the Creative Commons Attribution License (https://creativecommons.org/licenses/by/4.0/).

states, and differences in the rates of progression between states.

A sample of our amendments is outlined below:

Discussion:

We surprisingly found minor transcriptional differences between aged and young MuSCs at a given point in the activation process. These results are consistent with previous reports of subtle transcriptional differences between young and aged MuSCs [Sousa-Victor et al., 2014, Cosgrove et al., 2014, Keyes et al., 2016]. While transcriptional differences with age have been observed previously, it remained unknown if these differences were a large enough source of variation to alter the trajectory of myogenic activation. Our single cell RNA-seq data allows us to observe that age-related changes are much smaller than changes between states of activation, such that the sequence of cellular states – i.e. the trajectory of activation – is preserved with aging.
...
These differences in activation rate suggest that some transcriptional differences between young and aged MuSCs observed at the ensemble level may be the result of differences in the distribution of cells across the activation trajectory.

Reviewer 2 Comments

1. Young and aged MuSCs are observed to have distinct activation with aged MuSCs displaying delayed kinetics (Figure 1). This observation may suggest that, given sufficient time, aged MuSCs may catch up with young MuSCs. Have the authors tried to culture MuSCs for longer times (¿48 hrs) to see if this is the case? Do young MuSCs always retain a temporal advantage over old MuSCs? The inability of older mice to repair injured muscles, even left to recover for long time, would suggest that this is the case.

We have not performed single cell RNA-sequencing or timelapses at later timepoints. The cell tracking step of timelapse imaging makes long timelapses difficult for several technical reasons. For this reason, we could not include both early and late activation in a single timelapse experiment. We therefore focused this study on early activation.

We agree with the reviewer that the question of whether or not young cells “catch up,” is fascinating, and we hope to explore this question in future work.

2. Figure 2. The authors report the interesting observation that Pax7 is expressed in activated MuSCs. What are the UMIs for Pax7 in quiescent (cluster 3, Figure 2D) and activated (cluster 3 Figure 2D) clusters? Which other genes (myogenic and non-myogenic) are co-expressed in the Pax7+ cells within cluster 2?

Cells in our quiescent time point show an average of 0.233 UMIs per cell for *Pax7* after normalization and log scaling (0.262 UMIs per 10,000). Cells in the activated time point show an average of 0.195 UMIs per cell for *Pax7* after normalization and log scaling (0.21 UMIs per 10,000). We have presented these data and the corresponding gene expression levels by cluster in a new supplementary figure, copied below.

To determine which other genes are co-expressed with the *Pax7*+ cells in Transcriptional Cluster 4 (note: renamed, Fig. 2), we performed differential expression analysis comparing Cluster 2 *Pax7*+ cells Cluster 4 *Pax7*-cells. We identified 11 genes that were significantly upregulated in the *Pax7* positive cells, including three members of the Inhibitor of DNA binding protein family: *Id1, Id2*, and *Id3* and the cell cycle gene *Ccnd1*. Members *Id* family are known to inhibit myogenic differentiation, such that these

© 2020. Published by The Company of Biologists under the terms of the Creative Commons Attribution License (https://creativecommons.org/licenses/by/4.0/).

marker genes are consistent with the idea that *Pax7*+ cells might be less committed and more proliferative than counterparts. We have included this analysis in a new supplemental figure (copied below). We have included the full differential expression results as a new supplementary table.

Results:

Comparing *Pax7*+ cells in Transcriptional Cluster 4 to *Pax7*-cells in the same cluster, we find that *Pax7*+ cells show significant upregulation of *Id1, Id2,* and *Id3* which inhibit differentiation and cell cycle gene *Ccnd1* (Fig. S3D, $q < 0.05$, Wilcoxon Rank Sums). These results are consistent with the idea that *Pax7*+ cells may be more proliferative [Zammit et al., 2006] and less committed than *Pax7*-counterparts.

Also, of the total profiled, how many cells within quiescent and activated clusters (Figure 2C) express Pax7 and/or Spry1?

We find 4,230 cells expressing *Pax7*, and 2,174 cells expressing *Spry1*. We have included a new supplementary table (Table S2) quantifying the number of cells we detect expressing these and other genes of interest (copied below).

Table S2: **Number of cells expressing genes of interest.**

Txn Cluster	Pax7	Spry1	Myf5	Myod1	Myog	Myf6
1	511	436	586	313	6	13
2	1125	812	2279	3346	16	27
3	516	256	442	4979	1	2
4	2041	606	551	5039	2	16

3. Figure 4E. What do the values represented x- and y- axis represent? In Figure 4D legend, shouldn't it be "Gene-wise AUROC analysis demonstrates that single gene is NOT predictive of MuSC age state"?

The values on the axes are the mean expression of a gene in the population indicated by the label (Young or Aged). Each of the points represents a single gene. We have amended the axes labels and legend to clarify.

We thank the reviewer for pointing out our error in the figure legend. We have corrected the text to reflect that gene wise AUROC analysis shows that a single gene is *not* predictive of age.

4. Throughout the manuscript genes are reported to be differentially expressed in different cell states (quiescence and activation) and in young and aged MuSCs. For instance, expression of 2,631 is increased and that of 1,034 decreased during activation (page 7). 174 differentially expressed genes between aged and young MuSCs (page 10, Figure 4B). 200 differentially expressed genes in quiescent aged and young MuSCs (page 10). 275 differentially expressed genes in activated aged and young MuSCs (page 10) and in many other instances (Figure 4H, Figure 5D, Figure 5E,G). It would be very helpful if the authors could develop Tables in form of Excel spreadsheet where all these genes and the different cell conditions are indicated.

© 2020. Published by The Company of Biologists under the terms of the Creative Commons Attribution License (https://creativecommons.org/licenses/by/4.0/).

We agree that this resource would be helpful for readers. We have included tables for each of the differentially expressed gene sets as Supplemental Files.

> 5. Figure 6C. For the readership less familiar with scRNA-seq analysis, can the authors elaborate on how the shape of RNA velocity curve suggests a switch-like process for myogenic activation?

We thank the reviewer for pointing out this lack of clarity. We have amended the Results with the following text to justify this description:

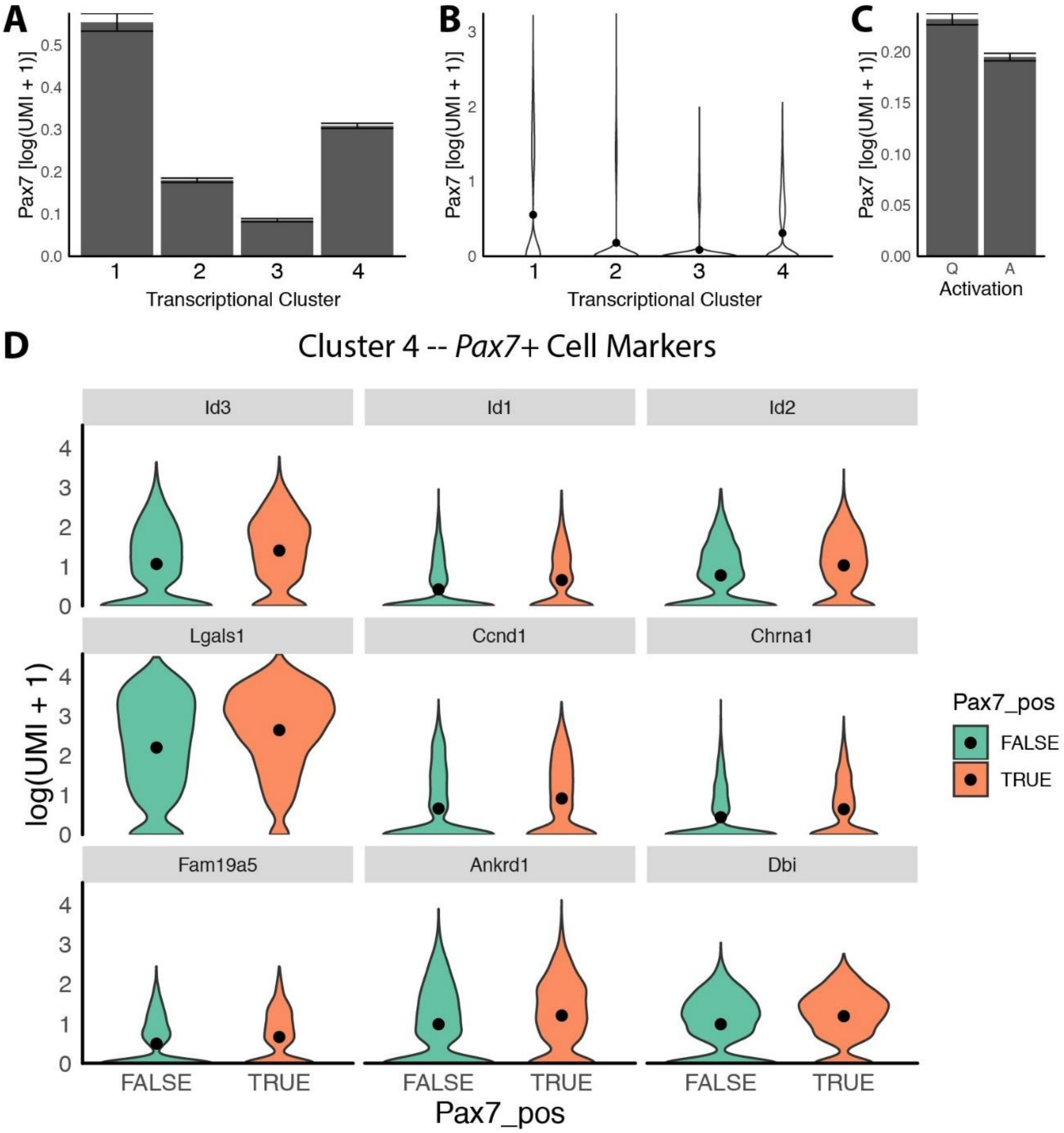

Supp. Fig. S3: ***Pax7* is lower in activated MuSCs than freshly-isolated MuSCs.** (**A**) Expression of *Pax7* in each transcriptional cluster, ordered by pseudotime. *Pax7* expression decreases in early activation (Cluster 1, 2, 3), then increases slightly in the final activation cluster (4). **(B)** Data from **(A)**, presented as violin plots. **(C)** Expression of *Pax7* in quiescent, freshly-isolated MuSCs and activated MuSCs after 18 hours *in vitro*. Considering all cells

© 2020. Published by The Company of Biologists under the terms of the Creative Commons Attribution License (https://creativecommons.org/licenses/by/4.0/).

at each timepoint, *Pax7* expression is decreased in activated cells. **(D)** Significant differentially expressed genes within the *Pax7*+ population of the activated Transcriptional Cluster 4 (q < 0.05, Wilcoxon Rank Sums). We found inhibitors of myogenic differentiation *Id1, 2, 3* and cell cycle gene *Ccnd1* were enriched in *Pax7*+ cells.

A switch-like process is characterized by one prominent period of transition, with two relatively stable states on either side. Concave transition rates are consistent with model, where the peak transition rate represents the "switch" between two states in the switch-like process. The concave transition rates we observe by RNA velocity suggests that myogenic activation is a switch-like process, corroborating our earlier observations made by cell behavior analysis [Kimmel et al., 2018].

> 6. My understanding is the reserve cells fail to enter differentiation. What's the evidence that MuSCs transition "backwards" rather than failing going "forward"?

In a fascinating report, Yoshida et. al. [Yoshida et al., 1998] describe "reserve cells" as a population of $MyoD^{Low}/Myf5^{Low}$ myogenic cells that emerge from a uniform $MyoD^{High}/Myf5^{High}$+ myoblast population upon differentiation challenge. The "rapid downregulation of MyoD" in this initial report suggests that myogenic cells have the ability to regress in the commitment program (key data in Fig. 6 and Fig. 7 of [Yoshida et al., 1998]).

Our RNA velocity data, which indicates how expression levels are changing in a single cell, indicate that many MuSCs are undergoing a transcriptional change that brings them "backward" in the lineage progression. We infer the future transcriptome of a cell as the current transcriptome plus the RNA velocity vector, then infer a pseudotime coorindate for each of these future transcriptomes. To our surprise, we found that the predicted transcriptomes for many MuSCs are earlier in pseudotime than the current state, suggesting "backward" motion. This is in contrast to the result we would expect if MuSCs were nearly "stuck" and not going "forward." In that case, we would see velocity vectors that provide positive change in pseudotime, or a change near zero, but not a negative change in pseudotime.

We note that we have no evidence cells are failing to activate in the long term, as described for reserve cells that do not differentiate even under prolonged serum starvation. Rather, we suggest that the process of activation may involve many small forwards and backwards movements in the lineage progression, rather than a smooth, continuous process. This description is consistent with our knowledge of noise in the transcriptional process [Elowitz et al., 2002, Hansen et al., 2018], such that we might expect lineage commitment to look more like the biased random walk of a gas particle crossing a room than the smooth motion of a ball rolling down a hill. To clarify this point, we have amended our Results. Samples of these amendments are included below.

Results:

RNA velocity is a measure of instantaneous change in the cell state, such that these results do not necessarily suggest a subset of cells which permanently fails to activate. Rather, these results suggest myogenic activation is a two-way process even under growth-promoting conditions, perhaps resembling a biased random walk through transcriptional space in which some steps in "reverse" occur even as the overall direction of motion progresses forward.

> Can the authors indicate which genes are differentially modulated in the 16% of young MuSCs regressing in pseudotime (Figure 7) and correlate these genes with those expressed in the different clusters reported in Figure 2?

This is a very interesting question. We have computed the difference in velocity between

© 2020. Published by The Company of Biologists under the terms of the Creative Commons Attribution License (https://creativecommons.org/licenses/by/4.0/).

forward and back-ward moving cells across all genes. Gene Ontology enrichment suggests that terms associated with protein translation and biogenesis have higher velocity in forward moving cells, while genes associated with mitochondrial oxidative phosphorylation have higher velocity in backward moving cells. These results are consistent with the idea that cells moving "forward" in activation need to activate protein translation programs, while cells that remain primitive and fail to move forward maintain the high use of oxidative phosphorylation characteristic of more primitive cells during activation (see: [Ryall et al., 2015]).

We have included the results of these analyses in a new supplemental figure panel (copied below) and table. Additions to the Results are included below.

Results:
To determine which genes underlie differences in motion through the activation trajectory, we computed the difference in velocity between forward and backward moving cells. This analysis reveals that forward moving have higher velocity for protein translation and biogenesis genes, while backward moving cells have higher velocity for mitochondrial respiration genes (Fig. S13D, Gene Ontology enrichments). These results are consistent with upregulation of protein synthesis and downregulation of oxidative phosphorylation during myogenic activation [Ryall et al., 2015].

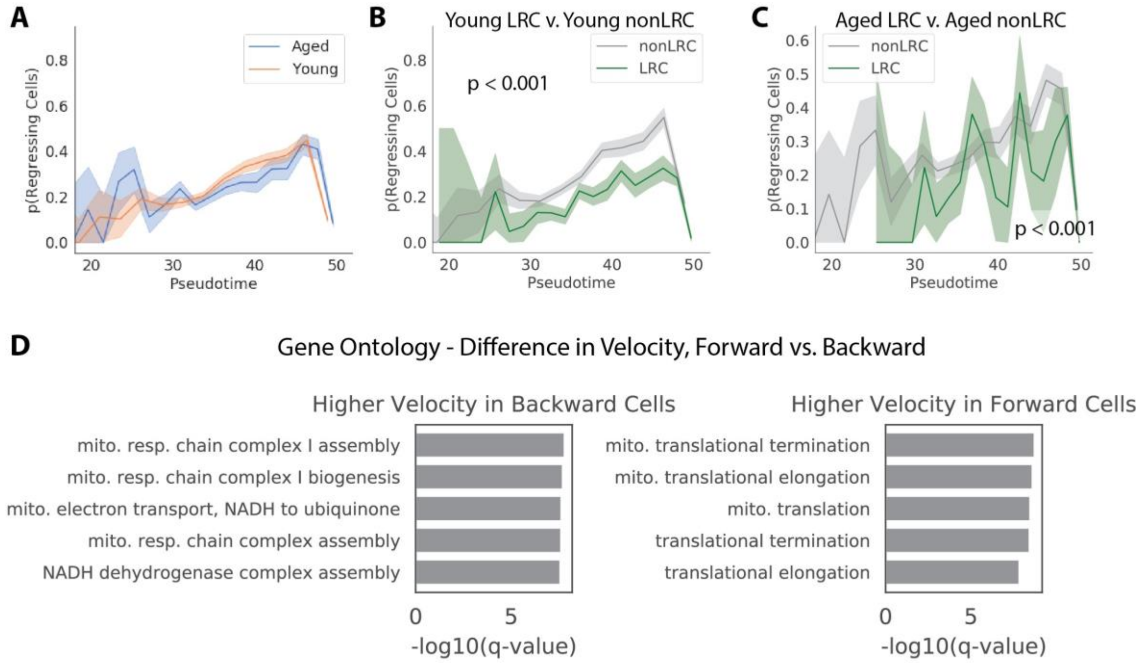

Supp. Fig. S4: **Lineage regression frequency with age (A)** The proportion of "regressing" cells across pseudotime in aged and young MuSCs, revealing little age dependence. Proportions were computed by coarse-graining pseudotime into equally sized bins. **(B)** Proportion of "regressing" cells across pseudotime in young LRCs and nonLRCs. nonLRCs regress more frequently late in the activation process (x^2 test, $p < 0.0001$. **(C)** The difference in lineage regression frequency between LRCs and nonLRCs is also present in MuSCs from aged animals (x^2 test, $p < 0.0001$). **(D)** We computed the difference in velocity for each gene between cells moving "forward" and "backward" in the activation trajectory. Gene Ontology enrichments suggest forward moving cells have higher velocity for protein translation and biogenesis programs, while backward moving cells have higher velocity for mitochondrial oxidative phosphorylation programs.

© 2020. Published by The Company of Biologists under the terms of the Creative Commons Attribution License (https://creativecommons.org/licenses/by/4.0/).

> 7. Figure 6E. The authors may want to elaborate on young and aged MuSC curves crossing-over after 1000 timestep.

We thank the reviewer for highlighting this result.

Our phase simulations update the position of a "phase point," (i.e. a simulated cell) over time. When the phase points reach the edge of the transcriptional space we observe in our experiment (near our most activated cells), they reach a metastable point and do not progress further. Intuitively, this reflects our simulations reaching the most activated point we observe, at which point there isn't any further information about where the simulations might go next. The aged phase points take longer to reach the end of the activation process we observe, which we interpret as slower activation kinetics.
We have amended our Results and Figure Legend to make this point clear to readers. A copy of the addition is pasted below:

Results:

After many time steps, both young and aged phase points reach similar inferred pseudotime locations as they near the edge of our observed pseudotime trajectory (Fig. 6D).

Figure Legend:

Curves cross when young and aged phase points have both reached a steady-state at the end of our observed pseudotime trajectory.

> 8. Figure S4. There is no H panel.

We thank the reviewer for pointing out this mistake. We have corrected the legend.

Reviewer 3 Comments

> In spite of potential enthusiasm, unfortunately the manuscript in its current state is almost entirely descriptive and speculative, while, in the opinion of this reviewer, not offering enough experimental evidence to support claims made in the study. And those claims that are supported by data (related to single cell tracking) support rather than substantially extending work done by others characterizing young and aged muscle stem cell behaviors using time-lapse based clonal studies (examples include: Gilbert, Science, 2010; Cosgrove, Nat Med, 2014, and others).

We appreciate the reviewer's candid feedback.

We believe our key contributions are as follows:

1 - Aged and young MuSCs share a common activation trajectory
We demonstrate that aged and young MuSCs show similar activation trajectories using multiple pheno- typing methods. This result is non-obvious given single endpoint measurements, like those presented in [Gilbert et al., 2010, Cosgrove et al., 2014]. Multiple previous studies have reported transcriptional differ- ences between aged and young MuSCs [Chakkalakal et al., 2012, Bernet et al., 2014, Cosgrove et al., 2014, Sousa-Victor et al., 2014], but it cannot be determined from ensemble measurements if these age-related changes define distinct trajectories of activation. Our single cell measurements provide quantitative measurements of the proportion of variation

© 2020. Published by The Company of Biologists under the terms of the Creative Commons Attribution License (https://creativecommons.org/licenses/by/4.0/).

explained by age-related changes, and show that aged and young MuSCs share a common set of transcriptional states through activation despite the existence of some transcriptional changes with age.

2 - Aged MuSCs activate more slowly than young MuSCs transcriptionally & behaviorally

We provide direct measurements of activation dynamics in young and aged MuSCs using single cell behavior and RNA velocity and show aged MuSCs activate more slowly by both measurements. While time-to-first-division assays have long suggested that aged MuSCs show impaired activation, cell cycle entry is but one of many aspects associated with stem cell activation. It cannot be deduced from those studies alone whether aged MuSCs show an impaired rate of activation (as we find here), or rather if some portion of cells travel down an incorrect activation trajectory (for which we find no evidence).

3 - LRCs activate more rapidly than nonLRCs

Work from our lab previously demonstrated that LRCs have better self renewal and differentiation under transplantation and retain a more primitive fate (Pax7+/MyoG-) when dividing *in vitro*. The assumption, although never directly tested was that these cells divided more slowly. Using orthogonal measures in the present manuscript, we show that LRCs activate more rapidly.

Using single cell RNA-seq, we find that LRCs preferentially occupy more activated transcriptional clusters relative to nonLRCs. Through an EdU incorporation assay, we likewise find that LRCs more rapidly enter the cell cycle. This result is non-intuitive, as some models in the field positive that more regenerative cells tend to activate more slowly. Our data are further consistent with recent reports of other “reserve” stem cell populations that activate quickly [Scaramozza et al., 2019]. Similarly, these results are consistent with work that demonstrates rapidly activating cells provide regenerative benefit [Rodgers et al., 2014].

We also believe the following secondary contributions add to the existing body of knowledge:

- We develop the first accurate classifier of single cell age in MuSCs.
- We likewise develop the first accurate classifier of LRC/nonLRC status in MuSCs.
- We introduce a dynamical systems analytical technique to measure rates of activation using RNA velocity vectors.
- We report for the first time that MuSC activation appears to be a random walk-like process, rather than a smooth progression from quiescence to activation.

> It should also be noted that the manuscript is rather overwhelming to read as it is quite dense and moves between a number of intellectually stimulating, though rather distinct topics and queries, with little depth, but at a substantial speed, and with quite some length.

We thank the reviewer for this honest assessment. We have substantially reworked the text of our manuscript to improve readability while reducing word count.

> The work may be of importance to the community, but in its current state, it is difficult to pinpoint one or two of the speculative statements as ‘key’ and to offer a series of wet lab experiments that would bring it up to a level appropriate for a Development reader.

© 2020. Published by The Company of Biologists under the terms of the Creative Commons Attribution License (https://creativecommons.org/licenses/by/4.0/).

We agree that many different questions in are addressed in our manuscript pertaining to general MuSC activation biology, MuSC aging, and MuSC heterogeneity. We deliberately chose to use investigative methods which are unbiased, measuring many cell behaviors and molecular changes, such that many questions could be addressed with these data. However, we agree that addressing many questions simultaneously can be distracting. We have therefore reworked the text of our manuscript to highlight the contributions we believe are of the most importance.

It is not accurate to state that 'it remains unclear if the transition states and rates of activation are uniform across cells, or how features of this process may change with age' in light of single cell studies by others that clearly show the time to first division is quite varied for young muscle stem cells (Gilbert, 2010) and that with age, there are populations of cells that failed to enter cell cycle and produce a colony in the same time frames observed for young cells (Cosgrove, 2014).

We have amended the abstract text to make our distinctions clear.

The pioneering work of Gilbert 2010 & Cosgrove 2014 indeed highlighted for the first time that young and aged MuSCs have different time-to-first division and clonal outputs. However, cell cycle activity is only one component of the activation process, and it cannot be deduced from this singular read-out if the broader program of MuSC activation is delayed with age or if the transcriptional trajectory is altered. In order to address these latter questions, the transcriptional and behavioral state of young and aged MuSCs must be profiled using unbiased methods, such as those we employ here.

We have amended our Introduction to clarify the importance of contributions from Gilbert 2010 & Cosgrove 2014, while highlighting the remaining unknowns. A sample of these additions is copied below:

In muscle stem cells (MuSCs), the activation process is canonically characterized by expression of *Myod1* [Grounds et al., 1992, Yablonka-Reuveni and Rivera, 1994], loss of *Spry1* and *Pax7*, and entry into the cell cycle [Shea et al., 2010]. Multiple groups have characterized the dynamics of activation at the population level using ensemble assays to measure these molecular markers [Yablonka-Reuveni and Rivera, 1994, Cornelison and Wold, 1997, Fu et al., 2015, Jones et al., 2005, Zhang et al., 2010]. Likewise, it has been reported that aged MuSCs show a delayed time to first division relative to young cells, with fewer aged cells forming colonies *in vitro* [Gilbert et al., 2010, Cosgrove et al., 2014]. These studies have elucidated many of the molecular players and sequences in MuSC activation and shown that aged cells exhibit a delay in at least one activation hallmark (first division time).

Genomics studies have revealed that MuSC activation is a complex process, affecting many aspects of transcription and cell behavior [Liu et al., 2013]. However, it remains unknown how aging affects the progress of activation in MuSCs outside of a small set of molecular markers and binary behavior features (i.e. cell cycle events). While it is known that aged MuSCs display a delayed cell cycle entry for instance, it is unknown if this one feature of cell behavior reflects a broader delay in the activation process across the many transcriptional and cell behavior features involved. Traditional molecular biology tools have also limited investigation to terminal assays, such that activation dynamics in single cells have not been directly observed. In order to disambiguate between the Different Paths and Different Rates models of MuSC aging, we require single cell measurements of activation dynamics that capture a broad set of transcriptional and behavioral features.

© 2020. Published by The Company of Biologists under the terms of the Creative Commons Attribution License (https://creativecommons.org/licenses/by/4.0/).

References

[Bernet et al., 2014] Bernet, J. D., Doles, J. D., Hall, J. K., Tanaka, K. K., Carter, T. A., and Olwin, B. B. (2014). p38 MAPK signaling underlies a cell-autonomous loss of stem cell self-renewal in skeletal muscle of aged mice. *Nat Med*, 20(3):1–9.

[Blau et al., 2015] Blau, H. M., Cosgrove, B. D., and Ho, A. T. V. (2015). The central role of muscle stem cells in regenerative failure with aging. *Nat Med*, 21(8):854–862.

[Chakkalakal et al., 2014] Chakkalakal, J. V., Christensen, J., Xiang, W., Tierney, M. T., Boscolo, F. S., Sacco, A., and Brack, A. S. (2014). Early forming label-retaining muscle stem cells require p27kip1 for maintenance of the primitive state. *Development*, 141(8):1649–1659.

[Chakkalakal et al., 2012] Chakkalakal, J. V., Jones, K. M., Basson, M. A., and Brack, A. S. (2012). The aged niche disrupts muscle stem cell quiescence. *Nature*, 490(7420):355–360.

[Cornelison and Wold, 1997] Cornelison, D. and Wold, B. J. (1997). Single-cell analysis of regulatory gene expression in quiescent and activated mouse skeletal muscle satellite cells. *Dev Biol*, 191(2):270–283.

[Cosgrove et al., 2014] Cosgrove, B. D., Gilbert, P. M., Porpiglia, E., Mourkioti, F., Lee, S. P., Corbel, S. Y., Llewellyn, M. E., Delp, S. L., and Blau, H. M. (2014). Rejuvenation of the muscle stem cell population restores strength to injured aged muscles. *Nat Med*, 20(3):255–264.

[De Micheli et al., 2019] De Micheli, A. J., Fraczek, P., Soueid-Baumgarten, S., Ravichandran, H., De Vlaminck, I., Elemento, O., and Cosgrove, B. D. (2019). Single-cell analysis of the muscle stem cell hierarchy identifies heterotypic communication signals involved in skeletal muscle regeneration. *bioRxiv*, pages 1–42.

[Dell'Orso et al., 2019] Dell'Orso, S., Juan, A. H., Ko, K.-D., Naz, F., Perovanovic, J., Gutierrez-Cruz, G., Feng, X., and Sartorelli, V. (2019). Single cell analysis of adult mouse skeletal muscle stem cells in homeostatic and regenerative conditions. *Development*, 146(12):dev174177.

[Elowitz et al., 2002] Elowitz, M. B., Levine, A. J., Siggia, E. D., and Swain, P. S. (2002). Stochastic gene expression in a single cell. *Science (New York, N.Y.)*, 297(5584):1183–1186.

[Fu et al., 2015] Fu, X., Wang, H., and Hu, P. (2015). Stem cell activation in skeletal muscle regeneration.
Cellular and molecular life sciences : CMLS, 72(9):1663–1677.

[Gilbert et al., 2010] Gilbert, P. M., Havenstrite, K. L., Magnusson, K. E. G., Sacco, A., Leonardi, N. A., Kraft, P., Nguyen, N. K., Thrun, S., Lutolf, M. P., and Blau, H. M. (2010). Substrate elasticity regulates skeletal muscle stem cell self-renewal in culture. *Science (New York, N.Y.)*, 329(5995):1078–1081.

[Grounds et al., 1992] Grounds, M. D., Garrett, K. L., Lai, M. C., Wright, W. E., and Beilharz, M. W. (1992). Identification of skeletal muscle precursor cells in vivo by use of MyoD1 and myogenin probes. *Cell and tissue research*, 267(1):99-104.

[Hansen et al., 2018] Hansen, M. M. K., Desai, R. V., Simpson, M. L., and Weinberger, L. S. (2018). Cytoplasmic Amplification of Transcriptional Noise Generates Substantial Cell-to-Cell Variability. *Cell Systems*, 7(4):384-397.e6.

[Jones et al., 2005] Jones, N. C., Tyner, K. J., Nibarger, L., Stanley, H. M., Cornelison, D. D. W., Fedorov, Y. V., and Olwin, B. B. (2005). The p38alpha/beta MAPK functions as

© 2020. Published by The Company of Biologists under the terms of the Creative Commons Attribution License (https://creativecommons.org/licenses/by/4.0/).

a molecular switch to activate the quiescent satellite cell. *J Cell Biol*, 169(1):105–116.

[Keyes et al., 2016] Keyes, B. E., Liu, S., Asare, A., Naik, S., Levorse, J., Polak, L., Lu, C. P., Nikolova, M., Pasolli, H. A., and Fuchs, E. (2016). Impaired Epidermal to Dendritic T Cell Signaling Slows Wound Repair in Aged Skin. *Cell*, 167(5):1323–1338.e14.

[Kimmel et al., 2018] Kimmel, J. C., Chang, A. Y., Brack, A. S., and Marshall, W. F. (2018). Inferring cell state by quantitative motility analysis reveals a dynamic state system and broken detailed balance. *PLoS computational biology*, 14(1):e1005927–29.

[Lim et al., 2005] Lim, L. P., Lau, N. C., Garrett-Engele, P., Grimson, A., Schelter, J. M., Castle, J., Bartel, D. P., Linsley, P. S., and Johnson, J. M. (2005). Microarray analysis shows that some microRNAs downregulate large numbers of target mRNAs. *Nature*, 433(7027):769–773.

[Liu et al., 2013] Liu, L., Cheung, T. H., Charville, G. W., Hurgo, B. M. C., Leavitt, T., Shih, J., Brunet, A., and Rando, T. A. (2013). Chromatin modifications as determinants of muscle stem cell quiescence and chronological aging. *Cell Reports*, 4(1):189–204.

[Ma et al., 2018] Ma, F., Lin, P., Chen, Q., Lu, X., Zhang, Y. E., and Wu, C.-I. (2018). Direct measurement of pervasive weak repression by microRNAs and their role at the network level. pages 1–12.

[Rodgers et al., 2014] Rodgers, J. T., King, K. Y., Brett, J. O., Cromie, M. J., Charville, G. W., Maguire, K. K., Brunson, C., Mastey, N., Liu, L., Tsai, C.-R., Goodell, M. A., and Rando, T. A. (2014). mTORC1 controls the adaptive transition of quiescent stem cells from G0 to GAlert. *Nature*, 510(7505):393–396.

[Rodgers et al., 2017] Rodgers, J. T., Schroeder, M. D., Ma, C., and Rando, T. A. (2017). HGFA Is an Injury-Regulated Systemic Factor that Induces the Transition of Stem Cells into GAlert. *Cell Reports*, 19(3):479–486.

[Ryall et al., 2015] Ryall, J. G., Dell'Orso, S., Derfoul, A., Juan, A., Zare, H., Feng, X., Clermont, D., Koulnis, M., Gutierrez-Cruz, G., Fulco, M., and Sartorelli, V. (2015). The NAD+-Dependent SIRT1 Deacetylase Translates a Metabolic Switch into Regulatory Epigenetics in Skeletal Muscle Stem Cells. *Cell Stem Cell*, 16(2):171–183.

[Scaramozza et al., 2019] Scaramozza, A., Park, D., Kollu, S., Beerman, I., Sun, X., Rossi, D. J., Lin, C. P., Scadden, D. T., Crist, C., and Brack, A. S. (2019). Lineage Tracing Reveals a Subset of Reserve Muscle Stem Cells Capable of Clonal Expansion under Stress. *Cell Stem Cell*, pages 1–20.

[Shea et al., 2010] Shea, K. L., Xiang, W., LaPorta, V. S., Licht, J. D., Keller, C., Basson, M. A., and Brack, A. S. (2010). Sprouty1 regulates reversible quiescence of a self-renewing adult muscle stem cell pool during regeneration. *Cell Stem Cell*, 6(2):117–129.

[Siegel et al., 2009] Siegel, A. L., Atchison, K., Fisher, K. E., Davis, G. E., and Cornelison, D. D. W. (2009). 3D Timelapse Analysis of Muscle Satellite Cell Motility. *Stem Cells*, 27(10):2527–2538.

[Sousa-Victor et al., 2014] Sousa-Victor, P., Gutarra, S., and Garcia-Prat, L. (2014). Geriatric muscle stem cells switch reversible quiescence into senescence. *Nature*.

[Webster et al., 2016] Webster, M. T., Manor, U., Lippincott-Schwartz, J., and Fan, C.-M. (2016). Intravital Imaging Reveals Ghost Fibers as Architectural Units Guiding Myogenic Progenitors during Regeneration. *Cell Stem Cell*, 18(2):243–252.

[Yablonka-Reuveni and Rivera, 1994] Yablonka-Reuveni, Z. and Rivera, A. J. (1994).

© 2020. Published by The Company of Biologists under the terms of the Creative Commons Attribution License (https://creativecommons.org/licenses/by/4.0/).

Temporal expression of regulatory and structural muscle proteins during myogenesis of satellite cells on isolated adult rat fibers. *Dev Biol*, 164(2):588–603.

[Yoshida et al., 1998] Yoshida, N., Yoshida, S., Koishi, K., Masuda, K., and Nabeshima, Y. (1998). Cell heterogeneity upon myogenic differentiation: down-regulation of MyoD and Myf-5 generates 'reserve cells'. *Journal of Cell Science*, 111(6):769–779.

[Zammit et al., 2006] Zammit, P. S., Relaix, F., Nagata, Y., Ruiz, A. P., Collins, C. A., Partridge, T. A., and Beauchamp, J. R. (2006). Pax7 and myogenic progression in skeletal muscle satellite cells. *Journal of Cell Science*, 119(Pt 9):1824–1832.

[Zhang et al., 2010] Zhang, K., Sha, J., and Harter, M. L. (2010). Activation of Cdc6 by MyoD is associated with the expansion of quiescent myogenic satellite cells. *J Cell Biol*, 188(1):39–48.

Second decision letter

MS ID#: DEVELOP/2019/183855

MS TITLE: Aging induces aberrant state transition kinetics in murine muscle stem cells

AUTHORS: Jacob C. Kimmel, Ara B. Hwang, Annarita Scaramozza, Wallace F. Marshall, and Andrew S. Brack
ARTICLE TYPE: Research Article

I am happy to tell you that your manuscript has been accepted for publication in Development, pending our standard ethics checks.

Reviewer 1

Advance summary and potential significance to field

The authors have significantly improved the original manuscript and have sufficiently addressed all of the concerns mentioned. The revised manuscript is clearer and has framed their results in relation to previous published works with better context.

Comments for the author

A few points:
In Figure 2F, Pax7 is annotated as belonging to gene module 4. Module 4 displays monotonically increase expression (as indicated in the reviewers' response). Yet, the authors say Pax7 has non-monotonic behaviour (in text). Is Pax7 mislabelled on the heatmap? If not, why does it group with the monotonically increasing module #4?
In Figure 5G compared to 5H, the EdU uptake is drastically different. If the Pax3 population is label ed retaining (LRC), the EdU kinetics should be similar. Or similarly, the Pax3- MuSCs (5H) seems to have higher EdU uptake compared to both LRCs and nonLRCs (Figure 5G).

Reviewer 2

Advance summary and potential significance to field

© 2020. Published by The Company of Biologists under the terms of the Creative Commons Attribution License (https://creativecommons.org/licenses/by/4.0/).

The paper provides detailed and comprehensive analysis of the activation kinetics experienced by young and aged muscle stem cells.

Comments for the author

The authors have satisfactorily addressed my comments and should be congratulated for the nice contribution.

© 2020. Published by The Company of Biologists under the terms of the Creative Commons Attribution License (https://creativecommons.org/licenses/by/4.0/).
